# Three-dimensional distribution of amino groups improves perovskite crystallization and defect passivation in high-performance photovoltaics

**DOI:** 10.1038/s41467-026-75990-x

**Published:** 2026-07-25

**Authors:** Xiaoqing Jiang, Guangyue Yang, Bingqian Zhang, Yu Lei, Panyu Wang, Na Shi, Kaiwen Dong, Likai Zheng, Yue Qiang, Shiwei Liu, Zhongjin Shen, Marina Freitag, Chongwen Li, Shuping Pang, Mohammad khaja Nazeeruddin, Xin Guo

**Affiliations:** 1 State Key Laboratory of Photoelectric Conversion and Utilization of Solar Energy, Qingdao Institute of Bioenergy and Bioprocess Technology, Chinese Academy of Sciences, Qingdao New Energy Shandong Laboratory, Shandong Energy Institute, Qingdao, China; 2 College of Chemical Engineering, Qingdao University of Science and Technology, Qingdao, China; 3 School of Electronic Science and Engineering, Southeast University, Nanjing, China; 4 Laboratory of Photonics and Interfaces, École Polytechnique Fédérale de Lausanne, Lausanne, Switzerland; 5 Engineering Technology Research Institute, PetroChina Xinjiang Oilfield Company, Karamay, China; 6 School of Natural and Environmental Sciences, Newcastle University, Newcastle upon Tyne, United Kingdom; 7 School of Integrated Circuits, Southeast University, Wuxi, China; 8 State Key Laboratory of Photoelectric Conversion and Utilization of Solar Energy, Dalian Institute of Chemical Physics, Chinese Academy of Sciences, Dalian, China

**Keywords:** Solar cells, Solar cells

## Abstract

Incorporating organic molecules with diverse functional groups to improve film quality has emerged as a crucial strategy for realizing high-performance perovskite solar cells (PSCs). Nevertheless, the role of spatial distribution of those functional groups in governing passivation efficacy and perovskite crystallization remains insufficiently investigated. Here, we introduce three amino-containing molecules, bis(4-aminophenyl)methane (2APM), tris(4-aminophenyl)methane (3APM) and tetrakis(4-aminophenyl)methane (4APM), featuring distinct spatial distributions of amino groups, into the perovskite precursor solution as in-situ regulators. Among them, 4APM exhibits the strongest interactions with PbI_2_ and formamidinium iodide (FAI) by virtue of its three-dimensional (3D) distribution of amino groups, most effectively suppressing undercoordinated Pb^2+^ defects and enhancing perovskite film crystallinity. As a result, PSCs incorporating 4APM achieve a stabilized power conversion efficiency (PCE) of 26.26%, while retaining over 95% of their initial efficiency after 1000 h of continuous operation at maximum power point under 1-sun illumination in a N_2_ atmosphere at 65 °C. Furthermore, 4APM-based perovskite solar modules (PSMs) with an active area of 14.0 cm^2^ deliver a PCE of 23.16%. Our findings underscore the critical role of functional group’s spatial distribution in the rational design of molecular passivators for perovskite photovoltaics.

## Abstract (web summary)

Organic additives can improve perovskite solar cells, but the effect of functional group arrangement on defect passivation and crystal growth is poorly understood. Jiang et al. compared three amino-containing molecules and found that a 3D four-armed design delivered the best efficiency and stability

## Introduction

Hybrid metal halide perovskite materials have emerged as exceptional light absorbers for next-generation photovoltaics, with certified power conversion efficiencies (PCEs) exceeding 27% over the past decade^1–4^. However, solution-based fabrication of perovskite solar cells (PSCs) inevitably generates undercoordinated ion defects at perovskite film surfaces and grain boundaries, which critically undermines device stability^5–7^. In-situ modification has emerged as a widely adopted strategy in conventional PSCs for suppressing non-radiative charge carrier recombination and enhancing device durability^8,9^. By incorporating functional molecules directly into the perovskite precursor solution, this approach promotes carrier transport and modulates crystallization kinetics, enabling comprehensive passivation of bulk defects throughout the entire film^10,11^.

Incorporating amino-based molecules with tailored functional groups as precursor additives has proven effective in modulating the crystallization kinetics of formamidinium (FA) and cesium (Cs)-based perovskites while efficiently passivating perovskite defects^12–14^. Previous studies have demonstrated that variations in substituent chemistry significantly influence the passivation efficacy of amino-based molecules, with representative examples including long-chain alkyl groups^15,16^, pyridyl groups^17,18^, and aromatic groups^5,19^. Furthermore, increasing the number of amino functional groups within a single molecule has been shown to substantially enhance its overall passivation capability^20^. Nevertheless, the role of the intramolecular spatial distribution of multiple amino groups in governing passivation performance remains poorly understood^21,22^. Therefore, a systematic investigation into the relationship between the intramolecular spatial arrangement of passivating groups and their molecular passivation capacity is critically needed.

**Fig. 1:**
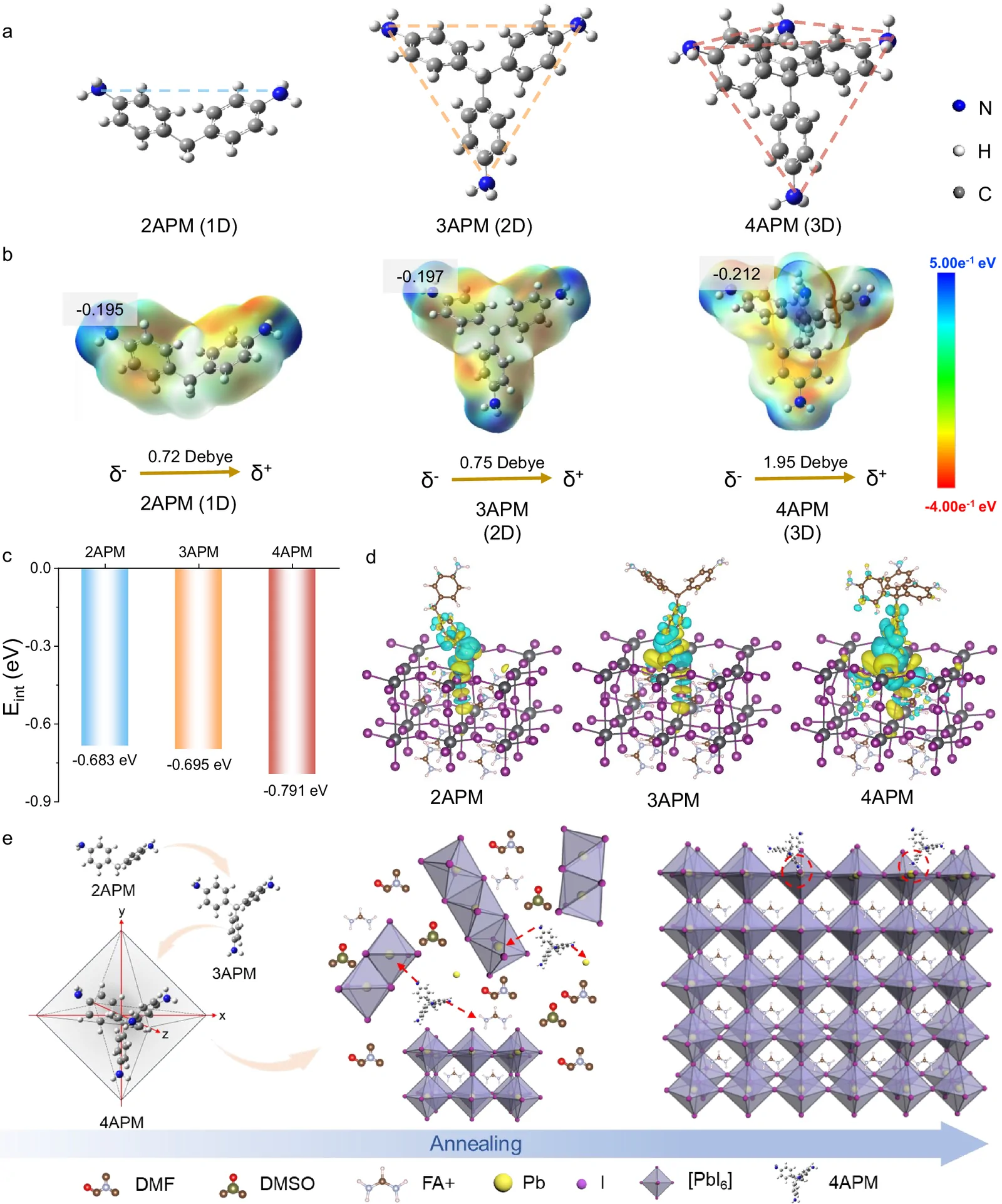
Simulation of molecular characteristics and interactions with perovskite defects. **a** Molecular structures (ball-and-stick models) of bis(4-aminophenyl)methane (2APM), tris(4-aminophenyl)methane (3APM) and tetrakis(4-aminophenyl)methane (4APM) with 1D, 2D and3D distributions of amino groups. **b** Calculated ESP profiles for 2APM, 3APM and 4APM molecules. **c** Interaction energies values of V_I_ defect sites within perovskite films with different additives. **d** Charge difference plot of 2APM, 3APM and 4APM on perovskite surfaces interacting with undercoordinated Pb^2+^. **e** Schematic diagram of using amino-based molecules to regulate perovskite precursors, film crystallization, and defect passivation.

In this work, we investigate the effect of amino group’s spatial distribution in molecular passivators on their passivation capability and PSC performance by introducing bis(4-aminophenyl)methane (2APM), tris(4-aminophenyl)methane (3APM), and tetrakis(4-aminophenyl)methane (4APM), into the perovskite precursor solution (Fig. 1a and Supplementary Fig. 1). Furthermore, another molecule, tetrakis(4-aminophenyl)ethylene (4APE), is also studied as a reference, which possesses the same number yet relatively more planar distribution of amino groups compared to 4APM^23^. The study found that 4APM with its amino groups spatially distributed in a three-dimensional (3D) pattern, relative to the other three molecules, present more obvious enhancements in defect passivation and crystallinity regulation. Meanwhile, the 4APM molecule can optimize the interfacial energy-level alignment, facilitating efficient charge carrier extraction. As a result, 4APM-based PSCs and perovskite solar modules with an active area of 14.0 cm^2^ achieve the best power conversion efficiencies (PCEs) of 26.26% and 23.16%, respectively, while retaining over 95% of their initial efficiency after 1000 h of continuous operation under maximum power point conditions.

## Results

### Molecular interactions with perovskite

To investigate the effect of amino group’s spatial distribution on the passivation capability, density functional theory (DFT) calculations were conducted. As shown in Fig. 1b, the electron cloud density of the amino group increases from −0.195 for 2APM to −0.197 for 3APM and −0.212 for 4APM. Correspondingly, the molecular dipole moment follows the same trend, rising from 0.72 D for 2APM to 0.75 D for 3APM and 1.95 D for 4APM, which indicates that the 3D spatial configuration of amino groups plays a significant role in enhancing the molecular dipole moment, thereby strengthening the electrostatic interaction with perovskite surface and promoting charge redistribution at the molecule/perovskite interface ^24^.

To further quantify additive–perovskite interactions, the Vienna Ab Initio Simulation Package (VASP) was employed to calculate the interaction energies (E_int_) between each additive and undercoordinated I vacancy (V_I_) defects in the perovskite lattice^25,26^. As shown in Fig. 1c, Supplementary Fig. 2 and Supplementary Table 1, 4APM exhibits the strongest interaction energy of −0.791 eV among the three molecules. The formation energies of V_I_ defects before and after modification (Supplementary Figs. 3–4) show that the 4APM-modified perovskite exhibits the higher value of 0.525 eV, compared to other counterparts, indicating highly difficult to form lead ion defects in the 4APM sample. Charge density difference plots further reveal that 4APM facilitates greater electron density sharing toward Pb^2+^ relative to the other additives (Fig. 1d and Supplementary Fig. 5). These DFT results demonstrate that extending the spatial distribution of amino groups progressively enhances the ability of amino-containing additives to suppress defect formation in perovskite films, thereby facilitating the growth of higher-quality perovskite films during annealing (Fig. 1e) ^27^.

**Fig. 2:**
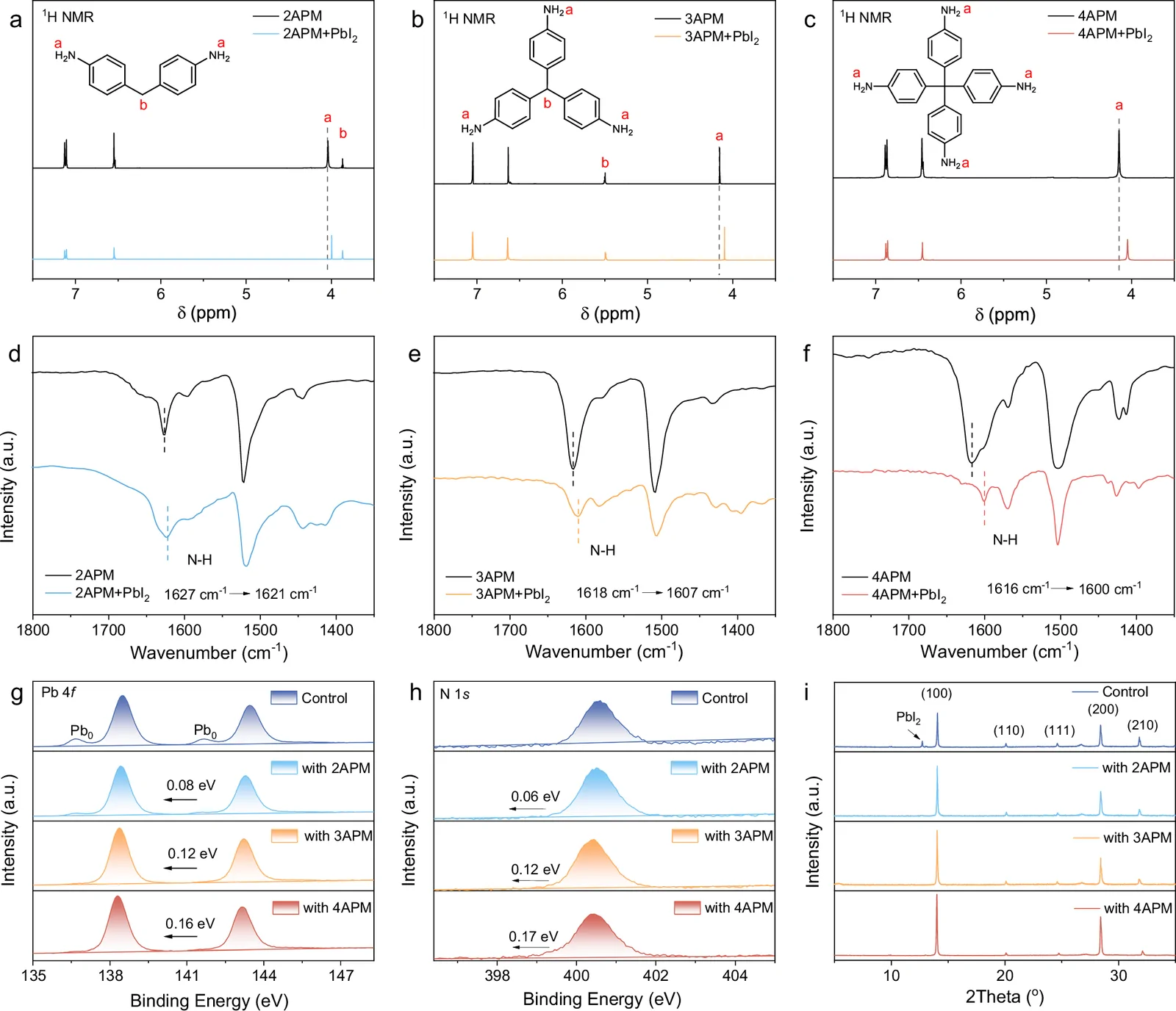
Molecular interactions between additives and perovskite defects. **a**–**c** Proton nuclear magnetic resonance (^1^H NMR) spectra of 2APM, 3APM and 4APM with and without PbI_2_. **d**–**f** Fourier transform infrared spectroscopy (FTIR) spectra of 2APM, 3APM and 4APM films with and without PbI_2_. **g** Pb 4 *f* XPS spectra, **h** N 1 *s* XPS spectra, and **i** XRD patterns of perovskite films without and with 2APM, 3APM and 4APM treatment. (All figures use arbitrary units (arb. units) to denote relative intensity).

To further investigate the interactions between the three molecules and Pb^2+^, proton nuclear magnetic resonance (^1^H NMR) experiments were conducted (Fig. 2a-c). The chemical shifts of –NH_2_ group in 2APM, 3APM and 4APM shifted upfield by 0.04, 0.07 and 0.10 ppm upon addition of PbI_2_, respectively, indicating progressively stronger interaction with Pb^2+^ as the spatial distribution of amino groups increases. Notably, 4APM exhibited the largest upfield shift, confirming its strongest interaction with Pb^2+^ among the three additives^24^. In addition, mixing these molecules with formamidinium iodide (FAI) also induced obvious chemical shifts in the proton signals of –NH_2_ groups, suggesting non-negligible interactions between the amino groups and FAI (Supplementary Fig. 6).

The coordination behavior between these amino-based molecules and Pb^2+^ was further examined by Fourier Transform Infrared Spectroscopy (FTIR) measurements, where each additive was mixed with PbI_2_ at a molar ratio of -NH_2_ group to PbI_2_ of 1:1 (Fig. 2d-f, and Supplementary Fig. 7). After mixing with PbI_2_, the N-H stretching and bending vibrational peaks of all three additives shifted to lower wavenumbers, confirming the coordination between amino groups and Pb^2+^^28^. In specific, the 4APM-based sample exhibited the more pronounced red-shifts (N–H bending: 1616 cm^-1^ → 1600 cm^-1^, ∆ = 16 cm^-1^; N–H stretching: 3429 cm^-1^ → 3413 cm^-1^, ∆ = 16 cm^-1^) than the other two counterparts. These results consistently demonstrate that 4APM with 3D distribution of amino groups possesses the strongest coordination ability with Pb^2+^, in good agreement with the DFT and ^1^H NMR findings.

X-ray photoelectron spectroscopy (XPS) measurements were performed on the additive-treated perovskite films to further elucidate the interactions between the additives and Pb^2+^ defects. As shown in Fig. 2g, the Pb 4 *f* spectra exhibit binding-energy shifts of 0.08 eV for 2APM, 0.12 eV for 3APM and 0.16 eV for 4APM, respectively. The N 1 *s* spectra follow the same trend (Fig. 2h), indicating that more stereoscopic spatial distribution of -NH_2_ groups enhances the defect passivation efficacy^29–31^. Furthermore, the I/Pb ratios of the perovskite films were determined by XPS (Supplementary Fig. 8). The control film exhibits an I/Pb ratio of 3.53, whereas 2APM-, 3APM-, and 4APM-treated films display progressively higher ratios of 3.68, 3.79, and 3.91, respectively. The increased I/Pb ratio in the 4APM-modified film implies more effective suppression of volatile I^0^ species formation, thereby reducing the density of undercoordinated Pb^2+^ defects.

To decouple the influence of amino groups quantity from their spatial distribution, we introduced a more planar amino-based molecule 4APE as a structural control (Supplementary Figs. 9–11). Compared to 4APM, 4APE exhibits a denser electron cloud distribution yet a significantly lower dipole moment, despite containing the same number of amino groups. Both computational and experimental results consistently demonstrate that while the number of amino groups contributes to the interaction energy, the 3D configuration of 4APM enables its four -NH_2_ groups to extend more freely and uniformly into space, in favor of stronger and more spatially distributed interactions with perovskite defects. This stereoscopically structural advantage amplifies the molecular dipole moment of 4APM relative to the more planar 4APE, thereby improving its defect passivation capability beyond what the count of functional groups alone can achieve.

### Molecular effects on film properties

The effect of 2APM, 3APM and 4APM on perovskite crystal growth and film morphology was further investigated. As shown in Fig. 2i and Supplementary Fig. 12, additive-treated perovskite films exhibit more intense (100) X-ray diffraction (XRD) peaks and narrower full-width at half-maximum (FWHM) values relative to the control sample. Notably, both peak intensity and FWHM follow a clear amino spatial distribution-dependent trend, with 4APM-treated perovskite film yielding the highest crystallinity among the three samples. To further corroborate these findings, grazing-incidence wide-angle X-ray scattering (GIWAXS) measurements were conducted. Compared to the control, 2APM- and 3APM-treated perovskite films, the 4APM-treated sample exhibits enhanced diffraction intensity along the out-of-plane (q_z_) direction at q ≈ 1 Å^-1^ (Supplementary Figs. 13 and 14), which corresponds to the (100) crystal plane, indicating stronger preferential orientation, in good agreement with the XRD results discussed above. These results confirm that the introduced additives enhance perovskite crystallinity, which becomes increasingly pronounced with the higher spatial distribution of amino groups. In addition, in-situ XRD measurements (Supplementary Fig. 15) reveal a consistent trend, wherein the additive, particularly 4APM, retards perovskite crystallization kinetics more effectively, promoting the formation of larger perovskite grains and higher-quality films ^32^.

**Fig. 3:**
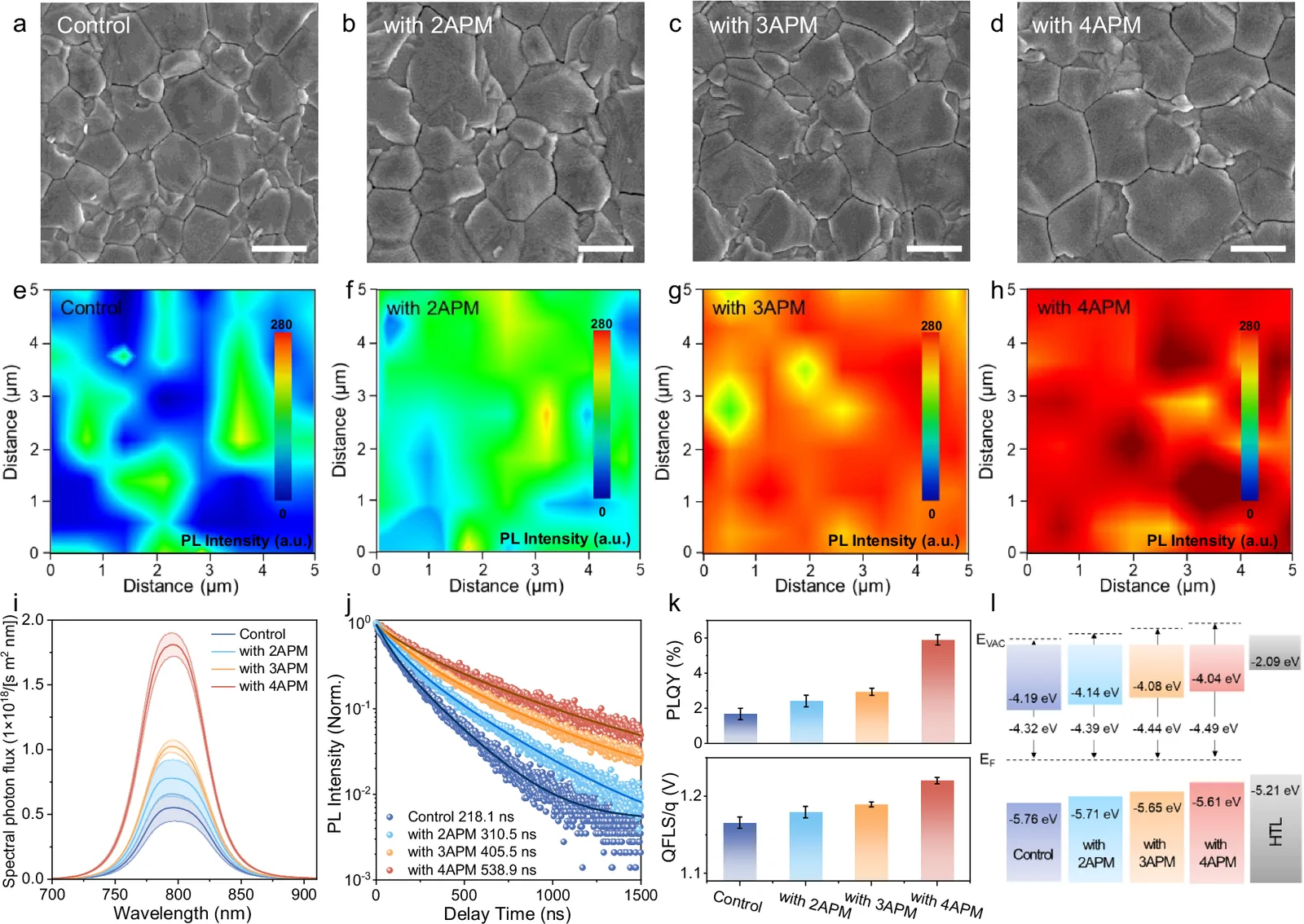
Molecular effects on perovskite film properties. **a**–**d** Top-view SEM images (scale bar: 1 μm), **e**–**h** two-dimensional PL intensity images, **i** Absolute intensity PL spectra of the corresponding devices (solid lines: averaged across three samples for each condition), and **j** TRPL decay of the perovskite films without and with 2APM, 3APM and 4APM. **k** Average PLQYs and quasi-fermi level splitting QFLS/q of the corresponding devices, averaged across three samples for each condition. Error bars represent the standard deviation. **l** Schematic diagrams of energy levels of perovskite films determined from UPS measurements. E_F_ is the Fermi level, and E_VAC_ is the vacuum level.

The surface morphologies of the four perovskite films were examined by scanning electron microscopy (SEM), as shown in Fig. 3a-d. The results clearly show that additive incorporation, particularly the 4APM molecule, significantly promotes perovskite grain growth, yielding an average grain size of 1.95 µm, higher than those of other samples (Supplementary Fig. 16). In addition, cross-sectional SEM measurements further reveal that the 4APM-treated perovskite film exhibits a more continuous grain structure with fewer and less distinct grain boundaries compared to control and those treated with 2APM and 3APM (Supplementary Fig. 17). Atomic force microscopy (AFM) analysis (Supplementary Fig. 18) also reveals a progressive reduction in the surface roughness upon the treatment of 2APM, 3APM, and 4APM. The smoother and more uniform surfaces achieved, most prominently with 4APM, are expected to improve interfacial contact and minimize carrier transport losses, thereby facilitating more efficient charge extraction ^33^.

Two-dimensional (2D) photoluminescence (PL) mapping was conducted to spatially visualize how the spatial distribution of amino groups influences defect passivation capability across the perovskite films. As shown in Fig. 3e-h, the introduction of additives leads to notable improvements in both PL intensity and spatial uniformity, with 4APM-treated films exhibiting the most homogeneous emission distribution^34^. These observations underscore the critical role of more extended distribution of amino groups in mitigating defect states and suppressing nonradiative recombination in perovskite films.

The influence of the additives on charge carrier dynamics was further assessed by steady-state and time-resolved photoluminescence (TRPL) measurements. As illustrated in Fig. 3i, 4APM-modified perovskite device exhibits a remarkable enhancement in PL emission intensity relative to the other devices, indicating most effectively reduced non-radiative recombination. The TRPL lifetime fitting was conducted using the double exponential method. The fast lifetime (τ_1_) represents non radiative recombination on the surface/interface, while the slow lifetime (τ_2_) corresponds to radiative recombination in the bulk phase, as shown in Fig. 3j and Supplementary Table 2^35^. The carrier lifetimes increase with the increased spatial distribution of amino groups, confirming progressive suppression of trap-assisted recombination.

The photoluminescence quantum yield (PLQY) of the corresponding full devices was determined via absolute intensity photoluminescence (AIPL) measurements to further quantify the effect of the spatial distribution of amino groups on film optoelectronic quality. As shown in Fig. 3k, the 4APM-treated device exhibits a markedly higher average PLQY of 5.89%, compared to 2.83% for 3APM, 2.44% for 2APM, and 1.72% for the control, reflecting significant reduction of nonradiative recombination in 4APM-modified device^36^. The quasi-Fermi level splitting (QFLS) was calculated from the PLQY using the equation (Supplementary Note 1)^37^. The extracted QFLS/q values increase progressively from 1.166 V for the control to 1.179 V, 1.189 V, and 1.220 V for 2APM-, 3APM-, and 4APM-modified devices, respectively, confirming that 4APM most effectively suppresses non-radiative recombination and elevates the intrinsic optoelectronic quality of the perovskite films.

Ultraviolet photoelectron spectroscopy (UPS) measurements were conducted to investigate the influence of additives on the energy levels of perovskite layer (Supplementary Fig. 19-20). The valence band of the 4APM-treated film exhibits positive shifts of 40 meV, 100 meV, and 150 meV compared to 3APM-, 2APM-treated and the control films, respectively, resulting in improved energy-level alignment with the HTL layer (Fig. 3l)^38^. These results demonstrate that additive incorporation, especially 4APM, simultaneously passivates defects, enhances carrier lifetime, and optimizes interfacial energy-level alignment, contributing to reduced non-radiative recombination and improved charge transport throughout the device ^39^.

**Fig. 4:**
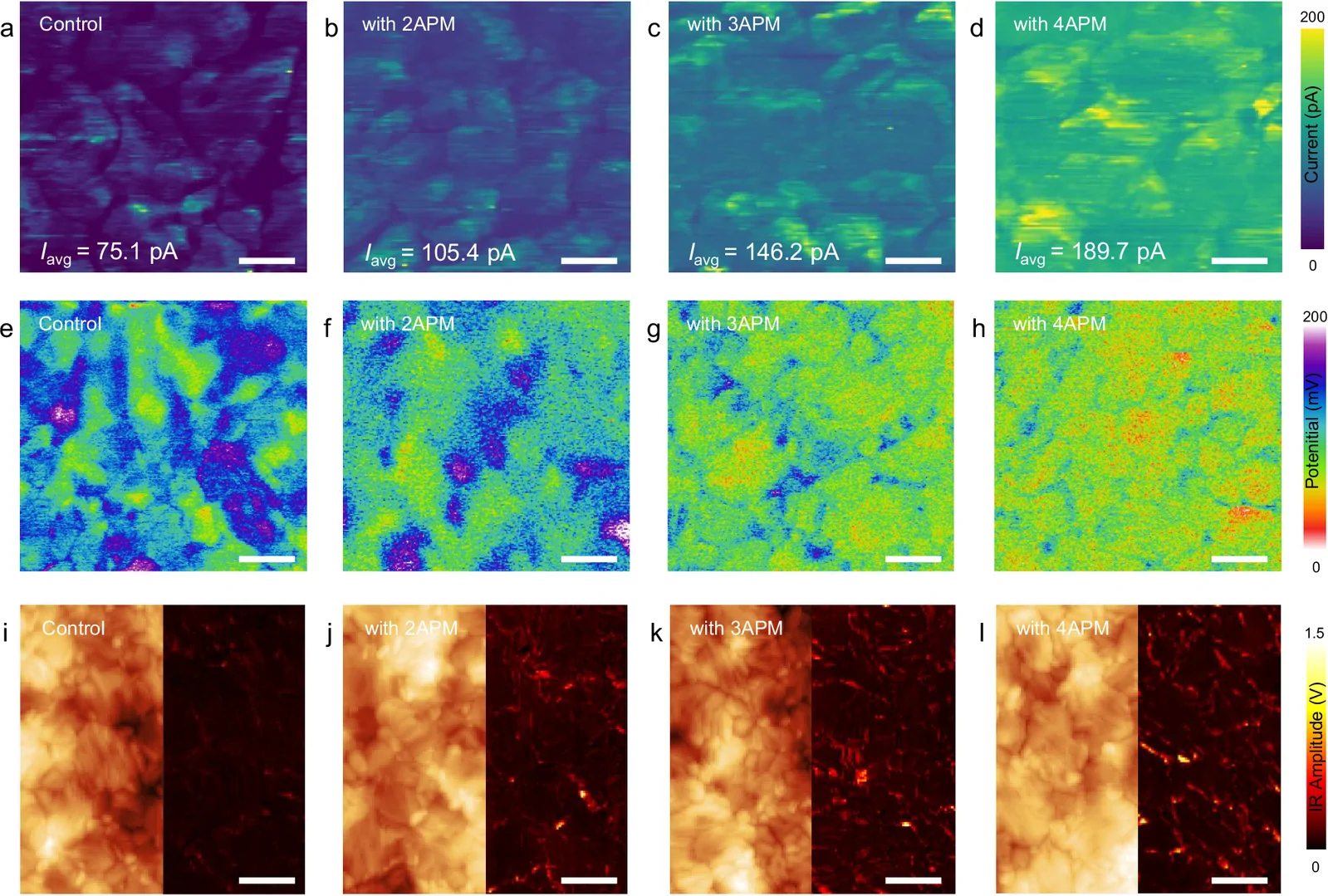
Electrical characteristics of perovskite films. **a**–**d** Conductive AFM current mapping images (I_avg_ represents the average current across the images, scale bar: 1 μm), **e**–**h** KPFM images (scale bar: 1 μm), and **i**–**l** AFM-IR (benzene signals corresponding to additives at 1519 cm^−1^ (2APM), 1508 cm^−1^ (3APM), 1502 cm^−1^ (4APM), scale bar: 2 μm) images of Control, 2APM-, 3APM- and 4APM-treated perovskite films.

Conductive AFM was further employed to verify the effect of the additives on local charge transport within the perovskite films (Fig. 4a-d). Upon additive incorporation, the average current increases systematically with the spatial distribution of amino groups, from 75.1 pA for the control to 105.4 pA for 2APM, 146.2 pA for 3APM, and 189.7 pA for 4APM, respectively, confirming progressively enhanced film conductivity. To further validate the additive-induced changes in the surface electronic structure, Kelvin probe force microscopy (KPFM) was employed to map the surface potential distribution of the perovskite films (Fig. 4e-h). Notably, the large surface potential fluctuations associated with grain boundaries in the untreated film are nearly eliminated after 4APM treatment, yielding a highly uniform surface potential distribution. This improvement can be attributed to the surface dipoles introduced by 4APM, which suppress potential fluctuations and reduce surface defect density^40–43^. Furthermore, the contact potential difference (CPD) decreases progressively with increasing the spatial distribution of amino groups, from 189.3 mV for the control to 126.5 mV, 83.4 mV, and 37.7 mV for 2APM-, 3APM-, and 4APM-modified films (Supplementary Fig. 21), respectively, reflecting an increasingly favorable surface electronic environment for charge extraction.

Time-of-flight secondary ion mass spectrometry (TOF-SIMS) measurements were conducted to investigate the distribution of the additives within the perovskite films. Depth-profile analysis reveals that the additive molecules are partially enriched at the upper surface of the perovskite layer (Supplementary Fig. 22), owing to the synergistic effects of physical segregation during perovskite crystallization and molecular chemical affinity for surface defects, which favors effective passivation of undercoordinated Pb^2+^ defects at the surface and grain boundaries of perovskite films^44,45^. Atomic Force Microscopy-based Infrared Spectroscopy (AFM-IR) was further employed to examine the surface properties of the perovskite thin films. Infrared imaging at 1519 cm^-1^, 1508 cm^-1^, and 1502 cm^-1^, corresponding to the characteristic aromatic C–H bending signals of 2APM, 3APM, and 4APM, respectively (Supplementary Fig. 23), reveals that the additive-treated perovskite films exhibit significantly stronger and more spatially homogeneous benzene ring signals compared to the control (Fig. 4i–l), confirming uniform distribution of the molecular additives across the perovskite surface. These results demonstrate that the incorporated additives, particularly 4APM, play a crucial role in promoting perovskite crystallization, suppressing defect formation and improving charge transport throughout the film ^46^.

### Photovoltaic performance and device stability

**Fig. 5:**
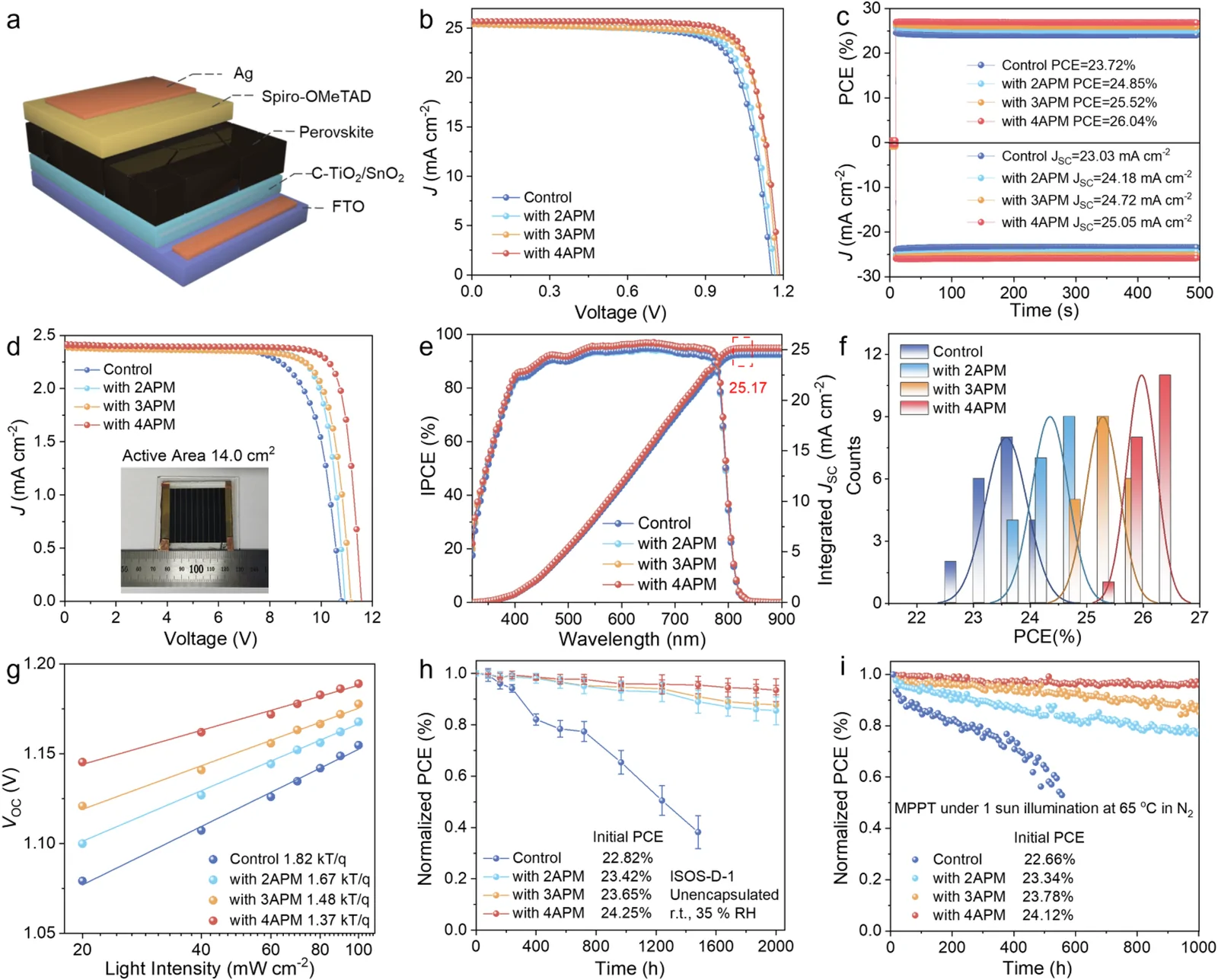
Photovoltaic performance and device stability. **a** An illustration of the device architecture of PSCs. **b** Reverse *J*-*V* curves of the PSCs without and with 2APM, 3APM and 4APM. **c** Time-dependent PCE of the PSCs measured at the maximum power point. **d** *J*-*V* curves of the perovskite solar modules without and with 2APM, 3APM and 4APM. Inset: Image of the PSM. **e** IPCE spectra and corresponding integrated short-circuit current density (*J*_SC_) of the PSCs. **f** Histogram of average PCE values of 20 devices without and with 2APM, 3APM and 4APM. **g** The light-intensity dependence of *V*_*OC*_ for PSCs. The ideality factor is calculated from the slope of the linear fit of the semilogarithmic plot; *K*, Boltzmann constant; *T*, Kelvin temperature; *q*, electron charge. **h** PCE evolution of the unencapsulated devices stored in dark and ambient environments (35% RH) for 2000 h. Error bars represent the standard deviation from 20 independent measurements. **i** MPPT of the unencapsulated devices under simulated illumination at 65 ^o^C in N_2_. (the test based on the device structure of FTO/C-TiO_2_/SnO_2_/perovskite/PTAA/Au).

To investigate the influence of molecular additives on device performance, PSCs with the architecture FTO/C-TiO_2_/SnO_2_/perovskite/Spiro-MeOTAD/Ag were fabricated (Fig. 5a). Current density-voltage (*J*-*V*) curves measured across a range of additive concentrations are shown in Supplementary Fig. 24, with the corresponding photovoltaic (PV) parameters summarized in Supplementary Tables 3-5. The optimal additive concentration was determined to be 0.1 mg/mL for all three molecules. The *J*-*V* curves of the champion devices under AM 1.5 G illumination are presented in Fig. 5b, and the key PV data are listed in Table 1. The control device yields a PCE of 24.04%, with an open-circuit voltage (*V*_OC_) of 1.155 V, a short-circuit current density (*J*_SC_) of 25.42 mA cm^-2^, and a fill factor (*FF*) of 81.87%. Incorporation of additives 2APM and 3APM enhances the device performance, achieving PCEs of 24.74% and 25.67%, respectively. Notably, 4APM-based device delivers the highest PCE of 26.26%, with a *V*_OC_ of 1.187 V, *J*_SC_ of 25.72 mA cm^-2^, and FF of 85.95%. The progressive enhancement in *V*_OC_ across the additive-treated devices can be attributed to the synergistic effects of suppressed non-radiative recombination and optimized interfacial energy-level alignment^47^. Under steady-state maximum power point tracking over 500 s, the 4APM-based device maintains a stabilized PCE of 26.04%, the highest among all tested devices (Fig. 5c). Furthermore, the 4APM-modified device exhibits a negligible hysteresis of 0.7%, compared to 1.2% for 3APM, 1.8% for 2APM, and 2.3% for the control (Supplementary Fig. 25 and Supplementary Table 6). Perovskite solar modules (PSMs) with an active area of 14.0 cm^2^ were further fabricated to assess scalability; the *J-V* curves of the PSMs with different additives are shown in Fig. 5d. The control PSM shows a PCE of 19.30%, with a *V*_OC_ of 10.77 V, *J*_SC_ of 2.376 mA cm^-2^, and FF of 75.61%. While 2APM- and 3APM-treated PSMs achieve higher PCEs of 20.52% and 22.05%, respectively. Remarkably, the 4APM-modified PSM delivers the highest efficiency of 23.16%, with a *V*_OC_ of 11.56 V, *J*_SC_ of 2.406 mA cm^-2^, and FF of 83.23% (Supplementary Table 7), demonstrating the effective scalability of the modification strategy with the 3D amino distribution.

**Table 1:** Photovoltaic parameters of PSCs without and with 2APM, 3APM and 4APM under 1 sun illumination

Sample	*V*_*oc*_ (V)	*J*_*sc*_ (mA cm^-2^)	*FF* (%)	PCE (%)
Control	1.155	25.42	81.87	24.04
with 2APM	1.168	25.55	82.91	24.74
with 3APM	1.178	25.67	84.88	25.67
With 4APM	1.187	25.72	85.95	26.26

Incident photon-to-electron conversion efficiency (IPCE) spectra of all devices are shown in Fig. 5e. The integrated current densities of the control, 2APM-, 3APM-, and 4APM-treated devices are 24.51, 24.65, 24.88, and 25.17 mA cm^-2^, respectively, which are consistent with the *J*_SC_ values obtained from *J-V* measurements (Table 1). The improved photovoltaic performance of 4APM-based devices was further corroborated by statistical analysis of the PV parameters across 20 independently fabricated devices (Fig. 5f, Supplementary Fig. 26 and Supplementary Table 8). Consistently, the ideality factors extracted from voltage response fitting decrease systematically with increasing the spatial distribution of amino groups, from 1.82 for the control to 1.67, 1.48, and 1.37 for 2APM-, 3APM-, and 4APM-based devices (Fig. 5g), respectively, confirming progressively reduced trap-assisted nonradiative recombination^48^. The trap state density in perovskite films was quantitatively assessed using the space-charge-limited current (SCLC) method. As shown in Supplementary Fig. 27, the pristine film exhibits a trap state density of 9.39 × 10^15 ^cm^-3^, which decreases progressively to 7.12 × 10^15 ^cm^-3^, 5.05 × 10^15 ^cm^-3^, and 3.71 × 10^15 ^cm^-3^ upon treatment with 2APM, 3APM, and 4APM, respectively, demonstrating the best defect passivation capability of 4APM.

To further investigate the impact of amino distribution on film stability, XRD measurements were conducted to monitor the phase stability of perovskite films with and without additives upon exposure to ambient air at ~35% relative humidity (RH). As shown in Supplementary Fig. 28, the control film exhibits a weak *δ*-FAPbI_3_ peak after 1000 h, which significantly intensifies after 2000 h. 2APM- and 3APM-treated films show negligible *δ*-FAPbI_3_ and PbI_2_ peaks after 1000 h, which, however, intensify upon further aging to 2000 h. In contrast, the 4APM-incorporated film demonstrates markedly superior phase stability, maintaining a negligible *δ*-FAPbI_3_ peak even after 2000 h of exposure, confirming the exceptional moisture-resistance conferred by the 3D amino group distribution. Furthermore, long-term storage stability of unencapsulated devices was evaluated under 35% RH. The 4APM-modified PSCs exhibit the best durability, retaining 93% of their initial PCE after 2000 h of aging (Fig. 5h), substantially outperforming the 2APM- (85%) and 3APM-based (88%) devices over the same period, and the control device, which retains only 38% of its initial PCE after 1450 h. Under maximum power point (MPP) tracking conditions at 65 ^o^C in a nitrogen (N_2_) atmosphere (Fig. 5i), the unencapsulated 4APM-based device maintains 95% of its initial efficiency after 1000 h under continuous illumination, while the control, 2APM-, and 3APM-modified devices retain only 52%, 77%, and 86% of their initial PCEs, respectively. Furthermore, we investigated the operational stability of the encapsulated PSM under continuous light illumination at 65 °C in ambient air (Supplementary Fig. 29). After 600 h, the 4APM-treated PSM retained 92% of its initial PCE, whereas the control device lost 30% of its initial PCE, further underscoring the superior long-term operational stability imparted by the modification strategy based on the 3D distribution of passivation groups.

## Discussion

In summary, we have systematically investigated the effect of amino group’s spatial distribution in molecular passivators on photovoltaic performances. Integrating DFT calculations with comprehensive experimental characterizations, we demonstrate that increasing the spatial distribution of amino groups progressively enhances defect passivation efficacy, suppresses non-radiative recombination, facilitates interfacial charge transport, and improves perovskite film crystallinity, driving systematic improvements in device performances. The optimized PSC incorporating 4APM with the 3D distribution of amino group achieves a stabilized PCE of 26.26%, while the corresponding PSM with an active area of 14.0 cm^2^ delivers a PCE of 23.16%. Furthermore, 4APM-based unencapsulated devices retain 95% of their initial PCE after 1000 h of continuous MPP operation at 65 °C in a N_2_ atmosphere, and 93% after 2000 h of dark storage under ambient conditions, demonstrating exceptional long-term operational and environmental stability. This work establishes the spatial distribution of functional groups as a critical design parameter for molecular additives, offering a clear and generalizable strategy for the development of high-efficiency and stable perovskite photovoltaics.

## Methods

### Materials

Formamidinium iodide (FAI), methylammonium lead bromide (MAPbBr_3_), cesium iodide (CsI), methylammonium chloride (MACl), lithium bis(trifluoromethanesulfonyl) imide (Li-TSFI), 2,2’,7,7’-tetrakis[N,N-di(4-methoxyphenyl)amino]-9,9’-spirobifluorene (Spiro-OMeTAD) (99.8%), Poly[bis(4-phenyl)(2,4,6-trimethylphenyl)amine] (PTAA), 4-tert-butylpyridine and lead iodide (PbI_2_), 4-tert-butylpyridine (tBP), SnCl_4_ were purchased from Xi’an Polymer Light Technology Corp in China. SnO_2_ colloid precursor (tin (IV) oxide) was purchased from Alfa Aesar. N, N-Dimethylformamide (DMF), dimethyl sulfoxide (DMSO), isopropyl alcohol (IPA), chlorobenzene (CB), ethyl acetate (EA), acetonitrile (ACN), bis(4-aminophenyl)methane (2APM), tris(4-aminophenyl)methane (3APM), tetrakis(4-aminophenyl)methane (4APM) and tetrakis(4-aminophenyl)ethylene (4APE) were purchased from Shanghai Macklin Biochemical Co., LTD. All chemicals were used as received without further purification.

### Device fabrication

The FTO-coated glass was cleaned via sequential sonication (30 min for each) with detergent once, deionized water twice, and ethanol for twice. Then the cleaned substrate was treated with UV/O_3_. A compact layer of TiO_2_ was deposited by atomic layer deposition (ALD) and annealed in ambient air at 500 °C for 30 min. Anhydrous SnCl_4_ and deionized water were mixed in a volume ratio of 1: 1.75 and aged at 25 ^o^C for a week, and the solution changed from a clear and transparent solution to a white colloidal solution (SnO_2_). The SnO_2_ electron transport layer was deposited by spin-coating at 3000 rpm for 30 s, and annealed at 180 °C for 30 min. The perovskite solution ((FA_0.95_Cs_0.05_) PbI_3_)_0.975_(MAPbBr_3_)_0.025_) was prepared by mixing 705.3 mg of PbI_2_, 228.8 mg of FAI, 18.2 mg of CsI, 33.67 mg of MACl and 18.2 mg of MAPbBr_3_ in mixed DMF/DMSO solvent system (v: v = 9:1). The amounts of bis(4-aminophenyl)methane (2APM), tris(4-aminophenyl)methane (3APM), and tetrakis(4-aminophenyl)methane (4APM) added were from 0.1-0.5 mg/mL. The perovskite solution was spin-coated on the SnO_2_ substrates in a two-step at 1000 rpm/10 s and 4000 rpm/30 s. In the last 15 s of the second step, 300 µl of the anti-solvent anisole was dropped at a constant rate, after spin coating was completed, the perovskite film was annealed at 100 °C for 40 min. Then the hole transport layer was prepared by mixing Spiro-OMeTAD (72.3 mg), 4-tert-butylpyridine (28.8 µl), and Li-TFSI (17.5 µl) solution (520 mg of Li-TFSI in 1 ml of acetonitrile) in chlorobenzene (1 ml). The prepared Spiro-OMeTAD solution (40 µl) was deposited on perovskite films by spin-coating (3000 rpm, 30 s). Finally, 80 nm thick Ag electrodes were thermally evaporated under vacuum to complete the device fabrication. For devices in 85° thermal stability tests, Spiro-OMeTAD was replaced with PTAA (PTAA (20 mg), preparation of PTAA solution by mixing 4-tert-butylpyridine (10 µl) and Li-TFSI (15 µl) solution (170 mg Li-TFSI in 1 mL acetonitrile) in chlorobenzene (1 mL)). Finally, 80 nm thick Au electrodes were thermally evaporated under vacuum to complete the device fabrication.

### Module fabrication

P1 (19 μm wide) lines etching process was pre-patterned on FTO glass (5 cm × 5 cm) with a 1064 nm fiber laser (Han’s laser). The laser power ratio, laser duty cycle, and laser frequency were 30%, 5%, and 50 kHz, respectively. Then, patterned FTO substrates were cleaned and treated by UV Ozone Cleaner (Ossila) for 15 min. The SnO_2_, Perovskite, and Spiro-OMeTAD layers were prepared with the same procedure as presented. For the 160 μm-wide P2 lines etching process, the laser used was a 532 nm laser with a laser power ratio of 65%, a laser duty cycle of 5%, and a laser frequency of 100 kHz. 80 nm thick Au electrodes were thermally evaporated under a vacuum to complete the fabrication of the modules. Finally, 78 μm-wide P3 lines etching used the same laser as P2 with a laser power ratio of 50%, a laser duty cycle of 5%, and a laser frequency of 100 kHz. P4 is an etching procedure for cleaning the edge of the modules; the laser used in P4 is the same as P1 with a laser power ratio of 40%, a laser duty cycle of 10%, and a laser frequency of 100 kHz.

### Computational details

The ESP profiles are performed using the Gaussian 16 W software package. The geometry optimization and frequency calculation are carried out using the B3LYP functional with the LANL2DZ basis set. The structure is fully optimized, and a frequency calculation is performed to ensure that the optimized geometry corresponds to a true minimum (no imaginary frequencies). The single-point energy calculations are also carried out at the same level of theory. The charge distribution is analyzed based on the population analysis method, and the electrostatic potential is calculated and visualized to examine the distribution of the charge in the molecular system. The convergence criterion for the geometry optimization is set to 10⁻⁴ a.u. for the energy change, and the maximum force is set to 0.00045 a.u. to ensure accurate results. In the Gaussian input file, fix the position of molecules using the NoOpt keyword to prevent geometric optimization during the calculation process.

The interaction energies and the charge difference plot are implemented using the Vienna ab initio Simulation Package (VASP). We calculated the interaction energies of three molecules on the FAPbI_3_ (100) surface and introduced I vacancies to create undercoordinated Pb^2+^ sites, simulating the typical dominant trap states. The electronic exchange-correlation interactions were described using the Perdew-Burke-Ernzerhof (PBE) functional method under the generalized gradient approximation (GGA-PBE). In all calculations, the plane-wave cutoff energy for electronic wavefunctions was set to 400 eV. The Brillouin zone sampling employed a (2 × 2 × 1) Monkhorst-Pack k-point mesh for geometric structure relaxation. To avoid interactions between adjacent layers, a vacuum layer of 20 Å was set along the z-axis. The dipole moment correction function remained enabled. The van der Waals forces in the structure were corrected using Grimme’s DFT-D3 method.

### Materials characterization

Scanning electron microscopy (SEM, S-4800, Hitachi) and atomic force microscopy (AFM, Bruker Dimension Fast Scan) with peak-force tapping mode using silicon tips (OTESPA, Bruker) were used to characterize the morphology of the perovskite films. Fourier transform infrared (FTIR) spectroscopy was obtained from the instrument Thermo, Nicolet 6700. The resolution of the instrument is 4 cm^-1^. The UV-Vis absorption spectra were measured by a U4100 spectrophotometer (Hitachi). X-ray diffraction (XRD) was performed in regular θ-2θ scanning mode using X-ray diffractometer (PANalytical, CuK_a1_: λ = 0.154056 nm) with scan rate 10°min^-1^. The photoluminescence (PL) signal was recorded using a Horiba Jobin Yvon system, while the time-resolved PL decay profiles were measured using a Picoharp 300 with time-correlated single-photon counting capabilities. Kelvin probe force microscopy (KPFM) was implemented based on a Bruker Dimension V SPM system. The equipped AFM tip was Pt/Ir coated, with ～60 kHz resonance frequency, purchased from Bruker. Ultraviolet photoelectron spectroscopy (UPS) was performed on a photoelectron spectrometer (ESCALAB 250Xi, Thermo Fisher Scientific). The XPS test was carried out with a photoelectron spectrometer model of ESCA LAB 250Xi from Thermo Fisher Scientific in the United States. Compositional depth profiling of perovskite films was carried out using a TOF-SIMS 5 system from IONTOF, which was operated in the spectral mode and using a 30-keV Bi^3+^ primary ion beam with an ion current of 0.8 pA. Photoluminescence quantum yield (PLQY) was measured using an integrating sphere (Fluorolog, Horiba JobinYvon), an Andor Kymera 193i spectrograph, and a 660 nm continuous-wave laser (OBIS, Coherent) set at 1-Sun equivalent photon flux (1.1 µm beam full-width half-maximum, 632 µW); photoluminescence was collected at normal incidence using a 0.1 NA, 110 µm-diameter optical fiber. For the absolute spectral calibration of the PLQY measurement system, we used a radiometrically calibrated halogen lamp (HL-3 plus CAL from Ocean Optics). PL mapping was measured with a laser scanning confocal microscope (Enlitech, SPCM-1000) equipped with a 470-pulse laser and galvo-based scanner. The objective was ×50 with numerical aperture of 0.8; for this objective, the confocal pinhole was set to 50 μm size. The detector was a photo-multiplier tube (Hamamatsu) with a photon-counting module.

### Device characterization

A 2400 Source Meter (Keithley, USA) was used to measure the *J-V* curve of the device at a simulated AM 1.5 G 100 mW/cm^2^ intensity (Oriel Sol3A Class AAA, Newport, USA) with a scan rate 0.1 V/s (1.3 V to −0.1 V). The cells with an active area of 0.25 cm^2^ (0.5 cm × 0.5 cm) were masked with a black metal mask with an area of 0.09 cm^2^. The external quantum efficiency (EQE) measurement was recorded using certified incident photon to current conversion efficiency equipment from Enlitech (Taiwan). The MPP stability tests were performed using a stability setup (LC Auto-Test 24, Shenzhen Lancheng Technology Co., Ltd.). In the test, all PSCs were without encapsulation, and tested under continuous light illumination and maximum power point tracking. The light source consisted of an array of white LEDs powered by a constant current. The LED type is MG-A200A-AE with an emission spectrum of 400-750 nm. Equivalent sun intensities were calibrated using a calibrated Si-reference cell. During aging, the PSCs were masked and placed inside a sample holder purged with continuous N_2_ flow. *J-V* curves with reverse voltage scans were recorded every 6 h during the whole operational test.

### Reporting summary

Further information on research design is available in the Nature Portfolio Reporting Summary linked to this article.

## Supplementary information

Supplementary Information

reporting summary

Transparent Peer Review file

## Source data

Source data

## Data Availability

The data that support the findings of this study are available within the article and its supplementary information. Correspondence and requests for materials should be addressed to the corresponding authors (Z.S., C.L., M.K.N. and X.G.). Source data are provided with this paper.
