# Supplementary material for: Three-dimensional distribution of amino groups improves perovskite crystallization and defect passivation in high-performance photovoltaics
Source: Nat Commun. 2026 Jul 25;17:9077. doi: 10.1038/s41467-026-75990-x (PMC13518884; doi:10.1038/s41467-026-75990-x)
Supplement: Supplementary file 1 [file 41467_2026_75990_MOESM1_ESM.pdf]

Supplementary Information

Table of Contents

Supplementary Note 1: The calculation of quasi-Fermi level splitting (QFLS) 3

Supplementary Fig. 1. Molecular structure of 2APM, 3APM and 4APM..4

Supplementary Fig. 2. DFT calculations of the interaction energy (E_{int}) of 2APM, 3APM, and 4APM with V_I defect sites on the surface of perovskite. 5

Supplementary Fig. 3. DFT calculations of the formation energies of V_I defect sites within perovskite without (a) and with 2APM (b), 3APM (b) and 4APM (d) 6

Supplementary Fig. 4. Formation energies values of V_I defect sites within perovskite films with different additives 7

Supplementary Fig. 5. Longitudinal integration image of differential charge density. 8

Supplementary Fig. 6. ^{1}H NMR spectra of (a) 2APM-FAI, (b) 3APM-FAI and (c) 4APM-FAI. 9

Supplementary Fig. 7. Fourier-transform infrared spectroscopy (FTIR) spectra of (a) pure 2APM and 2APM-PbI_2 films, (b) pure 3APM and 3APM-PbI_2 films, (c) pure 4APM and 4APM-PbI_2 films 10

Supplementary Fig. 8. I/Pb ratios extracted from Fig. 2g 11

Supplementary Fig. 9. Properties of 4APE molecules 12

Supplementary Fig. 10. Molecular simulation calculations of 4APE 13

Supplementary Fig. 11. Effects of molecular interactions with perovskites. 14

Supplementary Fig. 12. The full width at half maximum (FWHM) of the (100) diffraction peak................. 15

Supplementary Fig. 13. GWIAXS patterns of perovskite films without (a) and with (b) 2APM, (c) 3APM and (d) 4APM passivation. 16

Supplementary Fig. 14. The plots of perovskite film of (100) facet along the ring at the range about 0.9-1.05 $Å^{-1}$.. 17

Supplementary Fig. 15. In situ XRD patterns of the crystallization process of perovskite films without (a) and with 2APM (b), 3APM (b) and 4APM (d) over time.. 18

Supplementary Fig. 16. Domain size distribution of perovskite films without (a) and with 2APM (b), 3APM (b) and 4APM (d)... 19

Supplementary Fig. 17. Cross-sectional SEM images of perovskite films without (a) and with (b) 2APM, (c) 3APM and (d) 4APM, respectively. 20

Supplementary Fig. 18. Topography AFM images of perovskite films without (a) and with 2APM (b), 3APM (b) and 4APM (d)... 21

Supplementary Fig. 19. UPS data of perovskite films without (a) and with 2APM (b), with 3APM (c) and with 4APM (d). 22

Supplementary Fig. 20. UPS data of Spiro-OMeTAD. 23

Supplementary Fig. 21. CPD variation of perovskite films without and with 2APM, 3APM and 4APM along the line scan in KPFM images 24

Supplementary Fig. 22. TOF-SIMS depth profiles of perovskite films without (a) and with 2APM (b), 3APM (c) and 4APM (d) deposited on silicon substrates 25

Supplementary Fig. 23. FTIR spectra of pristine 2APM (a), 3APM (b) and 4APM (c) films. Dashed lines indicate the peak positions of benzene bond vibration signals 26

Supplementary Fig. 24. *J-V* curves of perovskite solar cells incorporating different concentrations of the 2APM, 3APM and 4APM. 27

Supplementary Fig. 25. Forward *J-V* curves of the PSCs without and with 2APM, 3APM and 4APM.......... 28

Supplementary Fig. 26. The statistical distribution of PCE (a), J_{SC} (b), V_{OC} (c) and *FF* (d) of PSCs from 20 devices for each condition. 29

Supplementary Fig. 27. Space charge limited current (SCLC) curve of hole-only devices; V_{TFL} values indicate trap density variation. 30

Supplementary Fig. 28. Aged XRD of perovskite thin films 31

Supplementary Fig. 29. Operational stability of the encapsulated PSMs under 1-sun MPPT conditions at 65 °C in ambient air. 32

Supplementary Table 1. The interaction energies between molecules and perovskite surface defects. 33

Supplementary Table 2. TRPL decay lifetimes of perovskite films 34

Supplementary Table 3. Performance of perovskite solar cells at different concentrations (2APM).............. 35

Supplementary Table 4. Performance of perovskite solar cells at different concentrations (3APM).............. 35

Supplementary Table 5. Performance of perovskite solar cells at different concentrations (4APM).............. 35

Supplementary Table 6. Photovoltaic parameters for forward and reverse scanning of PSCs without and with 2APM, 3APM and 4APM under 1 sun illumination.. 36

Supplementary Table 7. Summary of device performance of perovskite solar modules without and with 2APM, 3APM and 4APM. 37

Supplementary Table 8. Comparison of the photovoltaic performance of 20 devices for Control, 2APM-, 3APM- and 4APM-treated samples.. 38

Supplementary Note 1: The calculation of quasi-Fermi level splitting (QFLS)

$$\mathrm{QFLS} = \mathrm{q}V_{\mathrm{OC,rad}} + \mathrm{kT}\ln(\mathrm{PLQY}) \quad (1)$$

Where k_B, T, PLQY, and $V_{OC,\mathrm{rad}}$ are Boltzmann constant, temperature, photoluminescence quantum yield and the radiative limit of the open-circuit voltage, respectively.

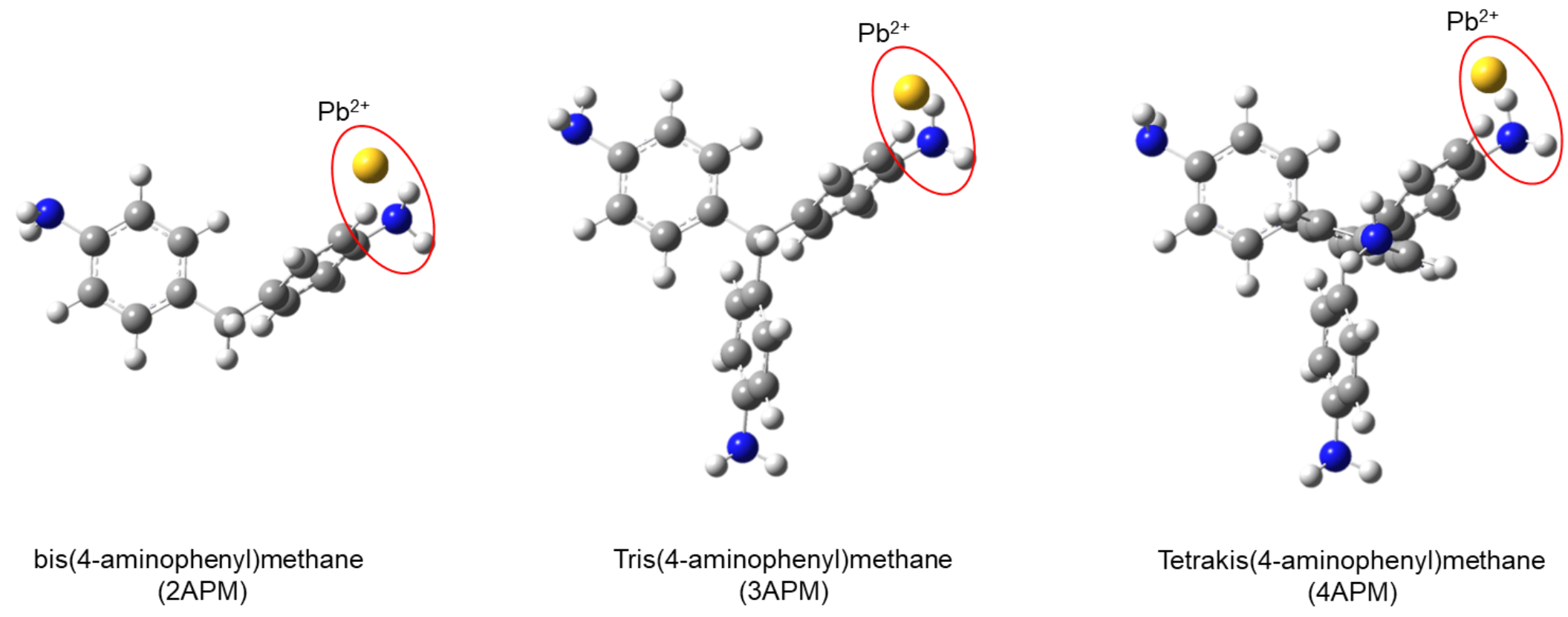

Supplementary Fig. 1. Molecular structures of additives. (a) 2APM, (b) 3APM and (c) 4APM.

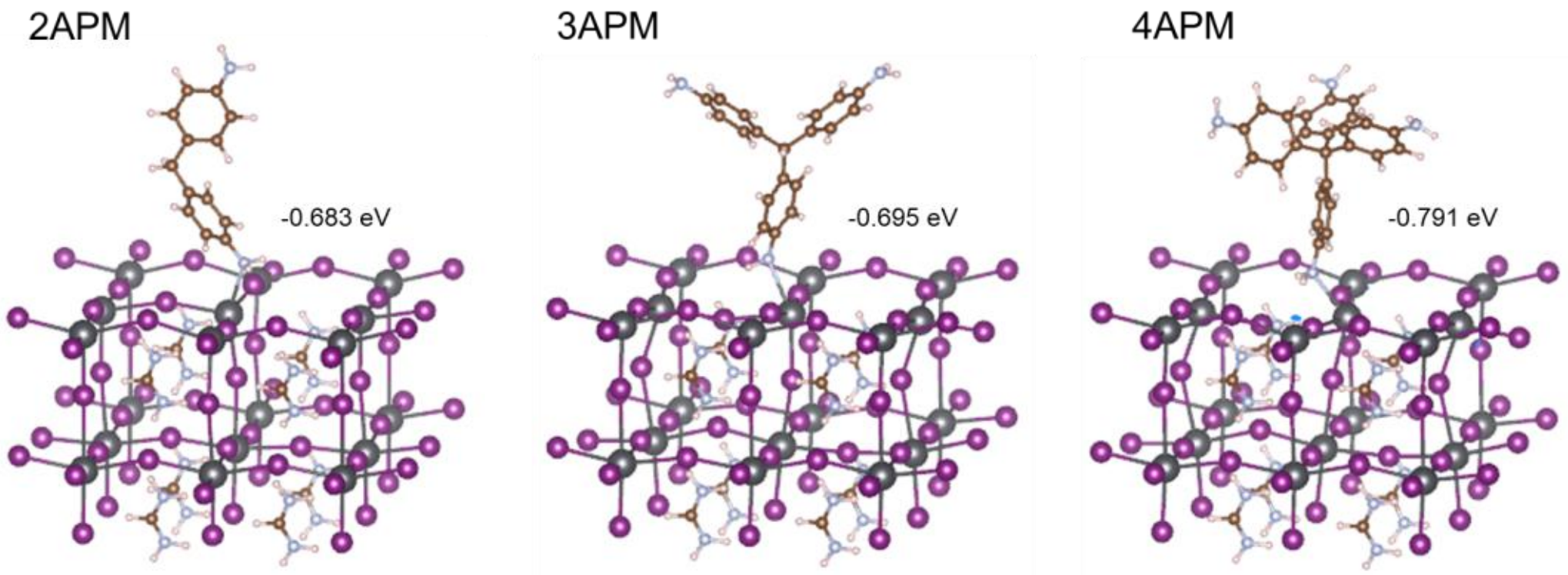

Supplementary Fig. 2. DFT calculations of the interaction energy (E_{int}). (a) 2APM, (b) 3APM, and (c) 4APM with V_I defect sites on the surface of perovskite.

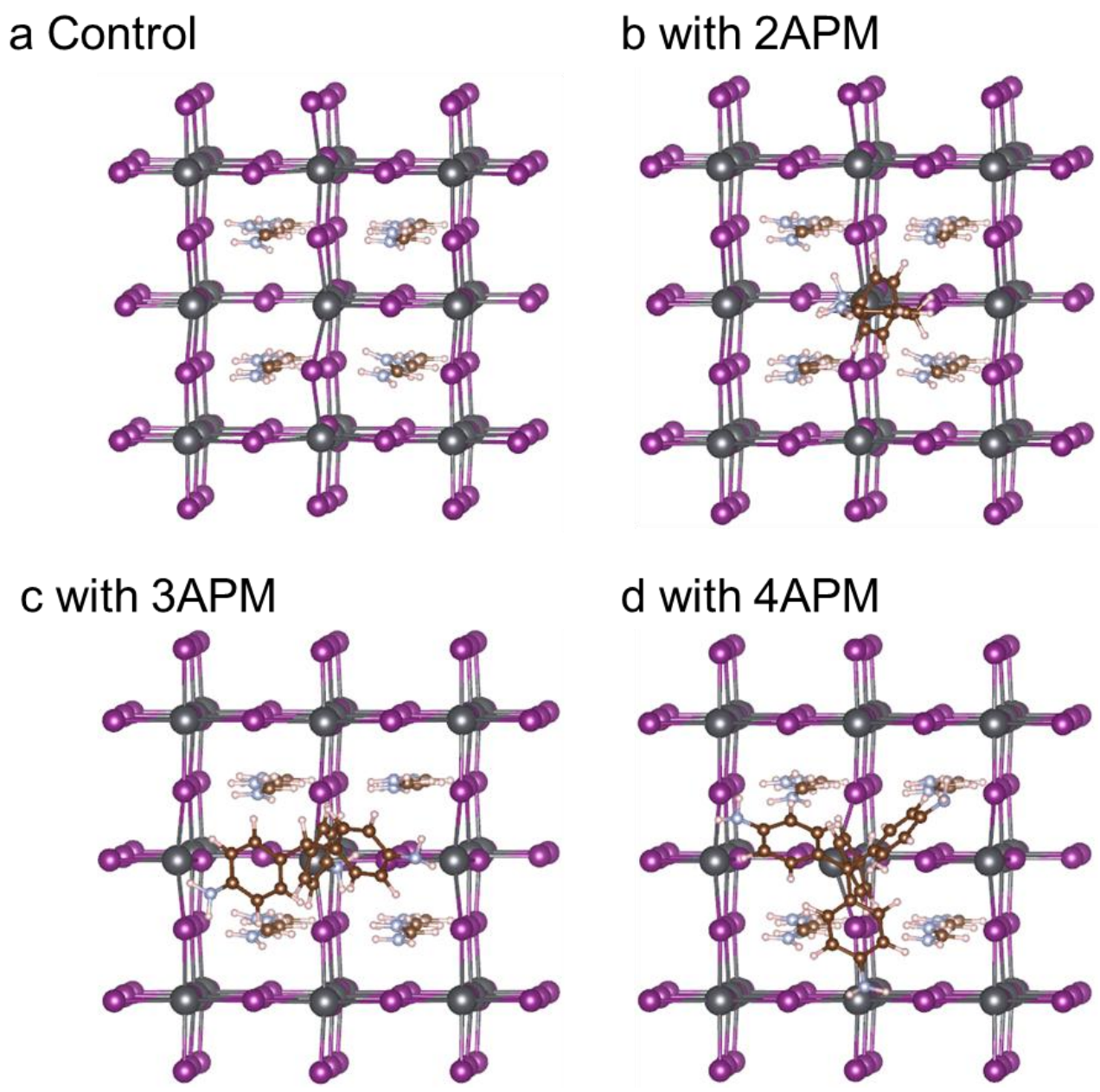

Supplementary Fig. 3. DFT calculations of the formation energies of V_I defect sites within perovskite. (a) control and with (b) 2APM, (c) 3APM and (d) 4APM.

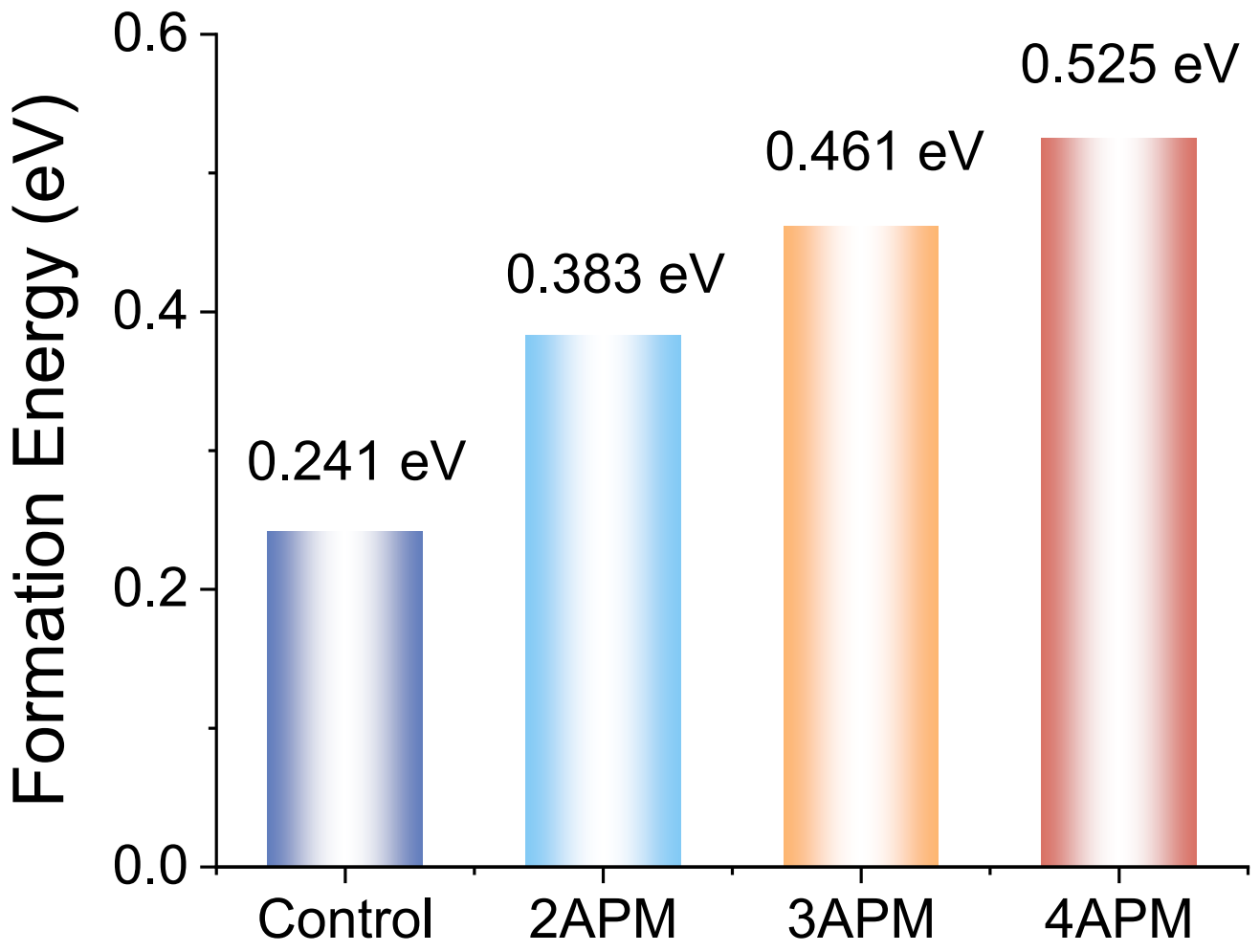

Supplementary Fig. 4. Formation energies values of V_I defect sites within perovskite films with different additives.

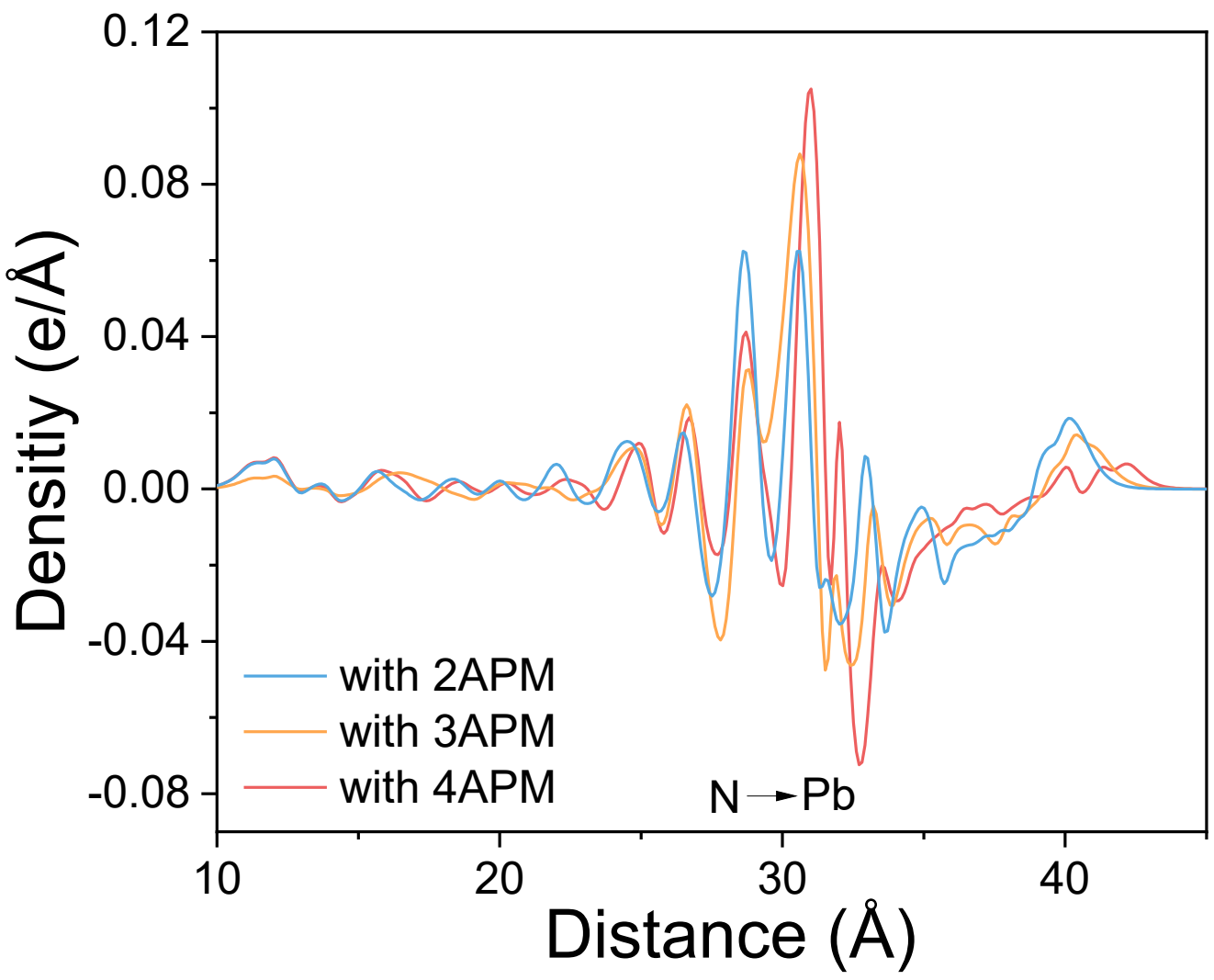

Supplementary Fig. 5. Longitudinal integration image of differential charge density. The isosurface values for the charge density difference plots is set at 0.0004 e/$Å^3$. Compared to 2APM and 3APM, 4APM exhibits more pronounced electron accumulation (positive) and electron depletion (negative), indicating more intense charge transfer and redistribution at the 4APM/perovskite interface. This reflects stronger chemical interactions between the 4APM molecules and surface Pb^{2+} atoms, which in turn facilitate more effective defect passivation and a more favorable interfacial energy level alignment.

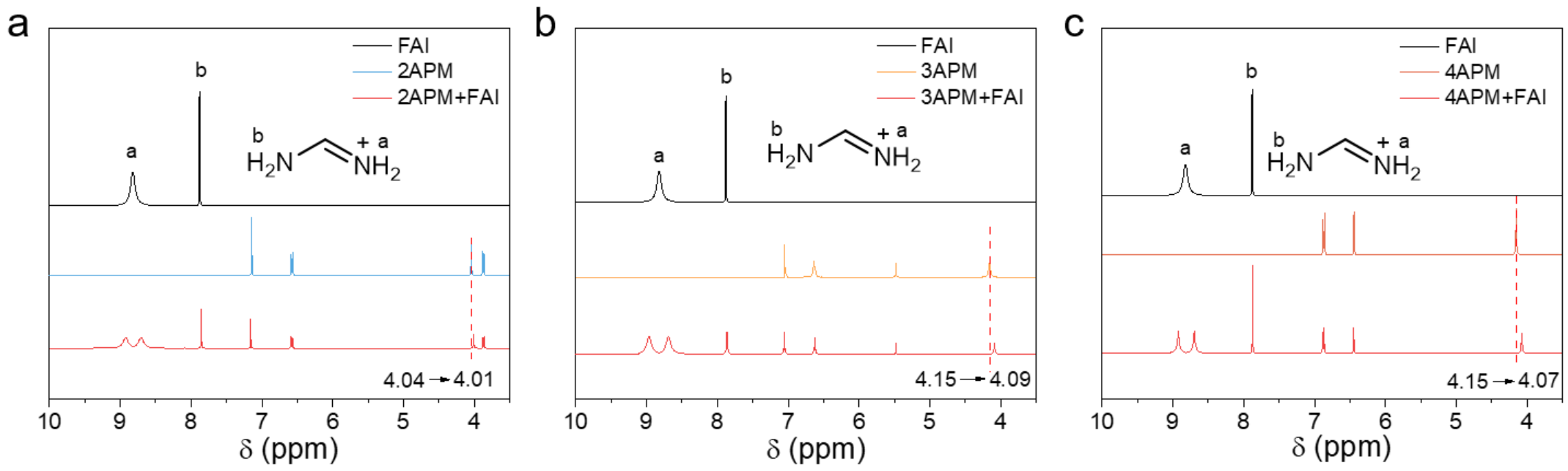

Supplementary Fig. 6. ^{1}H NMR spectra of additives with FAI. (a) 2APM-FAI, (b) 3APM-FAI and (c) 4APM-FAI.

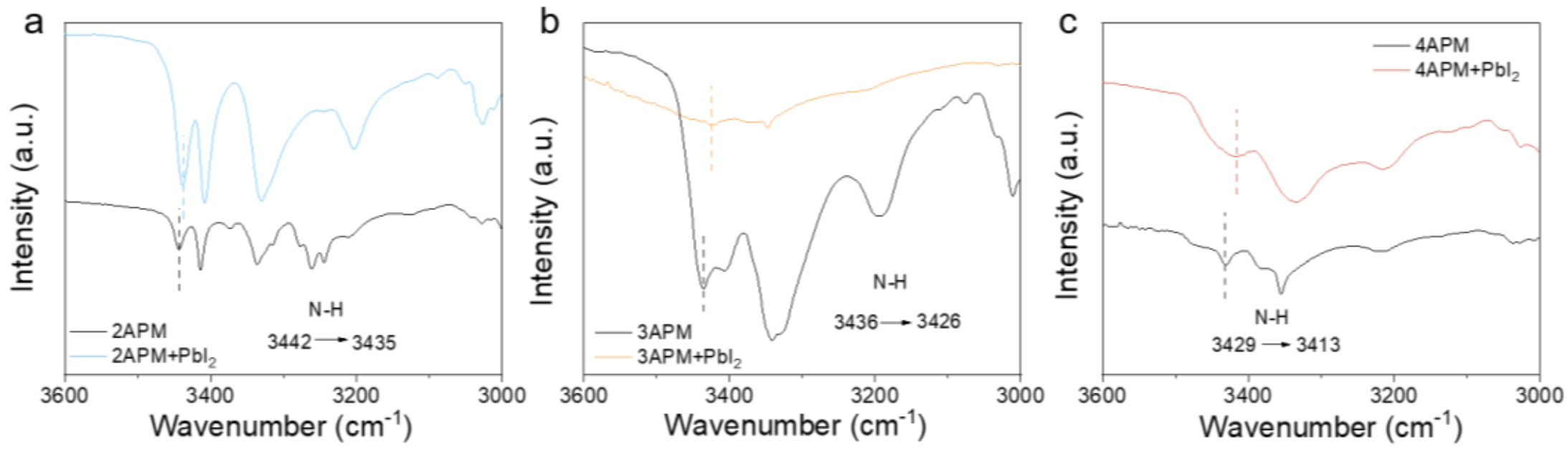

Supplementary Fig. 7. Fourier-transform infrared spectroscopy (FTIR) spectra. (a) pure 2APM and 2APM-PbI_2 films, (b) pure 3APM and 3APM-PbI_2 films, (c) pure 4APM and 4APM-PbI_2 films (the condition of a 1:1 molar ratio between NH_2 groups and Pb^{2+}). Dashed lines indicate the peak positions of N-H bond vibration signals.

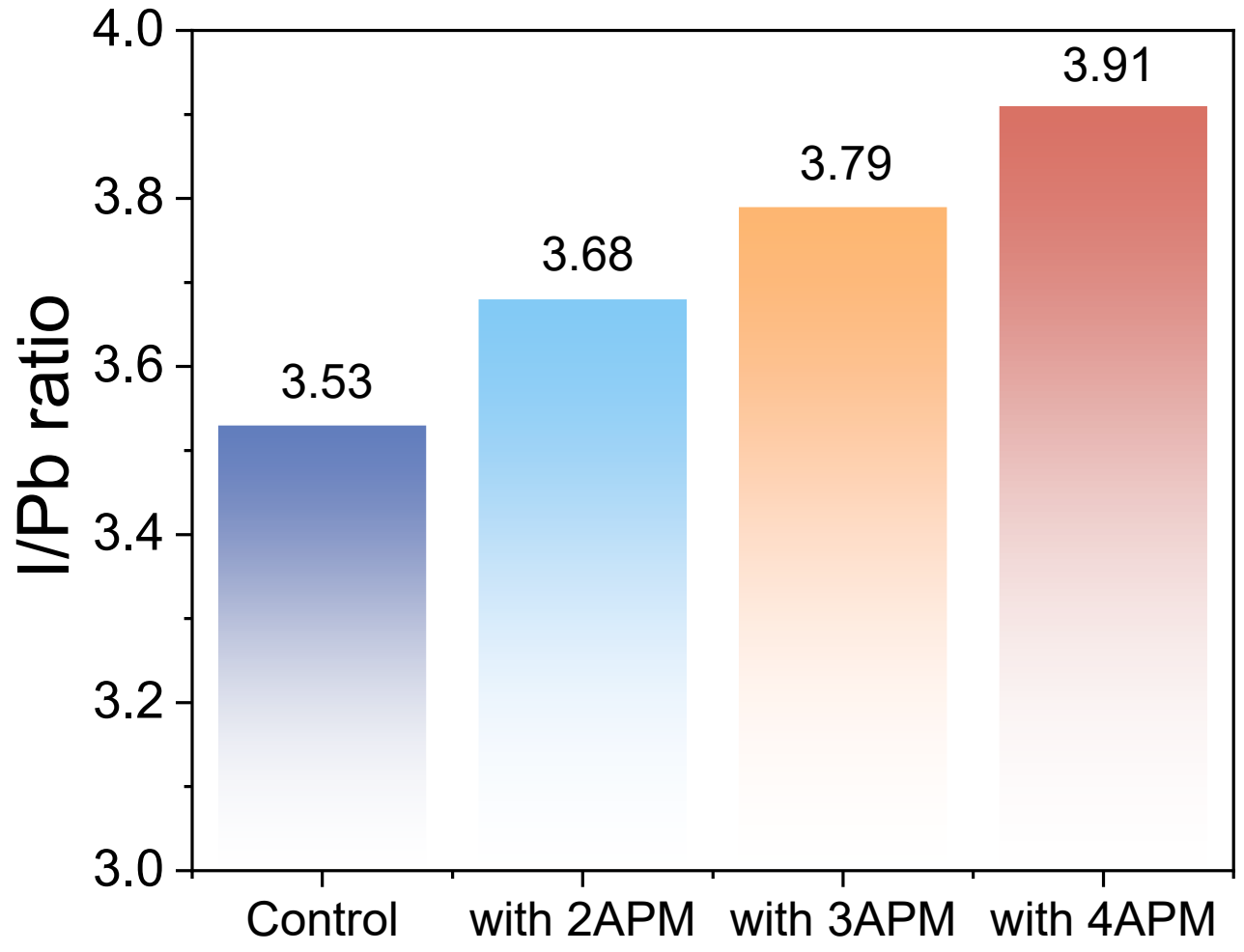

Supplementary Fig. 8. I/Pb ratios extracted from Fig. 2g.

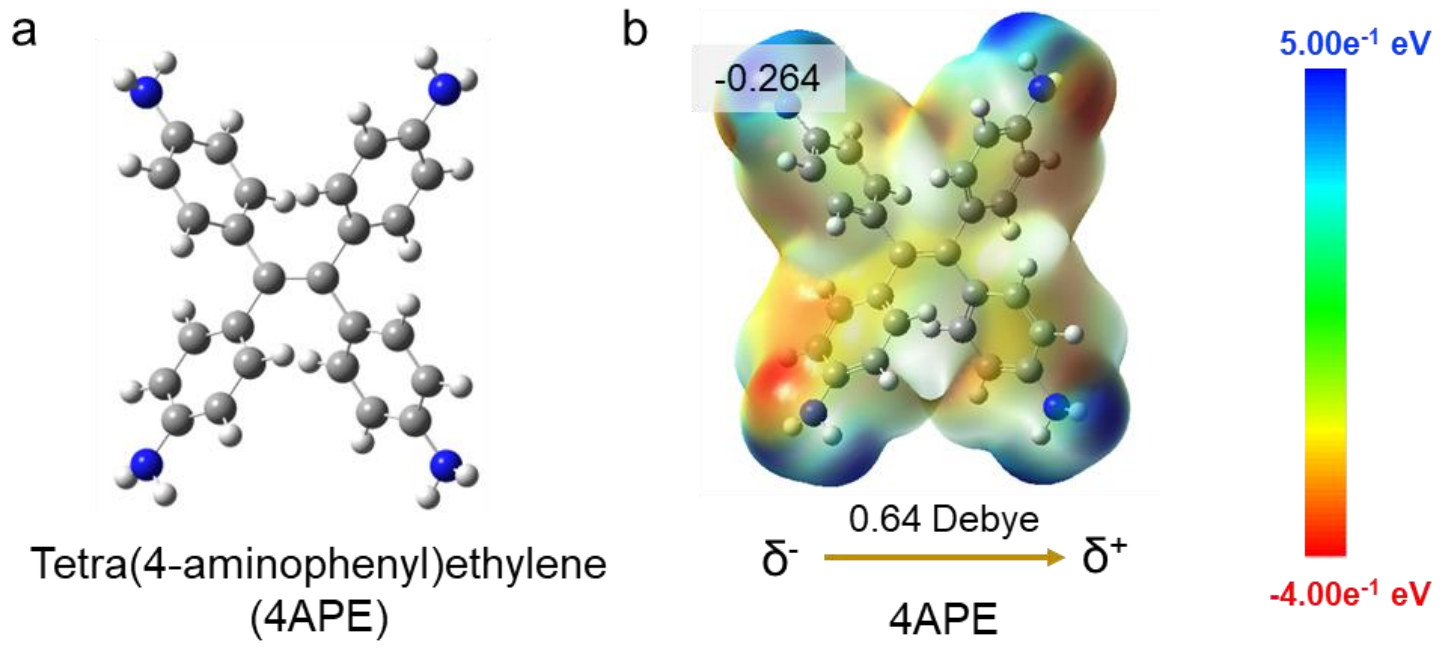

Supplementary Fig. 9. Properties of 4APE molecule. (a) Molecular structure of 4APE. (b) Calculated ESP profiles for 4APE molecule.

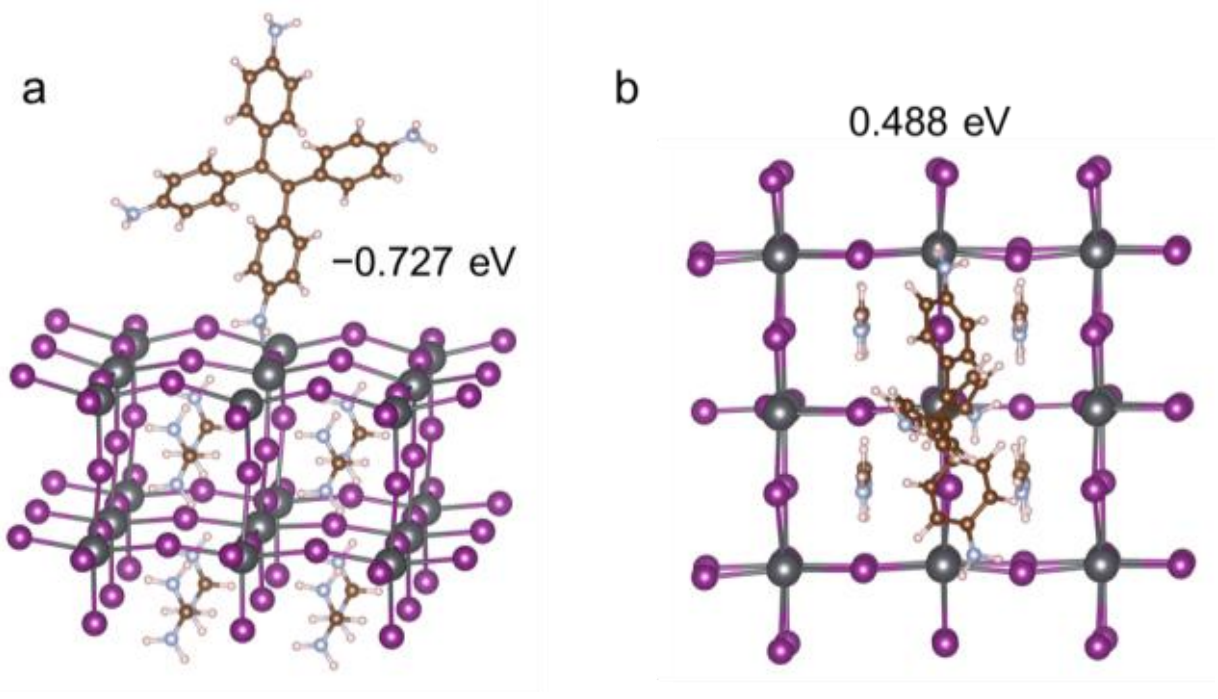

Supplementary Fig. 10. Simulation of interactions between 4APE and perovskite defects. (a) DFT calculations of the interaction energy (Eint) of 4APE with V_I defect sites on the surface of perovskite. (b) DFT calculations of the formation energies of V_I defect sites within perovskite with 4APE.

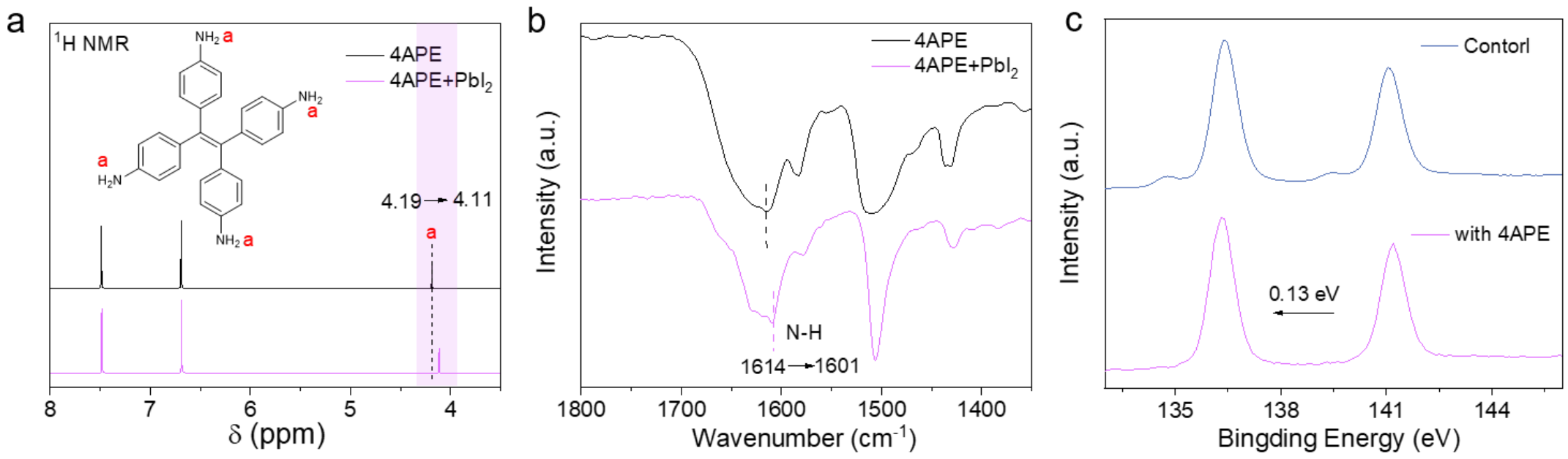

Supplementary Fig. 11. Interactions between 4APE and PbI_2. (a) ^1H NMR spectra of 4APE with and without PbI_2. (b) FTIR spectra of 4APE and PbI_2-4APE films. (c) Pb 4*f* XPS spectra of perovskite films without and with 4APE treatment.

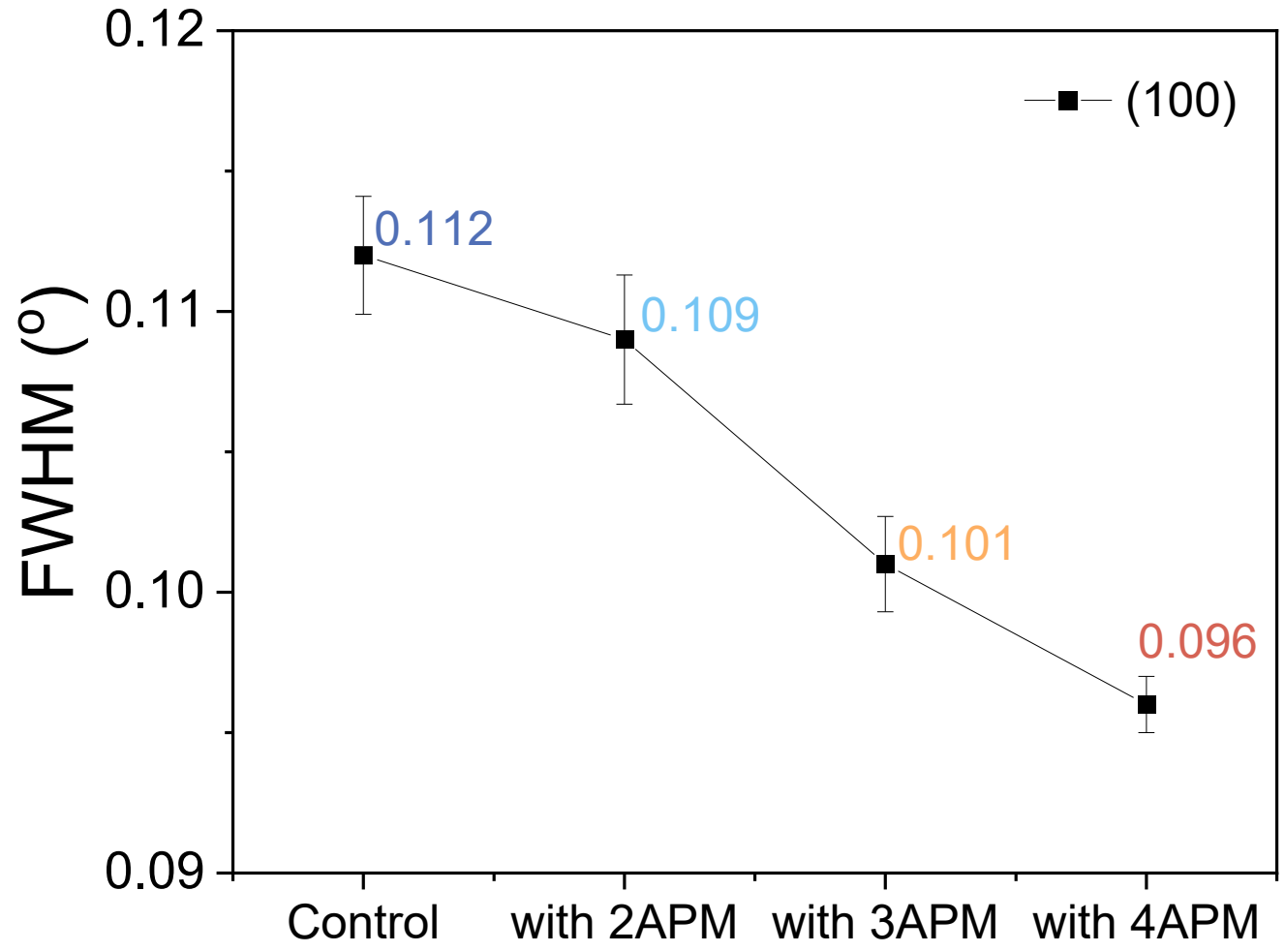

Supplementary Fig. 12. The full width at half maximum (FWHM) of the (100) diffraction peak. (among 3 perovskite films for each condition). Error bars represent the standard deviation.

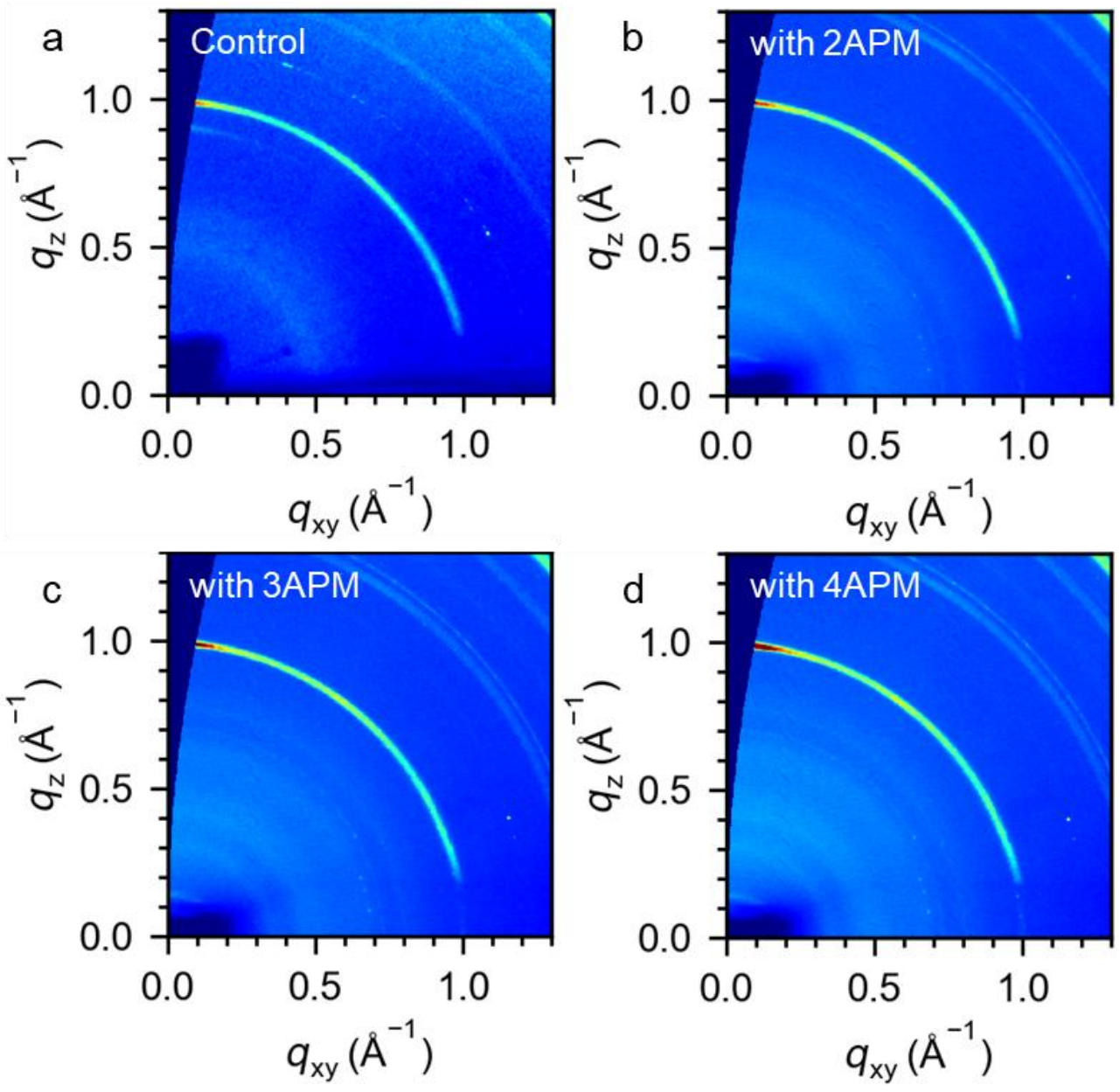

Supplementary Fig. 13. GWIAXS patterns of perovskite films. (a) control and with (b) 2APM, (c)3APM and (d) 4APM passivation.

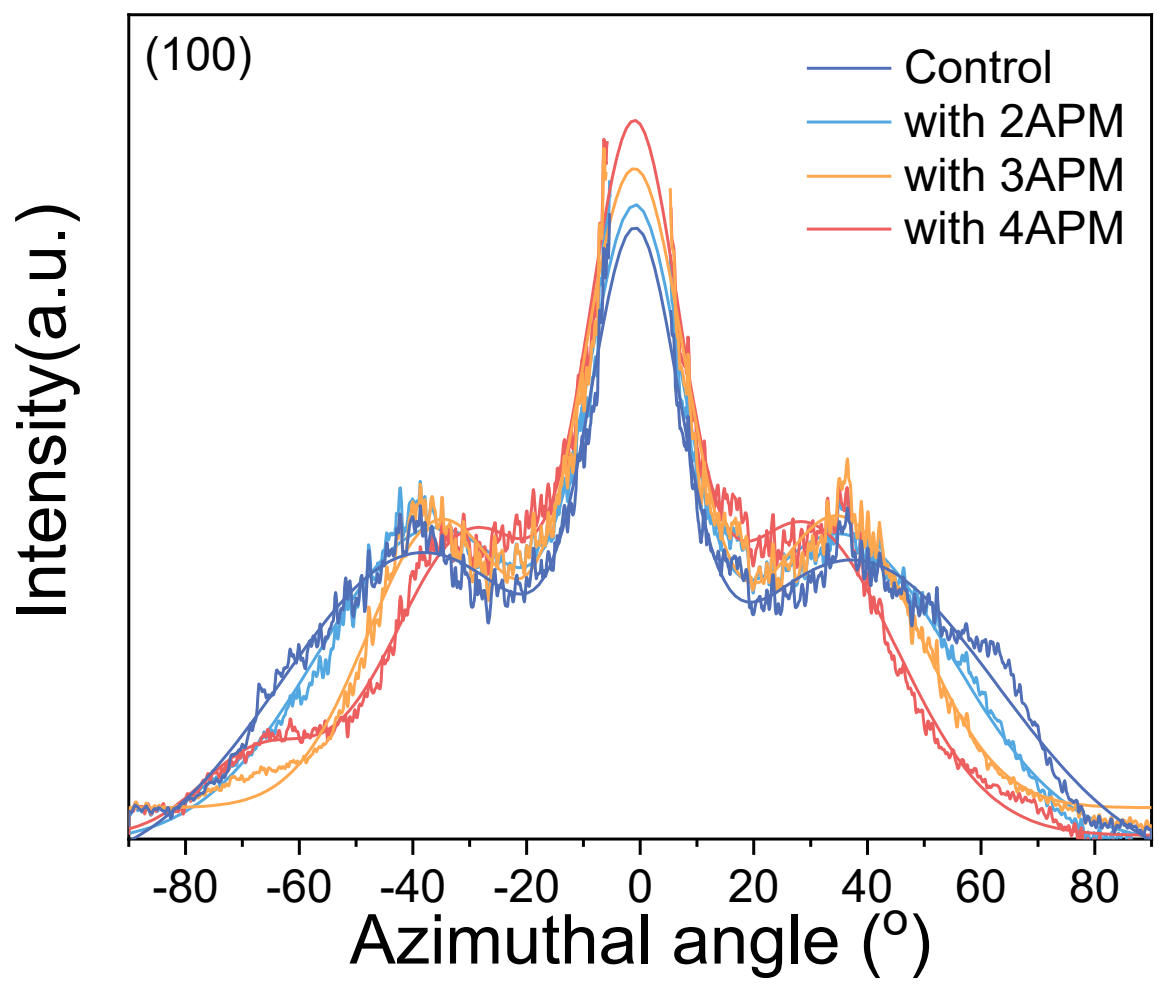

Supplementary Fig. 14. The plots of perovskite film of (100) facet along the ring at the range about 0.9-1.05 $Å^{-1}$.

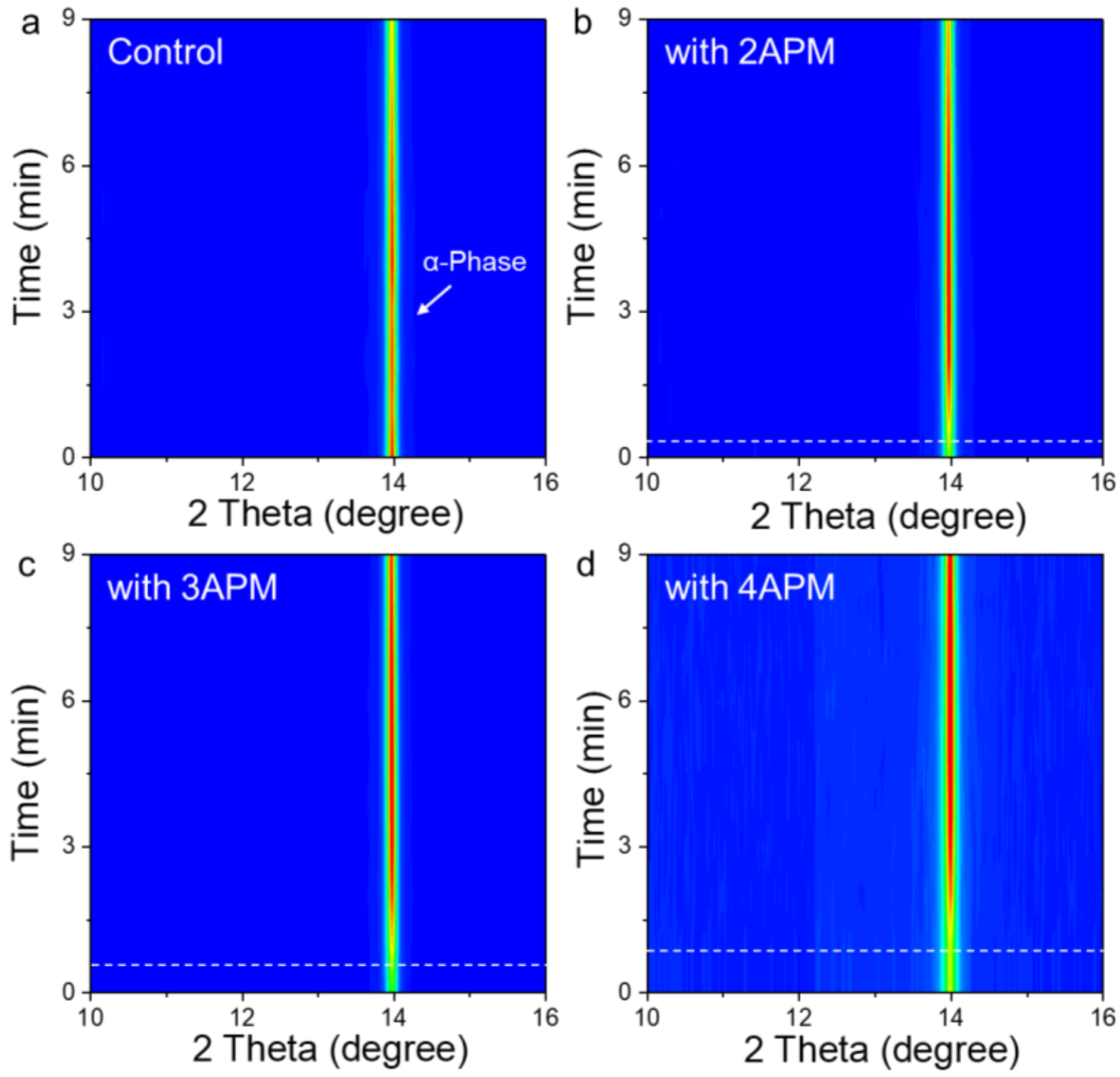

Supplementary Fig. 15. In situ XRD patterns of the crystallization process of perovskite films. (a) control and (b) with 2APM, (c) 3APM and (d) 4APM over time.

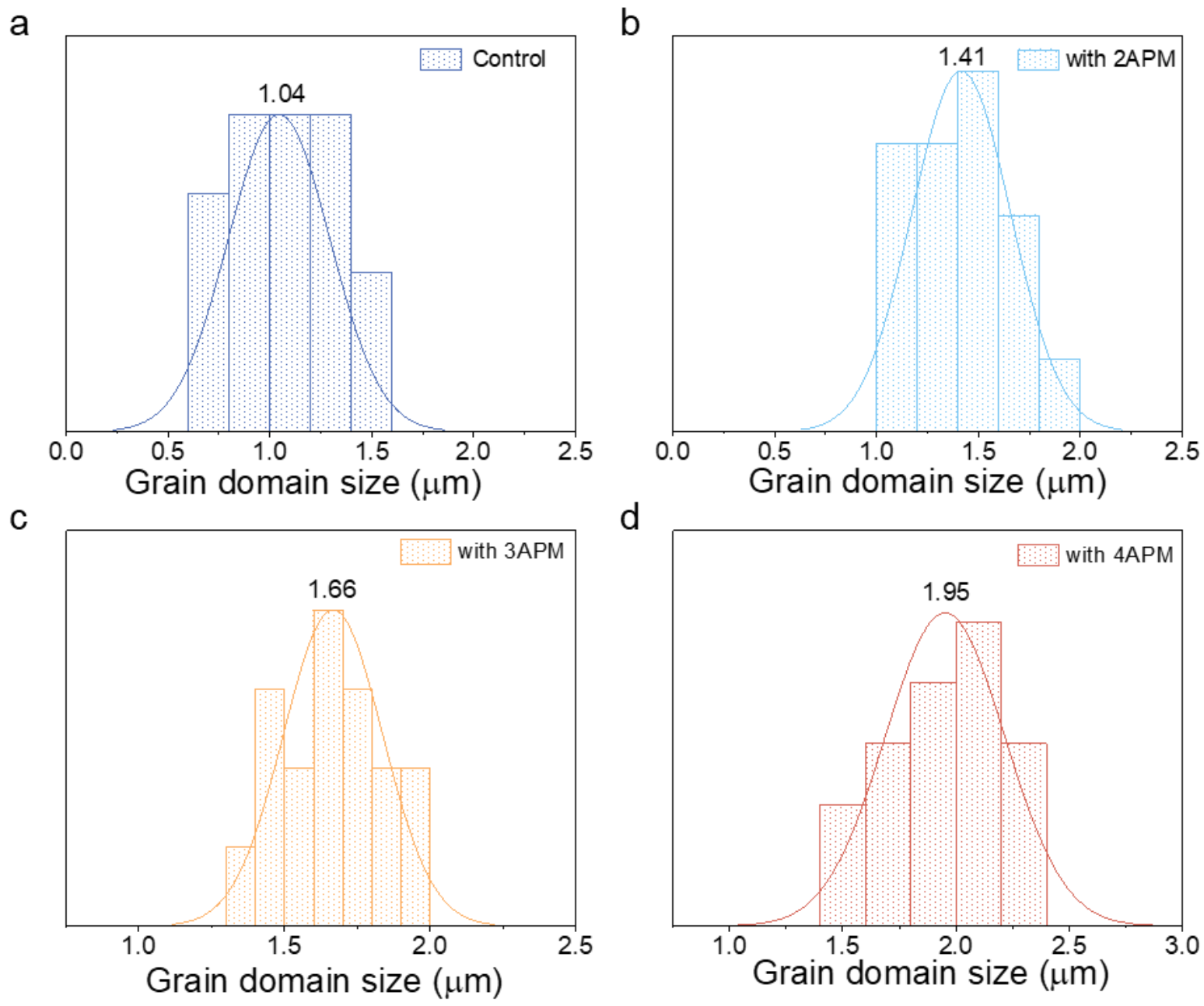

Supplementary Fig. 16. Domain size distribution of perovskite films. (a) control and (b) with 2APM, (c) 3APM and (d) 4APM. (Count: 30 grains)

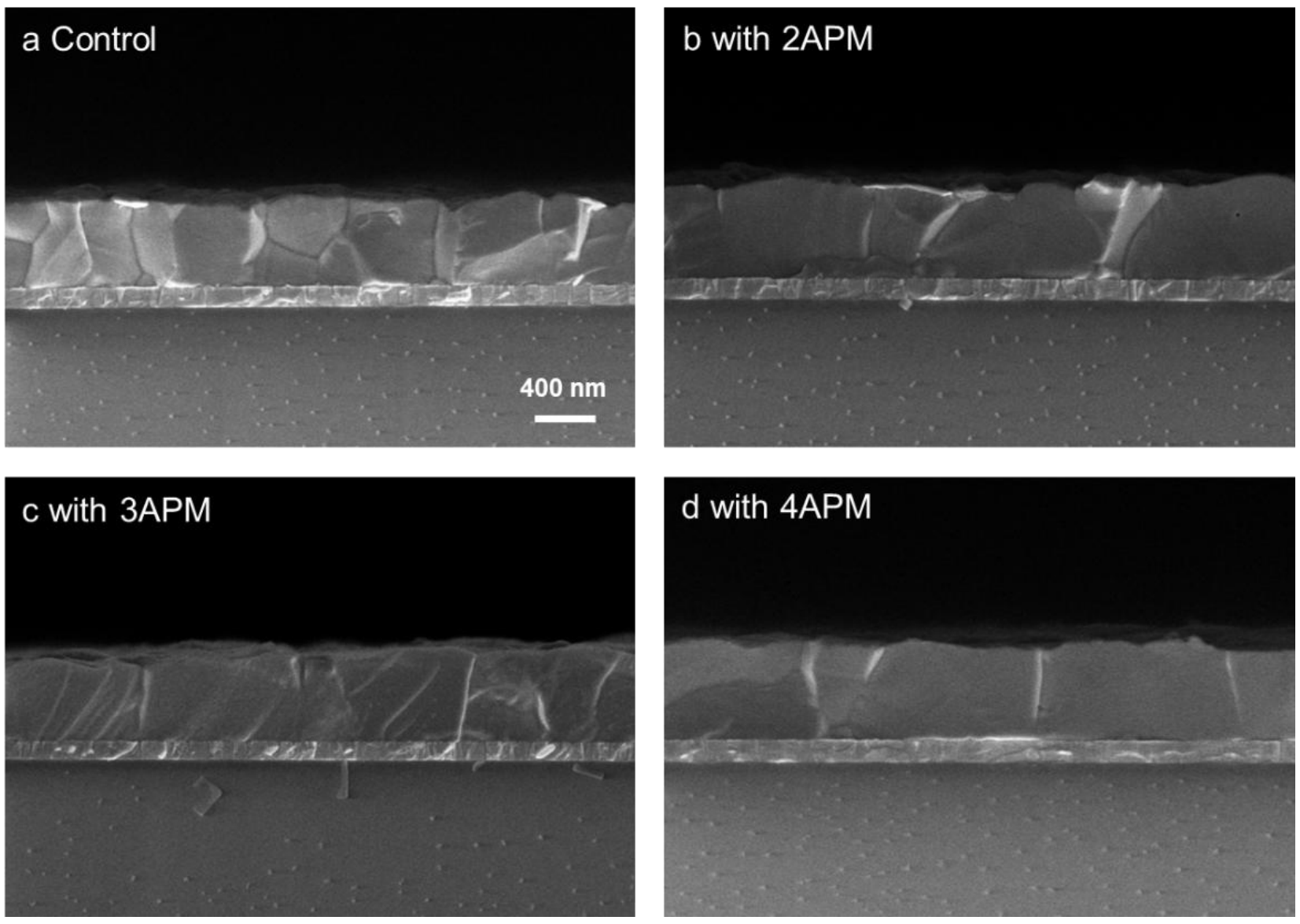

Supplementary Fig. 17. Cross-sectional SEM images of perovskite films. (a) control and (b) with 2APM, (c) 3APM and (d) 4APM, respectively.

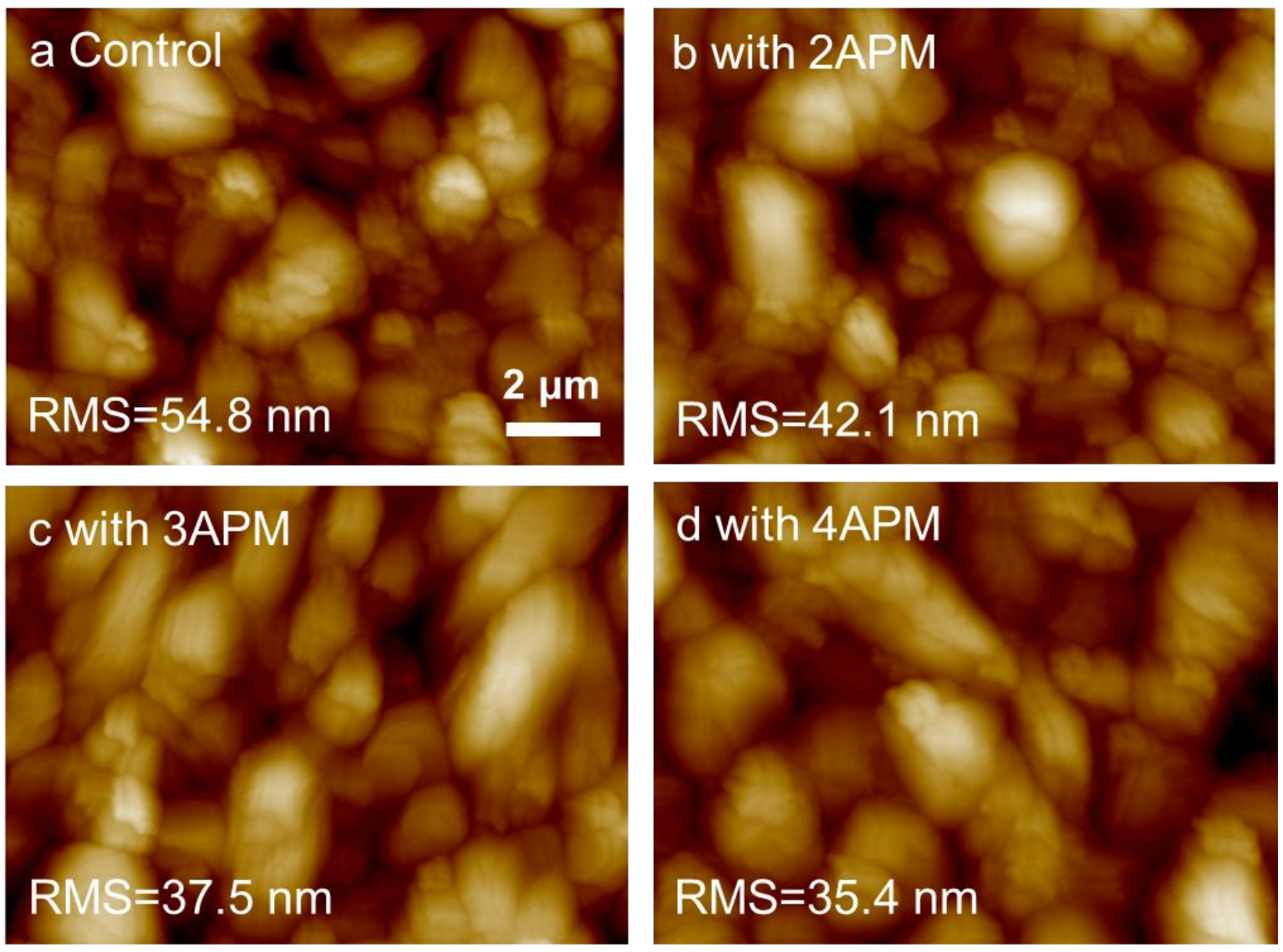

Supplementary Fig. 18. Topography AFM images of perovskite films. (a) control and (b) with 2APM, (c) 3APM and (d) 4APM.

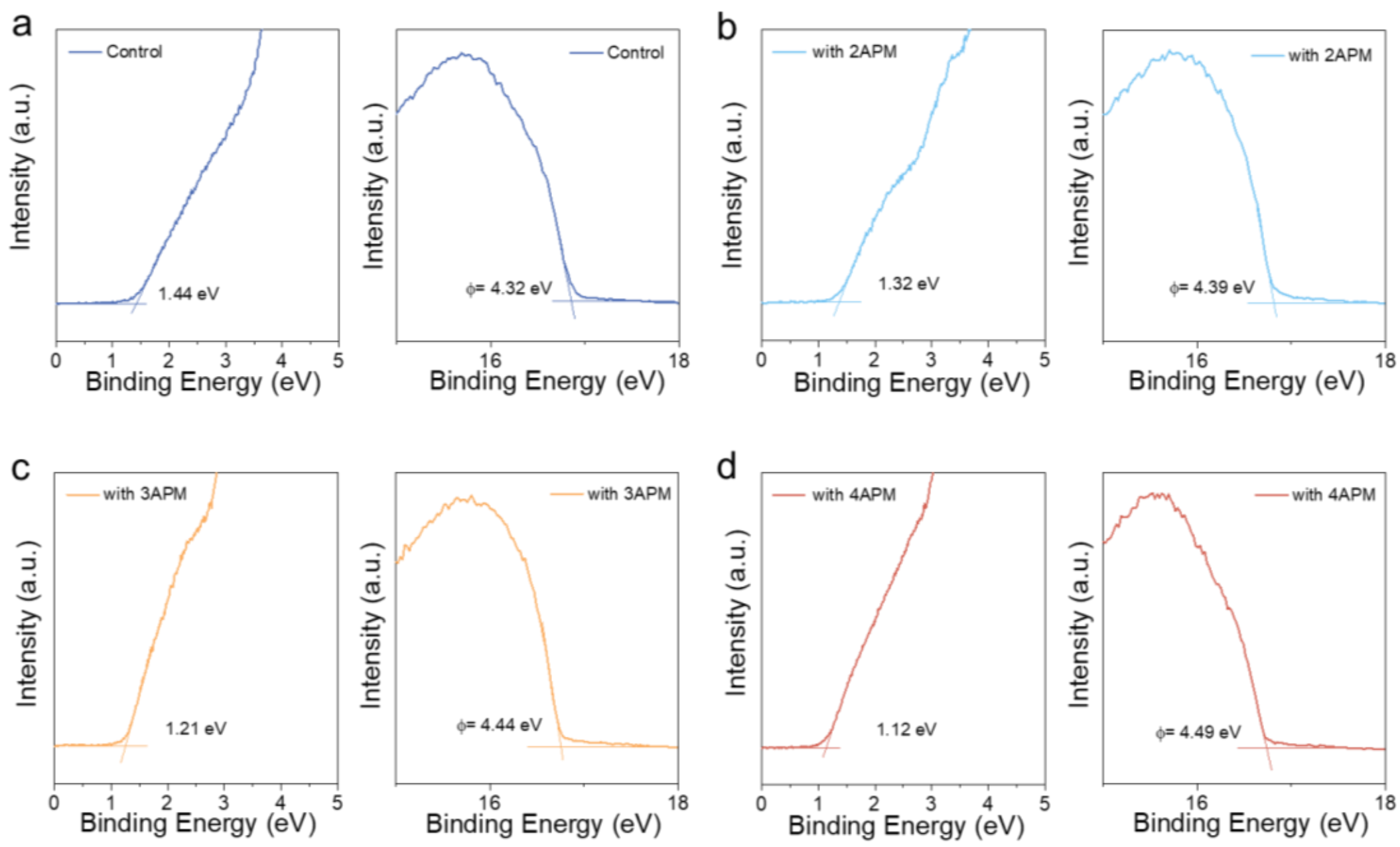

Supplementary Fig. 19. UPS data of perovskite films. (a) control and (b) with 2APM, (c) 3APM and (d) 4APM.

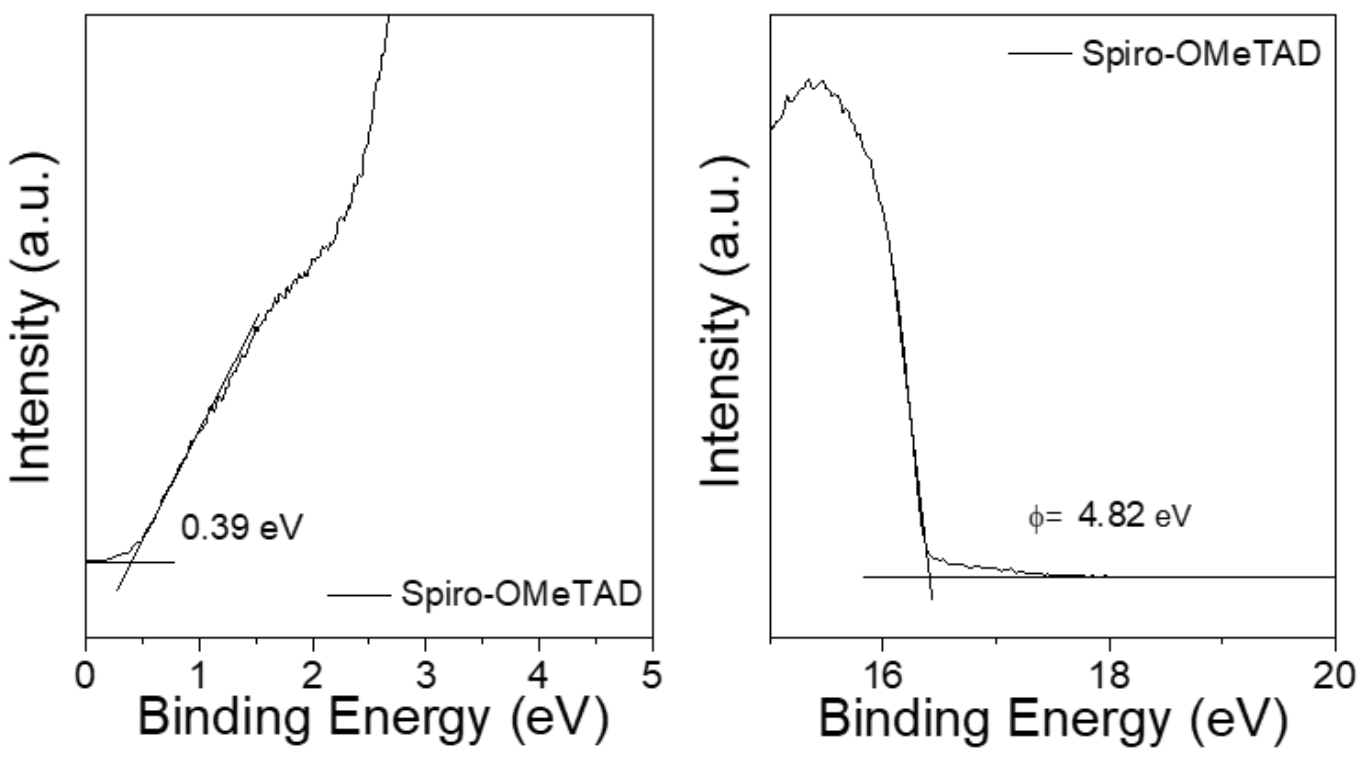

Supplementary Fig. 20. UPS data of Spiro-OMeTAD.

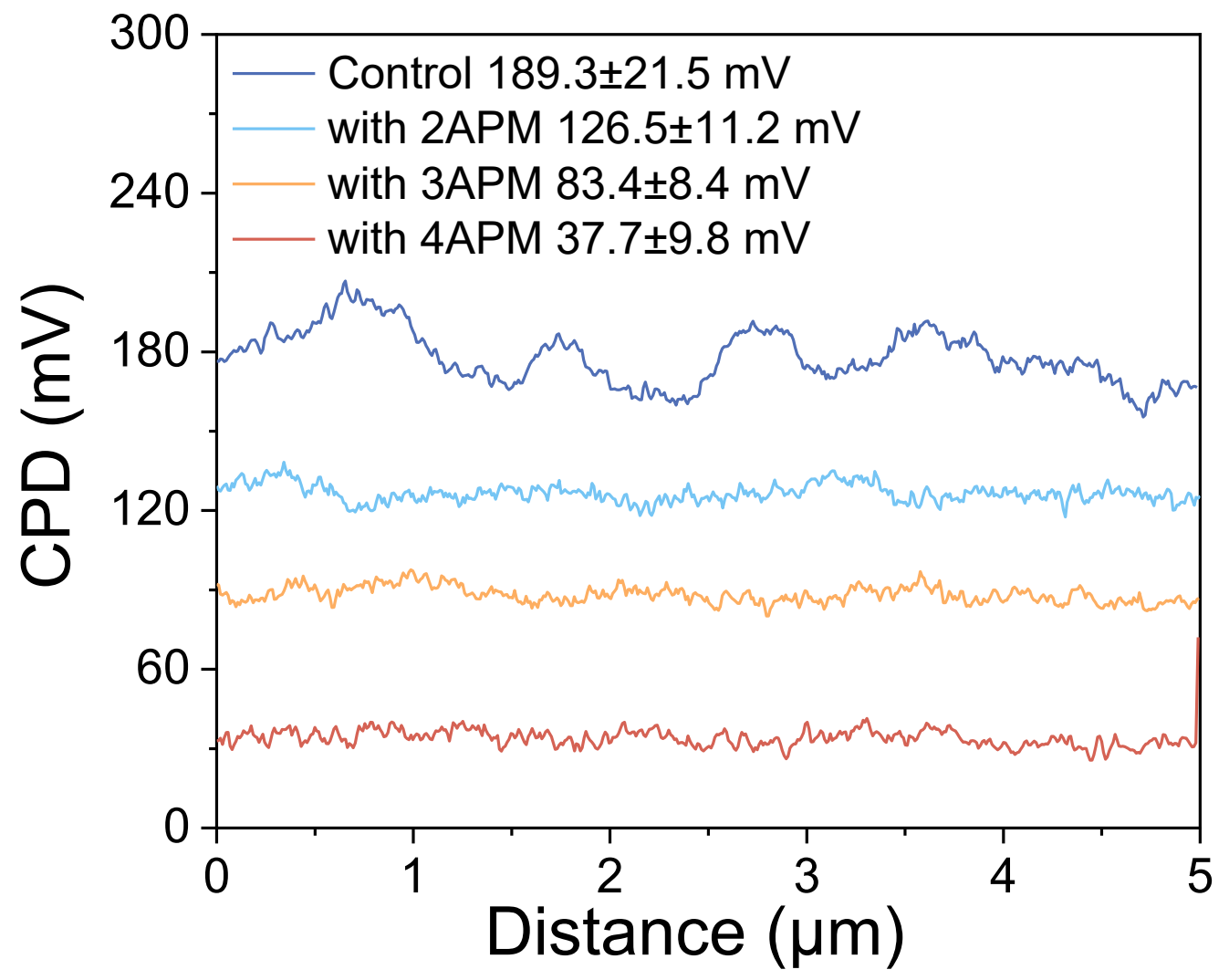

Supplementary Fig. 21. CPD variation of perovskite films without and with 2APM, 3APM and 4APM along the line scan in KPFM images.

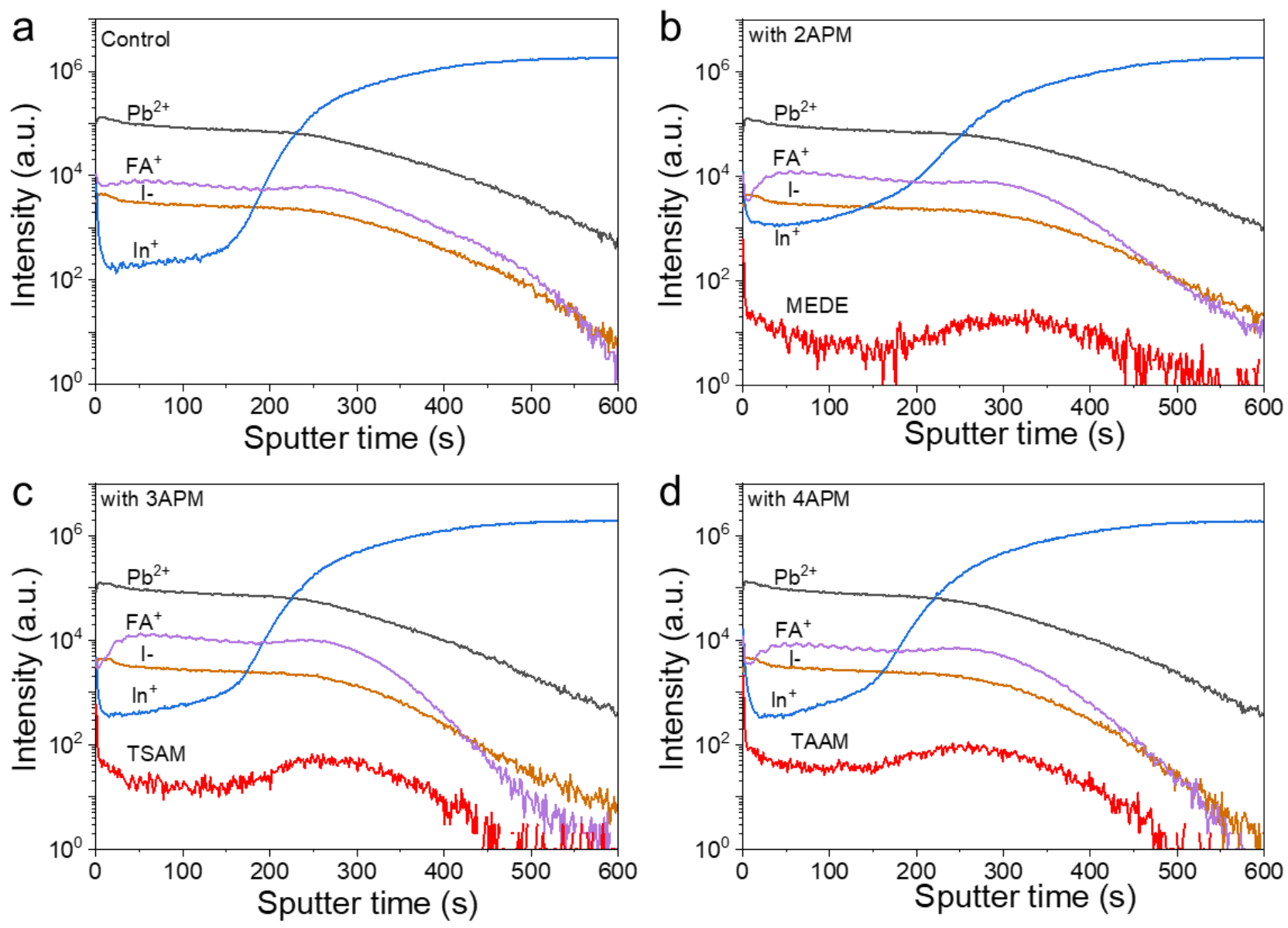

Supplementary Fig. 22. TOF-SIMS depth profiles of perovskite films. (a) control and (b) with 2APM, (c) 3APM and (d) 4APM deposited on silicon substrates.

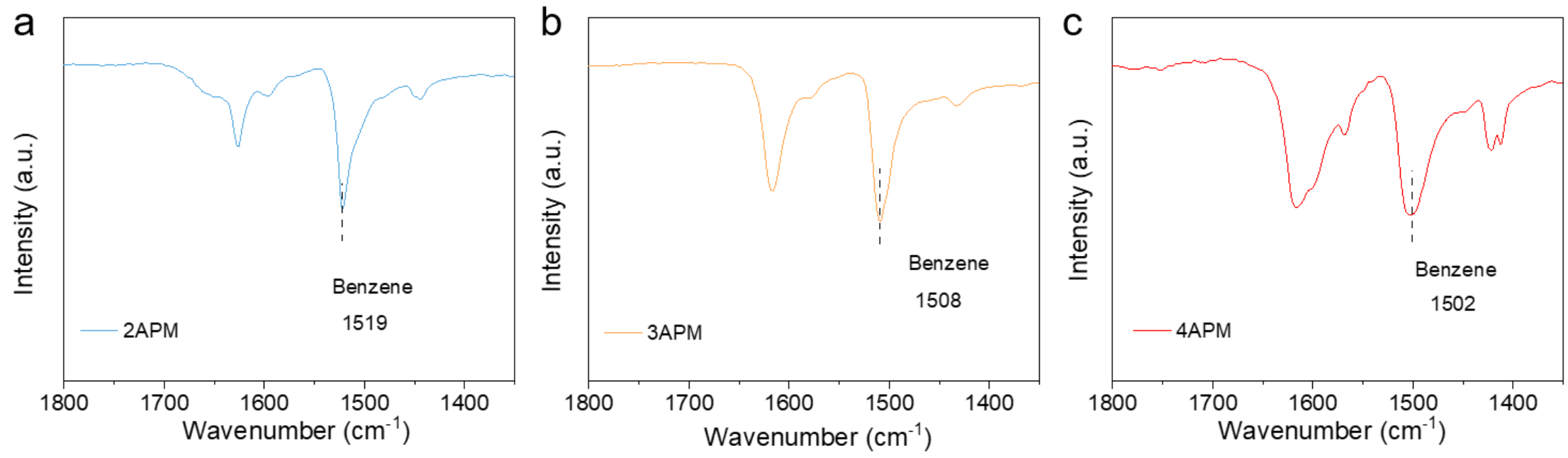

Supplementary Fig. 23. FTIR spectra of perovskite films. (a) with 2APM, (b) with 3APM and (c) with 4APM. Dashed lines indicate the peak positions of benzene bond vibration signals.

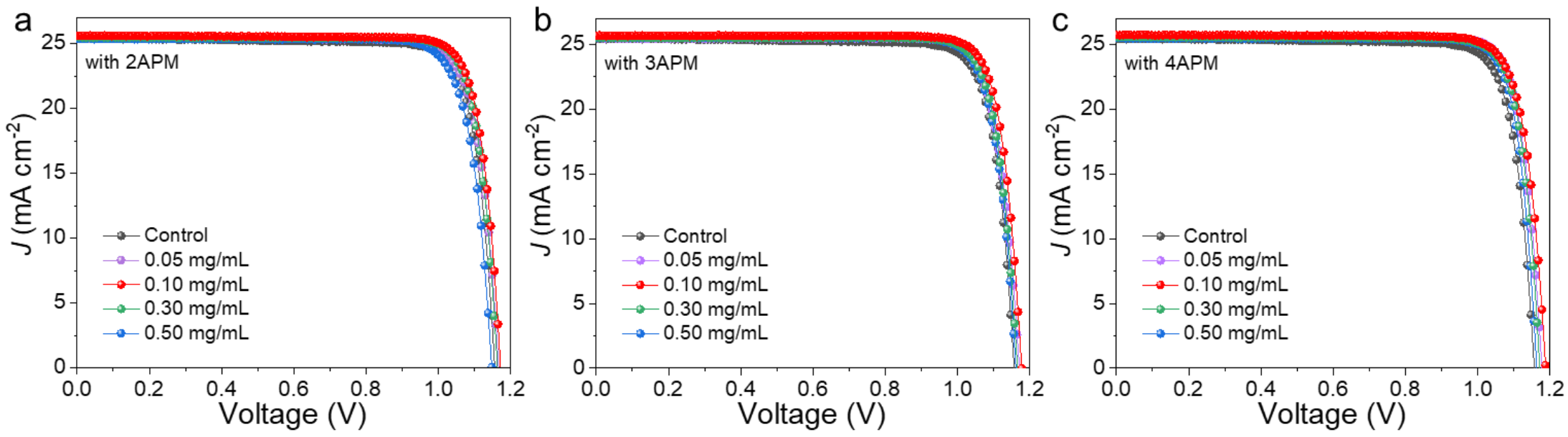

Supplementary Fig. 24. *J-V* curves of perovskite solar cells incorporating different concentrations of additives. (a) with 2APM, (b) with 3APM and (c) with 4APM, corresponding to the photovoltaic parameters summarized in Supplementary Table 3-5. (The device with 0.1 mg/mL phenylamine-based molecules exhibited the highest PCE among all concentrations.)

At lower concentrations, an insufficient number of additive molecules is available to effectively passivate the abundant defect sites, particularly under-coordinated Pb^{2+} at film surfaces and grain boundaries, resulting in a higher density of non-radiative recombination centers and consequently suboptimal V_{OC} and FF. Conversely, at higher concentrations, the excessive additive molecules disrupt the chemical environment of the perovskite precursor solution and interfere with the crystallization process, compromising film quality, introducing unwanted organic impurity phases, leading to inferior device performance. The optimal concentration of 0.1 mg/mL therefore strikes the most favorable balance between sufficient defect passivation and minimal disruption to perovskite crystallization, affording the best overall device performance.

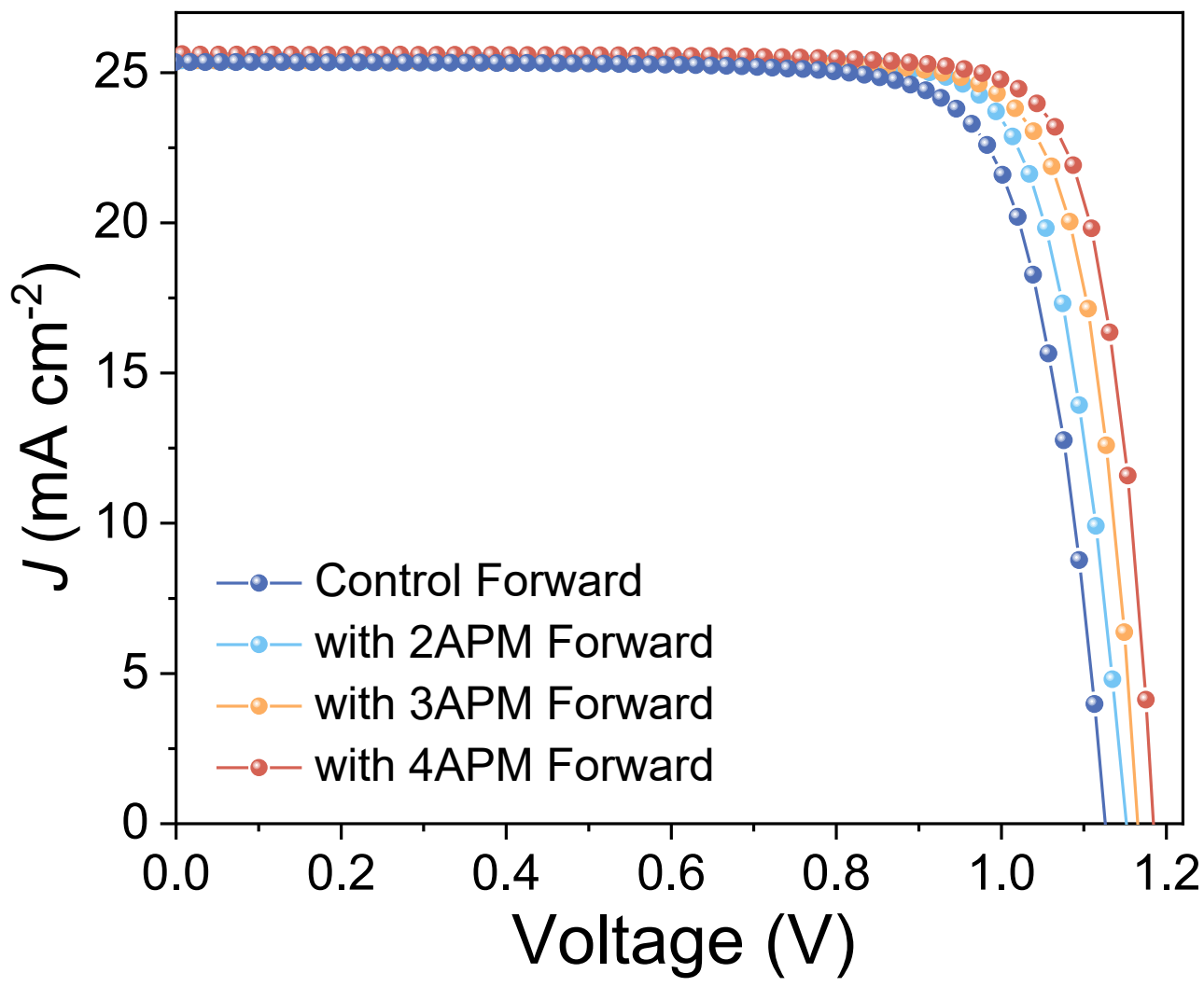

Supplementary Fig. 25. Forward *J-V* curves of the PSCs without and with 2APM, 3APM and 4APM.

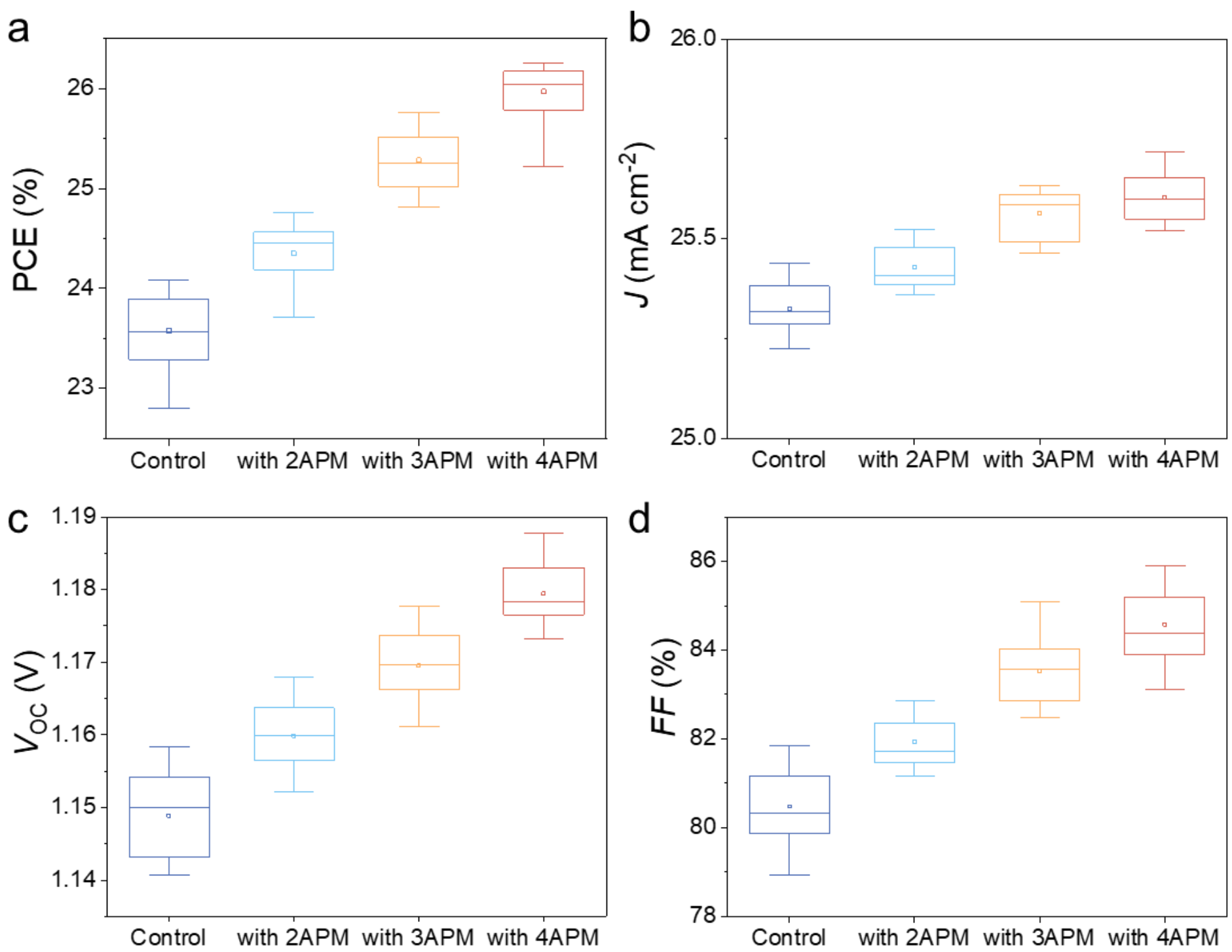

Supplementary Fig. 26. The statistical distribution of photovoltaic parameters. (a) PCE, (b) J_{SC}, (c) V_{OC} and (d) *FF* of PSCs from 20 devices for each condition. The central line of the box plot represents the median, the box length indicates data dispersion, while the whiskers denote the maximum and minimum values.

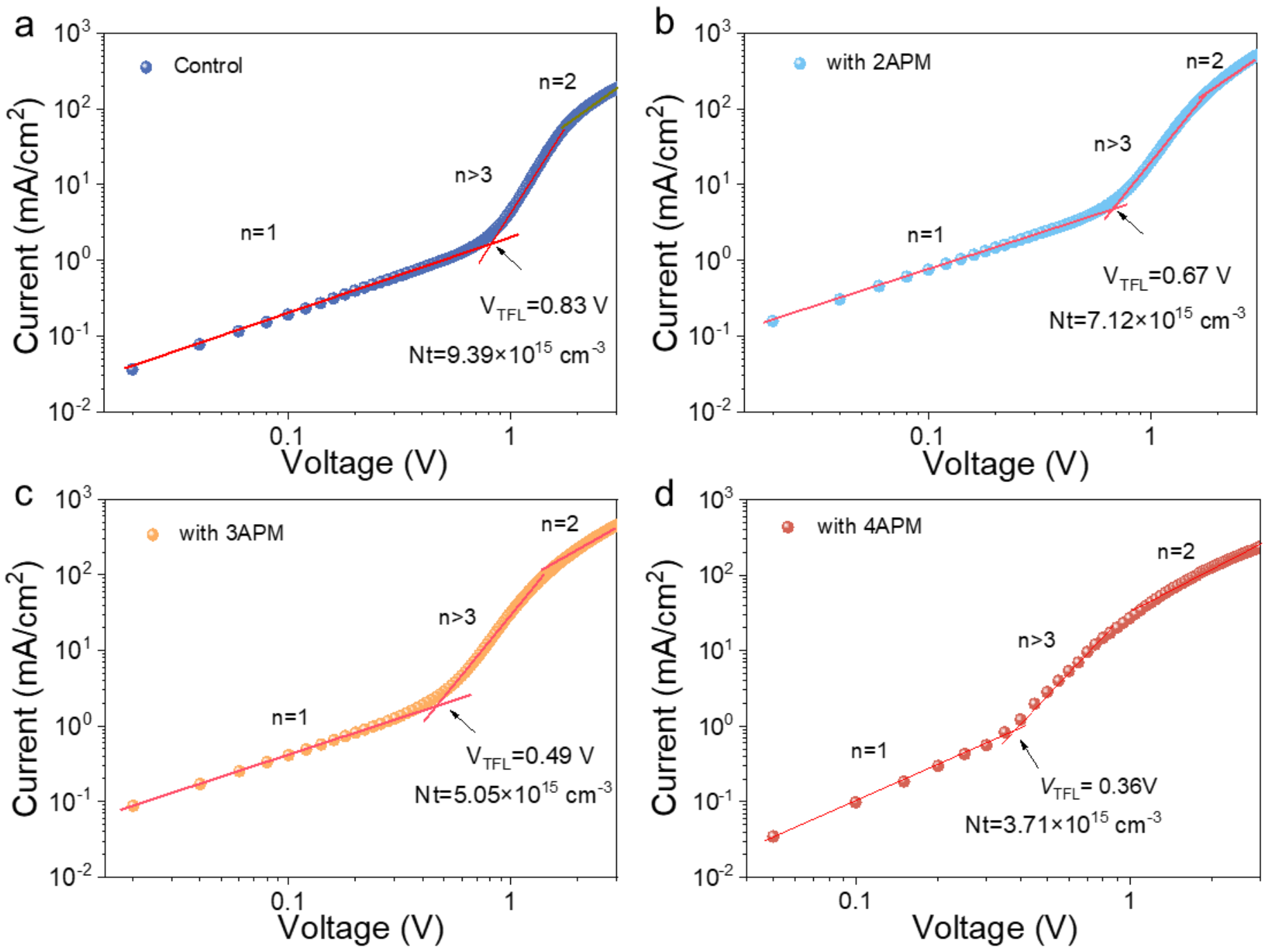

Supplementary Fig. 27. Space charge limited current (SCLC) curve of hole-only PSC devices. (a) control, (b) with 2APM, (c) with 3APM and (d) with 4APM. V_{TFL} values indicate trap density variation.

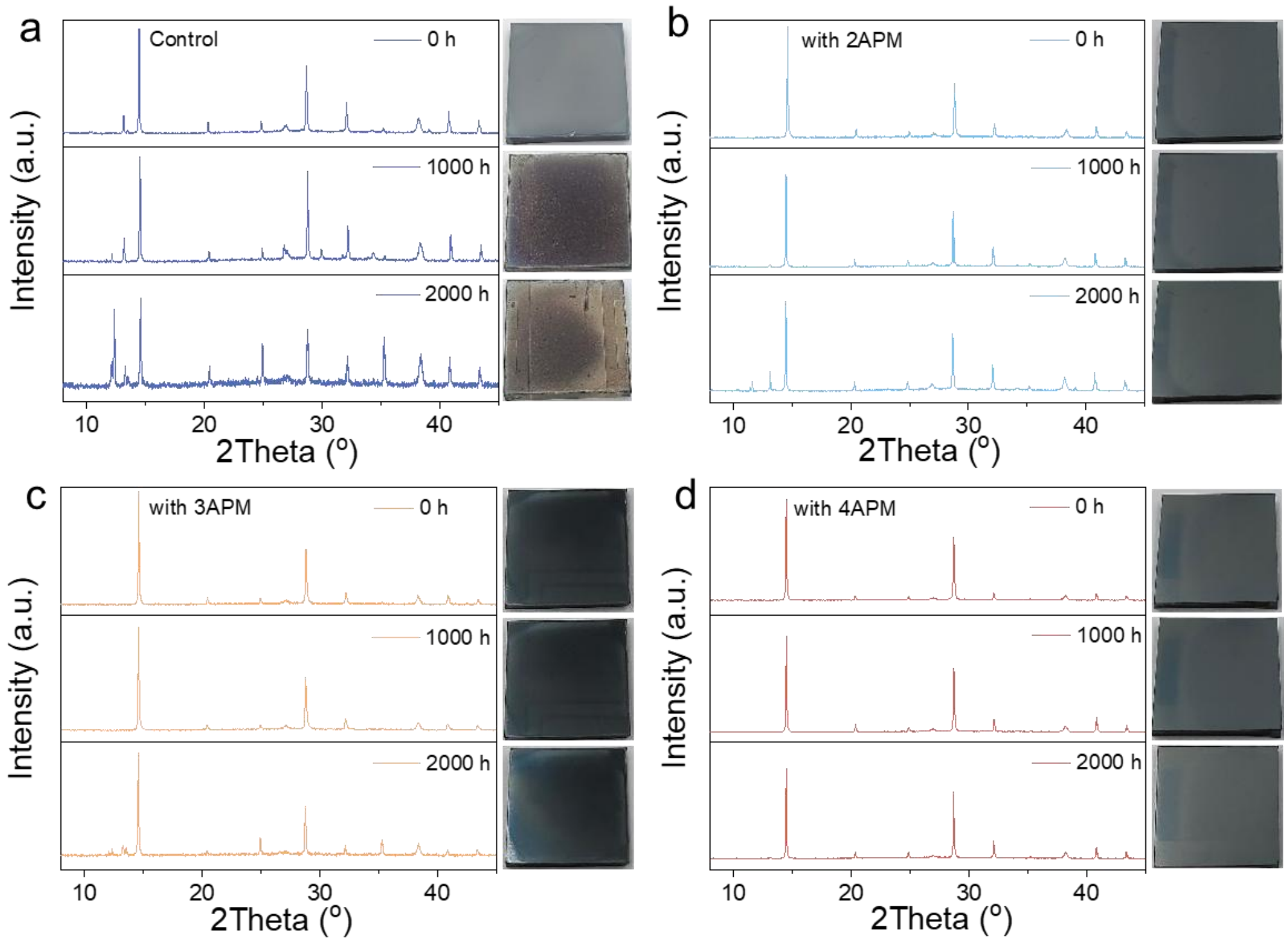

Supplementary Fig. 28. XRD measurements of aged perovskite films. (a) control, (b) 2APM, (c) 3APM and (d) 4APM-based perovskite films before and after aging for 2000 h in air environments. Inset: aging image of perovskite thin film.

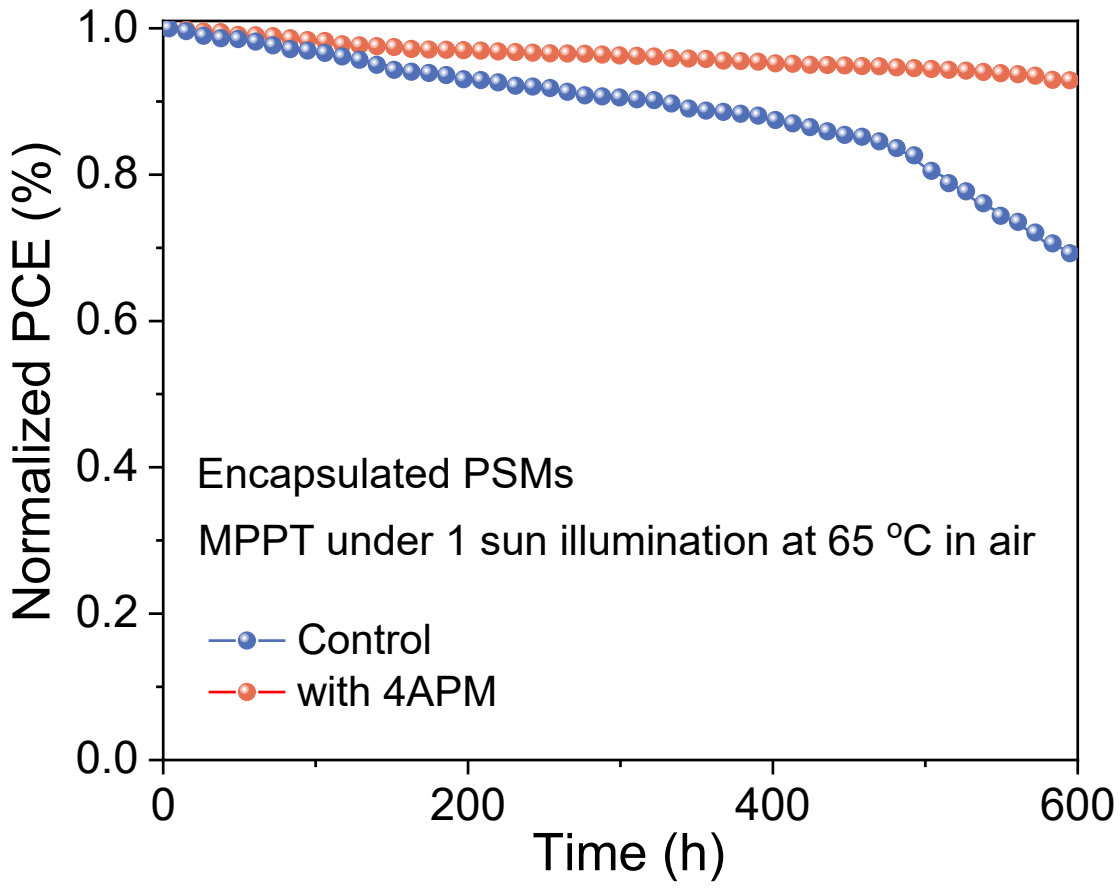

Supplementary Fig. 29. Operational stability of the encapsulated PSMs under 1-sun MPPT conditions at 65 °C in ambient air.

Supplementary Table 1. The interaction energies between molecules and perovskite surface defects. The interaction energies are defined as:

$$E_{int} = E_{defect+molecule} - E_{defect} - E_{molecule} \quad (2)$$

	2APM	3APM	4APM
$E_{molecule}$(eV)	-134.245	-221.822	-285.714
E_{defect}(eV)	-672.541	-672.541	-672.541
$E_{defect+molecule}$(eV)	-807.469	-895.058	-959.046
E_{int} (eV)	-0.683	-0.695	-0.791

Supplementary Table 2. Fitted parameters for time-resolved photoluminescence decay curves in Fig. 3j. The measured data were fitted using a biexponential decay model:

$$y = y_0 + A_1 e^{-x/\tau_1} + A_2 e^{-x/\tau_2} \quad (3)$$

where the average lifetime τ_{ave} is calculated as

$$\tau = \frac{A_1}{A_1+A_2}\tau_1 + \frac{A_2}{A_1+A_2}\tau_2 \quad (4)$$

Samples	τ_1 (ns)	A_1	τ_2 (ns)	A_2	τ_{avg} (ns)
Control	85.2	0.125	237.1	0.875	218.1
with 2APM	144.8	0.146	338.8	0.854	310.5
with 3APM	152.2	0.167	456.3	0.833	405.5
with 4APM	176.1	0.201	630.2	0.799	538.9

Supplementary Table 3. Performance of perovskite solar cells with different 2APM concentrations.

	V_{OC} (V)	J_{SC} (mA cm^{-2})	*FF* (%)	PCE (%)
Control	1.155	25.42	81.87	24.04
0.05 mg/mL	1.161	25.45	82.12	24.32
0.1 mg/mL	1.168	25.55	82.91	24.74
0.3 mg/mL	1.159	25.48	81.95	24.20
0.5 mg/mL	1.147	25.43	80.67	23.53

Supplementary Table 4. Performance of perovskite solar cells with different 3APM concentrations.

	V_{OC} (V)	J_{SC} (mA cm^{-2})	*FF* (%)	PCE (%)
Control	1.155	25.42	81.87	24.04
0.05 mg/mL	1.169	25.48	82.64	24.61
0.1 mg/mL	1.178	25.67	84.88	25.67
0.3 mg/mL	1.162	25.51	81.82	24.25
0.5 mg/mL	1.158	25.47	81.23	23.95

Supplementary Table 5. Performance of perovskite solar cells with different 4APM concentrations.

	V_{OC} (V)	J_{SC} (mA cm^{-2})	*FF* (%)	PCE (%)
Control	1.155	25.42	81.87	24.04
0.05 mg/mL	1.178	25.58	83.74	25.23
0.1 mg/mL	1.187	25.72	85.95	26.26
0.3 mg/mL	1.174	25.56	82.31	24.69
0.5 mg/mL	1.163	25.51	81.75	24.25

Supplementary Table 6. Photovoltaic parameters for forward and reverse scanning of PSCs without and with 2APM, 3APM and 4APM under 1 sun illumination.

Sample	Scan mode	V_{OC} (V)	J_{SC} (mA cm^{-2})	*FF* (%)	PCE (%)	Hysteresis index [a]
Control	Reverse	1.155	25.42	81.87	24.04	0.023
	Forward	1.129	25.39	80.76	23.15	
with 2APM	Reverse	1.168	25.55	82.91	24.74	0.018
	Forward	1.152	25.42	82.05	24.02	
with 3APM	Reverse	1.178	25.67	84.88	25.67	0.012
	Forward	1.169	25.55	83.95	25.07	
With 4APM	Reverse	1.187	25.72	85.95	26.26	0.007
	Forward	1.181	25.66	85.31	25.85	

[a] Hysteresis index = ($J_{RS}(0.8V_{OC})$-$J_{FS}(0.8V_{OC})$)/$J_{RS}(0.8V_{OC})$.

Supplementary Table 7. Summary of device performance of perovskite solar modules without and with 2APM, 3APM and 4APM. (active area: 14 cm^2)

	V_{OC} (V)	J_{SC} (mA cm^{-2})	*FF* (%)	PCE (%)
Control	10.77	2.376	75.61	19.30
with 2APM	10.95	2.395	78.25	20.52
with 3APM	11.25	2.402	81.61	22.05
with 4APM	11.56	2.406	83.23	23.16

Supplementary Table 8. Comparison of the photovoltaic performance of 20 devices for Control, 2APM-, 3APM- and 4APM-treated devices. (active area: 0.09 cm^2)

Sample	V_{OC} (V)	J_{SC} (mA cm^{-2})	FF (%)	PCE (%)
Control	1.153±0.0033	25.35±0.13	80.75±1.07	23.61±0.37
with 2APM	1.161±0.0035	25.42±0.14	82.05±0.72	24.21±0.35
with 3APM	1.173±0.0034	25.56±0.11	84.21±0.64	25.24±0.34
With 4APM	1.184±0.0032	25.63±0.08	85.37±0.55	25.91±0.27
