# Supplementary material for: Three-dimensional distribution of amino groups improves perovskite crystallization and defect passivation in high-performance photovoltaics
Source: Nat Commun. 2026 Jul 25;17:9077. doi: 10.1038/s41467-026-75990-x (PMC13518884; doi:10.1038/s41467-026-75990-x)
Supplement: Supplementary file 2 [file 41467_2026_75990_MOESM2_ESM.pdf]

nature portfolio

Corresponding author(s): Zhongjin Shen

Solar Cells Reporting Summary

Nature Portfolio wishes to improve the reproducibility of the work that we publish. This form is intended for publication with all accepted papers reporting the characterization of photovoltaic devices and provides structure for consistency and transparency in reporting. Some list items might not apply to an individual manuscript, but all fields must be completed for clarity.

For further information on Nature Research policies, including our data availability policy, see Authors & Referees.

Experimental design

Please check the following details are reported in the manuscript, and provide a brief description or explanation where applicable.

1. Dimensions

Area of the tested solar cells

- [x] Yes — 0.25 cm2
- [ ] No — *Explain why this information is not reported/not relevant.*

Method used to determine the device area

- [x] Yes — The cells with an active area of 0.5 cm x 0.5 cm
- [ ] No — *Explain why this information is not reported/not relevant.*

2. Current-voltage characterization

Current density-voltage (J-V) plots in both forward and backward direction

- [x] Yes — Figure5b and Supplementary Figure S25
- [ ] No

Voltage scan conditions

- [x] Yes — Device Characterization in Methods section
- [ ] No — *Explain why this information is not reported/not relevant.*

Test environment

- [x] Yes — Solar cells were tested at room temperature (25 °C) and in ambient air conditions
- [ ] No — *Explain why this information is not reported/not relevant.*

Protocol for preconditioning of the device before its characterization

- [ ] Yes — *Provide a description of the protocol.*
- [x] No — We did not use any preconditioning.

Stability of the J-V characteristic

- [x] Yes — Figure 5c
- [ ] No — *Explain why this information is not reported/not relevant.*

3. Hysteresis or any other unusual behaviour

Description of the unusual behaviour observed during the characterization

- [x] Yes — Figure5b, Supplementary Figure S25 and the photovoltaic parameters are summarized in Supplementary Table S6
- [ ] No — *Explain why this information is not reported/not relevant.*

Related experimental data

- [x] Yes — Figure5b, Supplementary Figure S25 and the photovoltaic parameters are summarized in Supplementary Table S6
- [ ] No — *Explain why this information is not reported/not relevant.*

4. Efficiency

External quantum efficiency (EQE) or incident photons to current efficiency (IPCE)

- [x] Yes — Figure 5e
- [ ] No — *Explain why this information is not reported/not relevant.*

A comparison between the integrated response under the standard reference spectrum and the response measure under the simulator

- [x] Yes — It is described in the manuscript, at the device performance
- [ ] No — *Explain why this information is not reported/not relevant.*

For tandem solar cells, the bias illumination and bias voltage used for each subcell

☐ Yes ☒ No

Provide a description of the measurement conditions.

No tandem cells investigated.

5. Calibration

Light source and reference cell or sensor used for the characterization

☒ Yes ☐ No

Device Characterization in Methods section. The intensity of one-sun AM 1.5G illumination was calibrated using a Si-reference cell certified by the National Renewable Energy Laboratory.

Explain why this information is not reported/not relevant.

Confirmation that the reference cell was calibrated and certified

☒ Yes ☐ No

The reference cell was certified by the National Renewable Energy Laboratory and calibrated on 23 April 2025

Explain why this information is not reported/not relevant.

Calculation of spectral mismatch between the reference cell and the devices under test

☒ Yes ☐ No

The spectral mismatch factor is 0.986, which was determined from the solar simulator spectrum, the AM1.5 spectrum and the EQE spectra of the test and reference cells.

Explain why this information is not reported/not relevant.

6. Mask/aperture

Size of the mask/aperture used during testing

☒ Yes ☐ No

0.09 cm2

Explain why this information is not reported/not relevant.

Variation of the measured short-circuit current density with the mask/aperture area

☐ Yes ☒ No

Report the difference in the short-circuit current density values measured with the mask and aperture area.

This has been investigated in our group intensively in the past, the mask area used here has been a standard within the group since many years.

7. Performance certification

Identity of the independent certification laboratory that confirmed the photovoltaic performance

☐ Yes ☒ No

Identify the independent certification laboratory.

We do not report a new record efficiency and the PCE is not the key quantity investigated in this work. The solar simulator and the test cell used are industry standard, efficiencies have been confirmed by other labs and by certification institutes in the past.

A copy of any certificate(s)

☐ Yes ☒ No

Certificate copies should be provided in the Supplementary information. Please state the supplementary item number.

see above

8. Statistics

Number of solar cells tested

☒ Yes ☐ No

20 cells were tested for each condition, Figure 5f and Supplementary Figure S26

Explain why this information is not reported/not relevant.

Statistical analysis of the device performance

☒ Yes ☐ No

Figure 5f, Supplementary Figure S26 and Table S8

Explain why this information is not reported/not relevant.

9. Long-term stability analysis

Type of analysis, bias conditions and environmental conditions

☒ Yes ☐ No

Figure 5h, Figure 5i and Supplementary Figure S29

Explain why this information is not reported/not relevant.
