# Supplementary material for: Three-dimensional distribution of amino groups improves perovskite crystallization and defect passivation in high-performance photovoltaics
Source: Nat Commun. 2026 Jul 25;17:9077. doi: 10.1038/s41467-026-75990-x (PMC13518884; doi:10.1038/s41467-026-75990-x)
Supplement: Supplementary file 3 [file 41467_2026_75990_MOESM3_ESM.pdf]

nature portfolio

Peer Review File

Three-dimensional distribution of amino groups improves perovskite crystallization and defect passivation in high-performance photovoltaics

Corresponding Author: Dr Zhongjin Shen

This file contains all reviewer reports in order by version, followed by all author rebuttals in order by version.

Version 0:

Reviewer comments:

Reviewer #1

(Remarks to the Author)
This work investigates the influence of aniline-based molecules with distinct spatial configurations on defect passivation and perovskite film crystallization in perovskite solar cells (PSCs) and modules. The authors demonstrate that increasing molecular dimensionality progressively enhances defect passivation, suppresses non-radiative recombination, and improves film crystallinity, resulting in systematic performance gains. Compared with control devices, PSCs and modules based on the 3D molecule TAAM achieve champion PCEs of 26.26% and 23.16%, respectively, while retaining over 95% of their initial efficiency after 1000 h of MPPT operation under a N_2 atmosphere at 65 °C. Overall, this study is of potential interest to Nature Communications, provided the following minor revisions are addressed before it can be considered for publication.

1. Although the electrostatic potential (ESP) map in Figure 1b includes a color scale, the corresponding physical units are not specified. This omission limits the quantitative interpretability of the data. The authors should include appropriate unit labels on the color bar.
2. The TOF-SIMS data indicate pronounced enrichment of the additive molecules at the top surface of the perovskite film, and AFM-IR further suggests that this distribution is laterally uniform. However, the manuscript does not provide a mechanistic explanation for the preferential migration and accumulation of these molecules at the surface during film formation. This point should be clarified.
3. The indexing of XRD diffraction peaks in Figure 2i appears inconsistent with standard conventions. In particular, assigning peaks at ~14.1°, 20°, 24.5°, and 28° to the (001), (011), (111), and (002) planes deviates from the commonly adopted (100)-family indexing used in perovskite literature. The authors should carefully re-examine and correct the peak assignments throughout the manuscript. A related inconsistency is found in Figure S8, where the label refers to the (001) plane while the caption discusses the FWHM of the (100) peak. This discrepancy should be resolved to ensure internal consistency.
4. The claim of a preferred (100) orientation in the TAAM-modified perovskite film (Figure 2i) requires more direct experimental evidence. GIWAXS measurements should be provided to substantiate this assertion, and the discussion should be revised accordingly.
5. While the DFT results in Figure 1c suggest stronger Pb^{2+} coordination for TAAM compared to the other molecules, the computational methodology is insufficiently described. The authors should clearly define the binding energy expression and report the calculated values for all molecules to enable quantitative comparison.
6. Figure 1d would benefit from a more quantitative analysis of charge transfer. Providing isosurface values and integrated charge data in the Supplementary Information would improve reproducibility and allow for more rigorous comparison.
7. The operational stability data are currently limited to small-area PSC devices. Given that module performance is a key claim of this work, additional stability measurements at the module level should be included.

Reviewer #2

(Remarks to the Author)
This manuscript compares the influence of molecular spatial configuration on film crystallization and defect passivation in

perovskite solar cells. It is claimed that the 3D-structured molecule (TAAM) is advantageous in enhancing defect passivation, perovskite film crystallization, and interfacial energy level alignment over the other two molecules with lower dimensions, thereby yielding in the best device performance.The point is interesting and resulting device efficiencies and stability are impressive.I believe it is an authoritative and inspiring article, and will draw broad readership.I would recommend its acceptance for publication in Nature Communications provided that the authors addresses following issues:

1. The authors attribute the superior passivation capability of TAAM primarily to its three-dimensional spatial configuration. However, compared to the other two molecules, TAAM also bears more functional groups. Could this also contribute to its stronger passivation ability? Furthermore, the enhanced electron cloud density of the groups might result from interactions among multiple groups, rather than solely from the simple stereostructure. It is recommended to revise the related descriptions accordingly.

2. Cross-sectional SEM images are currently absent for samples subjected to different molecular treatments. The authors should provide those data for all relevant samples to enable a systematic and detailed comparison of film morphology characteristics across the control and modified devices.

3. Figure 1b shows significant differences in the calculated dipole moments among the three molecules, but the main text does not discuss the potential implications of these dipole moment variations for perovskite film formation or device physics.

4. In Figure 5g, the voltage generated by the MEDE-modified device under 1 sun illumination appears inconsistent with the corresponding open-circuit voltage reported in the J–V curve. Please explain the reason for this discrepancy.

5. Although a champion PCE of 26.26% is reported for the PSC, neither the main text nor the Supplementary Information specifies the device area or the active area used for this measurement. The authors need to provide the relevant information as well as the aperture mask used during characterization.

Reviewer #3

(Remarks to the Author)
In this manuscript, Jiang et al. reported three aniline-based molecules, so-called 4,4'-methylenedianiline (MEDE), tris(4-aminophenyl)methane (TSAM), and tetra(4-aminophenyl)methane (TAAM), with different spatial configurations as in-situ modulators incorporated into the perovskite precursor solution. In the three molecules, TAAM with a 3D architecture most effectively suppresses uncoordinated Pb^{2+} defects, enhances the perovskite film crystallinity, and optimizes the interfacial energy-level alignment between the perovskite and the HTL. The TAAM-incorporated small-area perovskite solar cells and mini-modules achieved efficiencies of 26.26% and 23.16%, respectively, along with notable improvement in operational stability. This work can be considered for publication in Nature Communications after the points listed below are addressed.
1. The title of this manuscript needs to be revised to clearly show the specific difference in the spatial configuration of the three molecules.
2. The abbreviations of the three molecules, which do not reflect their structural characteristics, are suggested to be changed.
3. The NMR peak shifts of the amino groups in the three aniline molecules are described as “shifted upfield”. However, in Figures 2a-c and Figure S5, the chemical shifts of the amino groups consistently move toward lower numerical values. Please clarify how the direction of the shift is defined.
4. In Figure 2f, the N–H peak shift does not appear to correspond to the value of "Δ = 14 cm^{-1}" stated in the main text. The authors should verify whether this discrepancy reflects a labeling error in the figure or an inaccuracy in the reported value and provide corrected data or a revised figure as appropriate.
5. It is stated that all three aniline molecules are incorporated as bulk-phase additives within the perovskite film. However, both AFM-IR and TOF-SIMS analyses show strong signals from these molecules on the film surface. Please explain the cause of this surface signal.
6. The team "PEC" in the sentence “retaining 93% of their initial PEC after 2000 h of aging” appears to be an error. The authors should carefully check the entire manuscript to identify and correct any similar typographical or terminology issues.
7. The reduced surface potential variation observed in KPFM measurement is interpreted as evidence of lower defect density. However, KPFM signals are inherently sensitive not only to defect states but also to local work function variations and surface dipole moments.
8. Please also clarify how the phenylamine-based molecules contribute to the observed increase in VOC. Specifically, it should be clarified whether the VOC enhancement arises from the suppressed non-radiative recombination, the modified interfacial energy-level alignment, or their synergistic effects.

Reviewer #4

(Remarks to the Author)
This study finds that the three-dimensional (3D) molecule TAAM, due to its multidentate aniline groups, most effectively suppresses uncoordinated Pb2+ defects and improves the crystallinity of perovskite films. Furthermore, the TAAM molecule demonstrates good device performance in both perovskite solar cells and modules (14 cm2) with superior operational stability under MPP tracking conditions. These findings provide an important reference for the rational selection of in-situ modification molecules in high-performance perovskite photovoltaics.Therefore, I am willing to recommend its publication in

this journal after addressing the following revisions.
1. The paper indirectly infers that TAAM effectively suppresses uncoordinated Pb2+ defects based on results such as improved XRD crystallinity and homogenized surface potential in KPFM. However, it lacks direct quantitative characterization of changes in trap density. It is recommended to supplement tests such as space-charge-limited current (SCLC) to quantitatively compare the trap densities of devices modified with different molecules.
2. The paper compares three molecules—MEDE, TSAM, and TAAM—with functional group (-NH2) counts of 2, 3, and 4, respectively. Please explain how the authors more fairly compare the influence of "spatial configuration" rather than merely the influence of the "number of functional groups."
3. The authors optimized the concentrations of all three additives and achieved the best device performance with the concentration of 0.1 mg/mL. Please explain why the higher or lower concentrations cause the inferior device performance.
4. The differential charge distribution of the 3D molecule TAAM shows significantly enhanced charge transfer performance. Please explain the reason for this enhancement.
5. The author mentioned in the discussion that the molecules were incorporated into the perovskite bulk phase, but the TOF-SIMS results clearly show strong signals of the molecules still present on the film surface. Please explain why this phenomenon occurs and provide a clearer description of the actual distribution of the molecules.
6. Why did the authors choose silver as the electrode instead of gold? I am curious whether there might also be some differences in photovoltaic parameters between the two, and I would like the authors to provide some comparisons and reasonable explanations.
7. Please carefully review the grammar throughout the manuscript and thoroughly proofread the spelling to ensure accuracy and consistency. It would be greatly appreciated if the authors could pay close attention to any typographical errors, awkward phrasing, or grammatical inconsistencies, and make the necessary revisions to improve the overall clarity and professionalism of the manuscript.

Version 1:

Reviewer comments:

Reviewer #1

(Remarks to the Author)
The manuscript was revised accordingly, I would recommend to accept it without further revision.

Reviewer #2

(Remarks to the Author)
This work can be accepted as it is, without any further revision.

Reviewer #3

(Remarks to the Author)
The authors have well addressed the issues from the reviewers. The revised manuscript is now accepted for publication as is.

Reviewer #4

(Remarks to the Author)
The authors have addressed most of the issues raised in a satisfactory manner. The responses are appropriate and supported by additional data and discussion where necessary. Given these revisions, the manuscript can be considered for publication.

Open Access This Peer Review File is licensed under a Creative Commons Attribution 4.0 International License, which permits use, sharing, adaptation, distribution and reproduction in any medium or format, as long as you give appropriate credit to the original author(s) and the source, provide a link to the Creative Commons license, and indicate if changes were made.
In cases where reviewers are anonymous, credit should be given to 'Anonymous Referee' and the source.
The images or other third party material in this Peer Review File are included in the article's Creative Commons license, unless indicated otherwise in a credit line to the material. If material is not included in the article's Creative Commons license and your intended use is not permitted by statutory regulation or exceeds the permitted use, you will need to obtain permission directly from the copyright holder.
To view a copy of this license, visit https://creativecommons.org/licenses/by/4.0/

We are grateful to the reviewers for the time and effort that they spent on our manuscript. We have diligently addressed their concerns and comments in our revised manuscript. Our point-by-point responses (in blue) to the reviewers' comments (in **black**) are as below. Changes made to the revised manuscript and supplementary information are highlighted in yellow.

REVIEWER COMMENTS

Reviewer #1 (Remarks to the Author):

This work investigates the influence of aniline-based molecules with distinct spatial configurations on defect passivation and perovskite film crystallization in perovskite solar cells (PSCs) and modules. The authors demonstrate that increasing molecular dimensionality progressively enhances defect passivation, suppresses non-radiative recombination, and improves film crystallinity, resulting in systematic performance gains. Compared with control devices, PSCs and modules based on the 3D molecule TAAM achieve champion PCEs of 26.26% and 23.16%, respectively, while retaining over 95% of their initial efficiency after 1000 h of MPPT operation under a N_2 atmosphere at 65 °C. Overall, this study is of potential interest to Nature Communications, provided the following minor revisions are addressed before it can be considered for publication.

Response: We sincerely appreciate the reviewer's positive and constructive comments, which have been invaluable in improving the quality of our manuscript.

1. Although the electrostatic potential (ESP) map in Figure 1b includes a color scale, the corresponding physical units are not specified. This omission limits the quantitative interpretability of the data. The authors should include appropriate unit labels on the color bar.

Response: We sincerely appreciate the reviewer for the comments. We have added the electrostatic potential unit (eV) to Figure 1b in the manuscript, as presented in Figure R1.

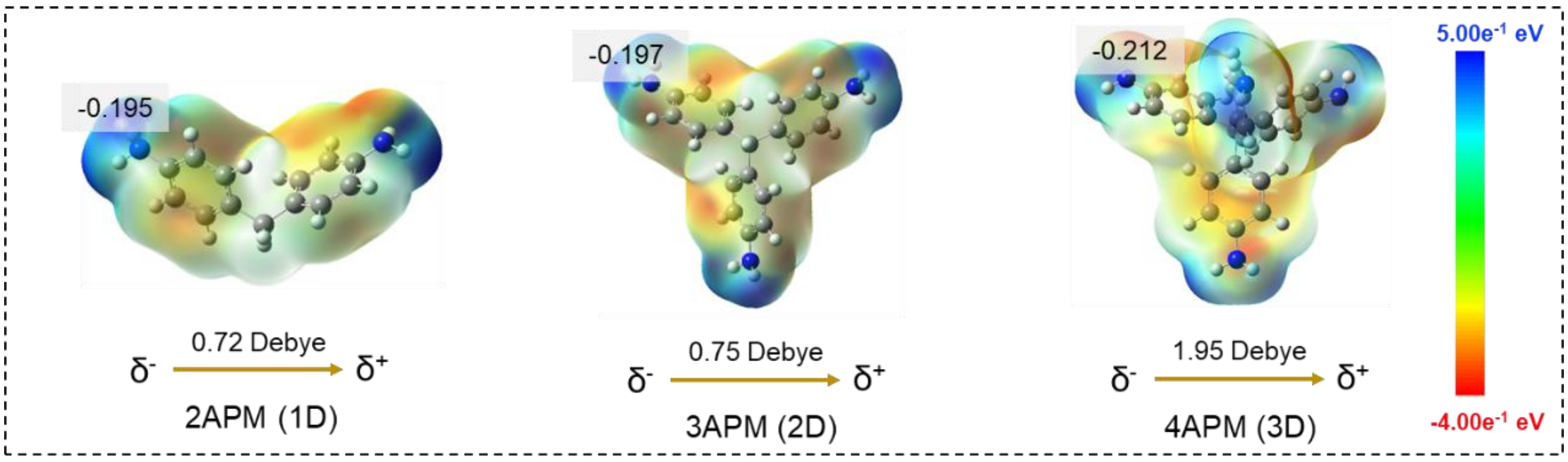

Figure R1. Calculated ESP profiles for 2APM, 3APM and 4APM molecules.

2. The TOF-SIMS data indicate pronounced enrichment of the additive molecules at the top surface of the perovskite film, and AFM-IR further suggests that this distribution is laterally uniform. However, the manuscript does not provide a mechanistic explanation for the preferential migration and accumulation of these molecules at the surface during film formation. This point should be clarified.

Response: Thanks for the reviewer's thoughtful and constructive comments. The surface enrichment of additive molecules at the top of the film arises primarily from the combined effects of crystallization-induced segregation and defect-trapping. During perovskite crystal nucleation and growth, uncoordinated molecules are expelled from the growing perovskite lattice and accumulate at interfacial regions, particularly the air/film interface (top surface) and grain boundaries (Adv. Energy Mater. e04647 (2025).; ACS Energy Lett. 10, 3325–3334 (2025)). Furthermore, as demonstrated in this work through DFT simulations, NMR, and FTIR characterization, the amino groups (–NH_2) of these molecules exhibit strong binding affinity for under-coordinated Pb^{2+} ions. Since the top surface and grain boundaries of perovskite films host the highest density of such under-coordinated Pb^{2+} defect sites, the expelled molecules preferentially anchor to these regions through coordination interactions. This synergistic accumulation mechanism, driven by both physical segregation during crystallization and chemical affinity for surface defects, accounts for the strong molecular signals detected at the surface by TOF-SIMS.

To make this more clear, we added and modified these sentences in the manuscript:

"…. Depth-profile analysis reveals that the additive molecules are partially enriched at the upper surface of the perovskite layer (Figure S22), owing to the synergistic effects of physical segregation during perovskite crystallization and molecular chemical affinity for surface defects, which favors effective passivation of under-coordinated Pb^{2+} defects at the surface and grain boundaries of perovskite films. [45, 46] "

3. The indexing of XRD diffraction peaks in Figure 2i appears inconsistent with

standard conventions. In particular, assigning peaks at ~14.1°, 20°, 24.5°, and 28° to the (001), (011), (111), and (002) planes deviates from the commonly adopted (100)-family indexing used in perovskite literature. The authors should carefully re-examine and correct the peak assignments throughout the manuscript. A related inconsistency is found in Figure S8, where the label refers to the (001) plane while the caption discusses the FWHM of the (100) peak. This discrepancy should be resolved to ensure internal consistency.

Response: We thank the reviewer for this valuable suggestion. The characteristic peak assignments and nomenclature of perovskite have been re-labeled accordingly in Figures R2–R3, with corresponding revisions made to Figure 2i in the main text and Figure S12 in the Supporting Information.

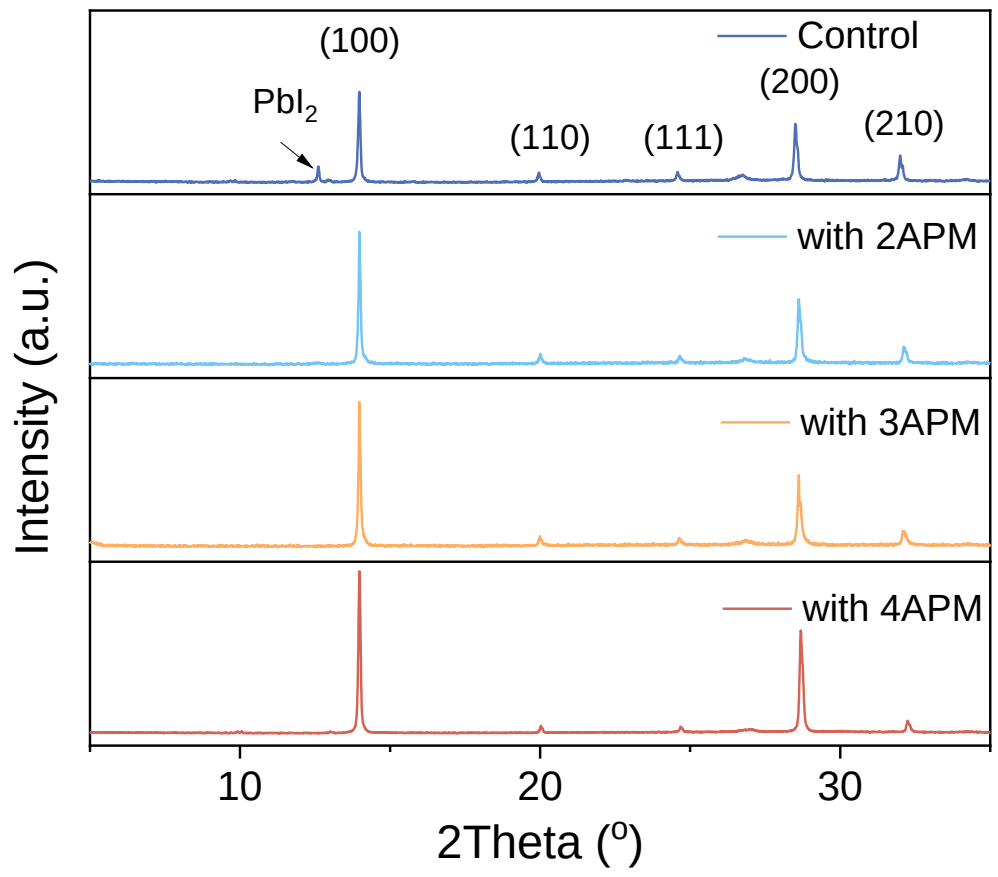

Figure R2. XRD patterns of perovskite films with different additives.

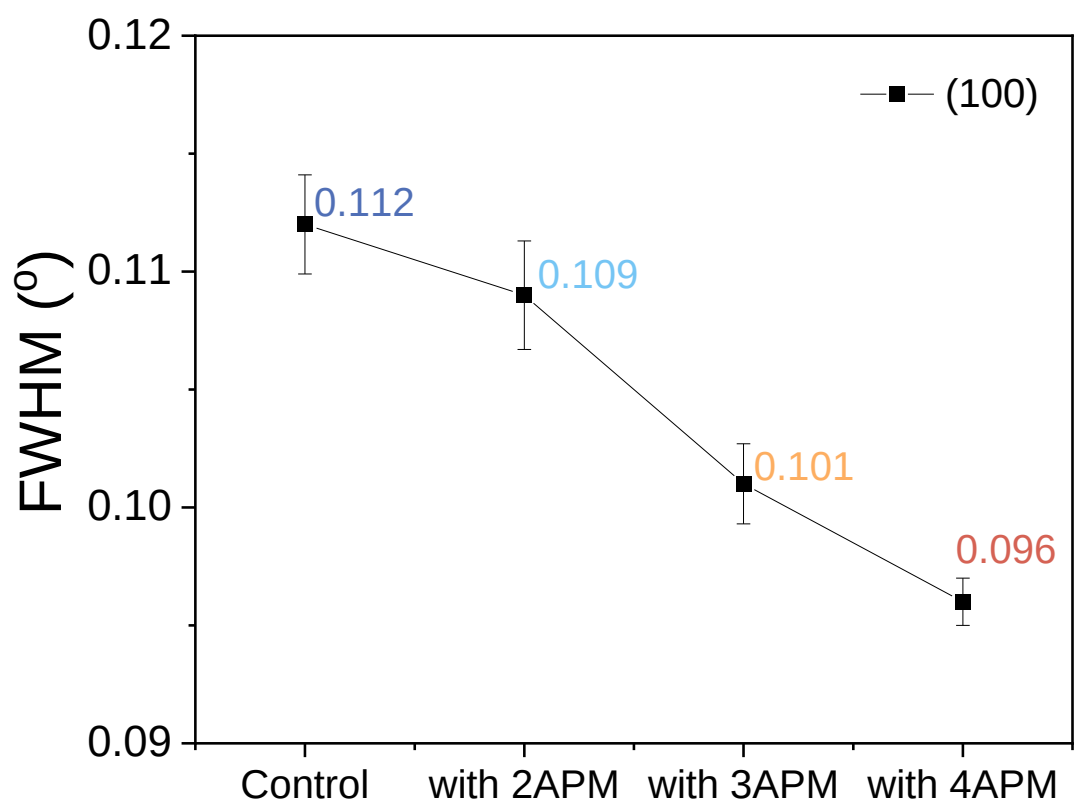

Figure R3. The full width at half maximum (FWHM) of the (100) diffraction peak. (among 3 perovskite films for each condition).

4. The claim of a preferred (100) orientation in the TAAM-modified perovskite film (Figure 2i) requires more direct experimental evidence. GIWAXS measurements should be provided to substantiate this assertion, and the discussion should be revised accordingly.

Response: We appreciate the reviewer raising this important point. Grazing-incidence wide-angle X-ray scattering (GIWAXS) was performed to verify the preferred (100) crystallographic orientation. Compared to the control, 2APM-, and 3APM-treated perovskite films, the 4APM-treated perovskite film exhibits a markedly enhanced diffraction intensity along the out-of-plane (q_z) direction at q ≈ 1 $Å^{-1}$, corresponding to the (100) plane, indicating a stronger preferred orientation, which is consistent with the XRD results presented in the manuscript.

To make this more clear, we added these sentences in the manuscript:

"…. To further corroborate these findings, grazing-incidence wide-angle X-ray scattering (GIWAXS) measurements were conducted. Compared to the control, 2APM- and 3APM-treated perovskite films, the 4APM-treated sample exhibits enhanced diffraction intensity along the out-of-plane (q_z) direction at q≈1 $Å^{-1}$ (Figures S13 and S14), which corresponds to the (100) crystal plane, indicating stronger preferential orientation, in good agreement with the XRD results discussed above."

We also added Figures R4-R5 in Supporting Information as Figures S13 and S14.

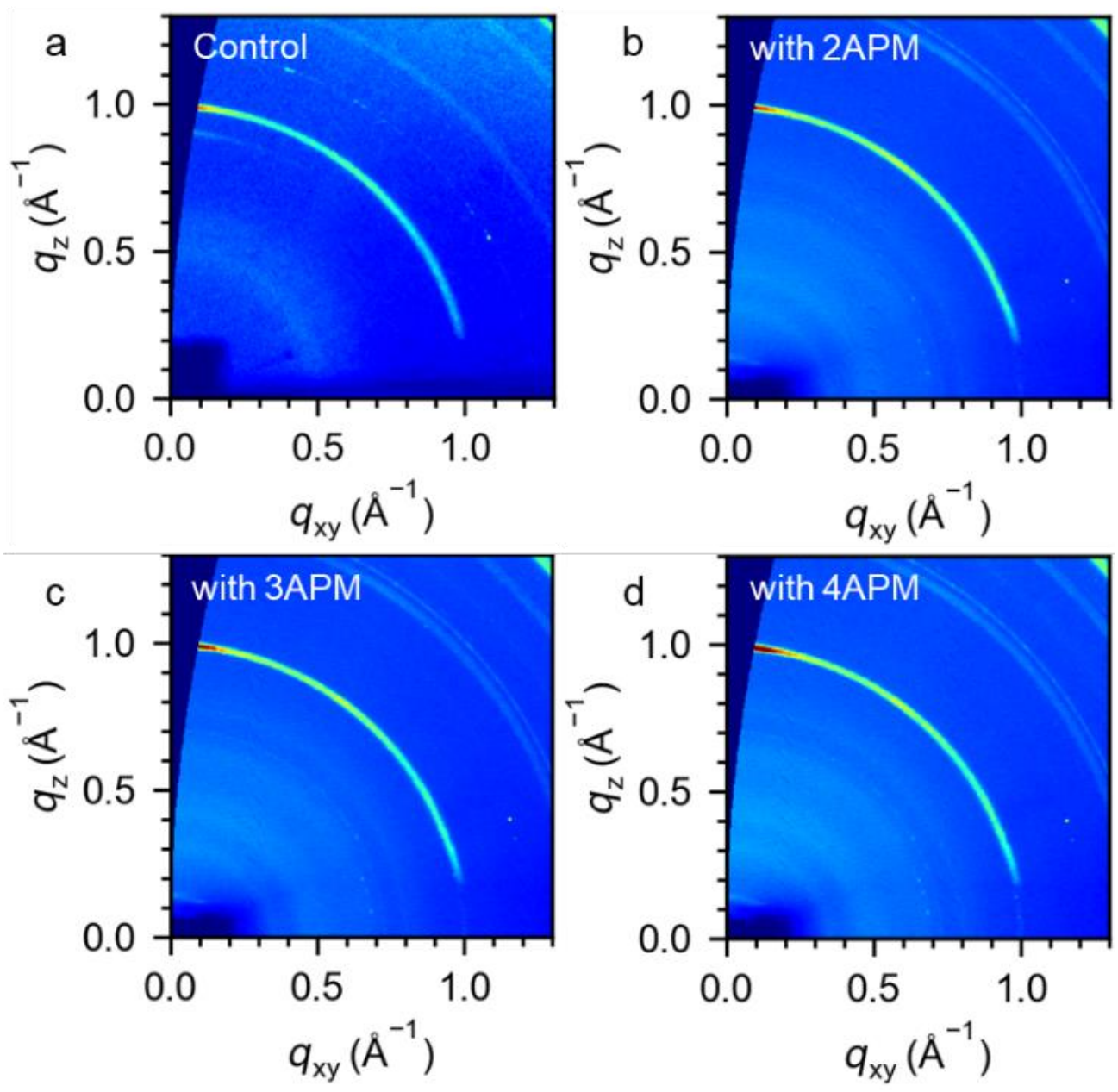

Figure R4. (a-d) GWIAXS patterns of perovskite films without and with 2APM, 3APM and 4APM treatment.

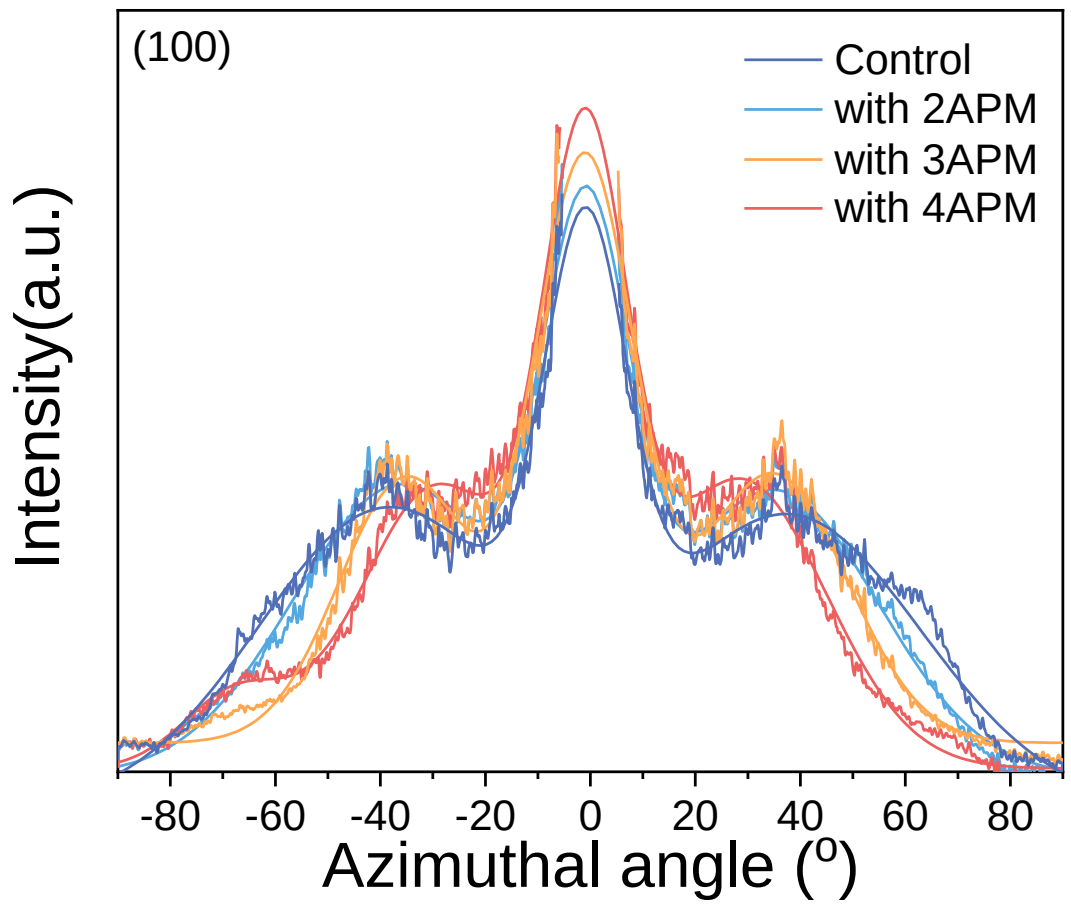

Figure R5. The plots of perovskite film of (100) facet along the ring at the range about 0.9 - 1.05 $Å^{-1}$.

5. While the DFT results in Figure 1c suggest stronger Pb^{2+} coordination for TAAM compared to the other molecules, the computational methodology is insufficiently described. The authors should clearly define the binding energy expression and report the calculated values for all molecules to enable quantitative comparison.

Response: We appreciate the reviewer raising this issue. More complete computational details have been provided in the Supporting Information. The interaction energies of the three molecules on the $FAPbI_3$ (100) surface were calculated, with iodine vacancies introduced to generate undercoordinated Pb^{2+} sites, simulating the predominant trap states in perovskite films. Electronic exchange-correlation interactions were described using the Perdew-Burke-Ernzerhof (PBE) functional method under the generalized gradient approximation (GGA-PBE). In all calculations, the plane-wave cutoff energy for electronic wavefunctions was set to 400 eV. The Brillouin zone sampling employed a (2×2×1) Monkhorst-Pack k-point mesh for geometric structure relaxation. To avoid interactions between adjacent layers, a vacuum layer of 20 Å was set along the z-axis. The dipole moment correction function remained enabled. The van der Waals forces in the structure were corrected using Grimme's DFT-D3 method.

To make this more clear, we added Table R1 as Table S1, and the definition of the interaction energies in the Supporting information:

"…..The interaction energies are defined as:

$E_{int} = E_{defect+molecule} - E_{defect} - E_{molecule}$"

Table R1. The interaction energies between molecules and perovskite surface defects.

	2APM	3APM	4APM

$E_{molecule}$(eV)	-134.245	-221.822	-285.714
E_{defect}(eV)	-672.541	-672.541	-672.541
$E_{defect+molecule}$(eV)	-807.469	-895.058	-959.046
E_{int} (eV)	-0.683	-0.695	-0.791

We also added and modified these sentences in the manuscript:

"….. As shown in Figures 1c, S2 and Table S1, the 3D molecule 4APM exhibits the strongest interaction energy of −0.791 eV, compared to −0.695 eV for the 2D molecule 3APM."

6. Figure 1d would benefit from a more quantitative analysis of charge transfer. Providing isosurface values and integrated charge data in the Supplementary Information would improve reproducibility and allow for more rigorous comparison.

Response: We appreciate the reviewers for pointing this out. More complete computational details have been provided in the Supporting Information, along with one-dimensional integral plots of differential charge density (Figure R6). The isosurface values for the charge density difference plots is set at 0.0004 e/Å^3. Compared to 2APM and 3APM, 4APM exhibits more pronounced electron accumulation (positive) and electron depletion (negative), indicating more intense charge transfer and redistribution at the 4APM/perovskite interface. This reflects stronger chemical interactions between the 4APM molecules and surface Pb^{2+} atoms, which in turn facilitate more effective defect passivation and a more favorable interfacial energy level alignment, ultimately accounting for the superior device performance observed for 4APM-treated films.

To make this clearer, we added Figure R6 in the Supporting Information as Figure S5. In addition, we modified these sentences in the manuscript:

"…. Charge density difference plots further reveal that 4APM facilitates greater electron density sharing toward Pb^{2+} relative to the other additives (Figure 1d and Figure S5)."

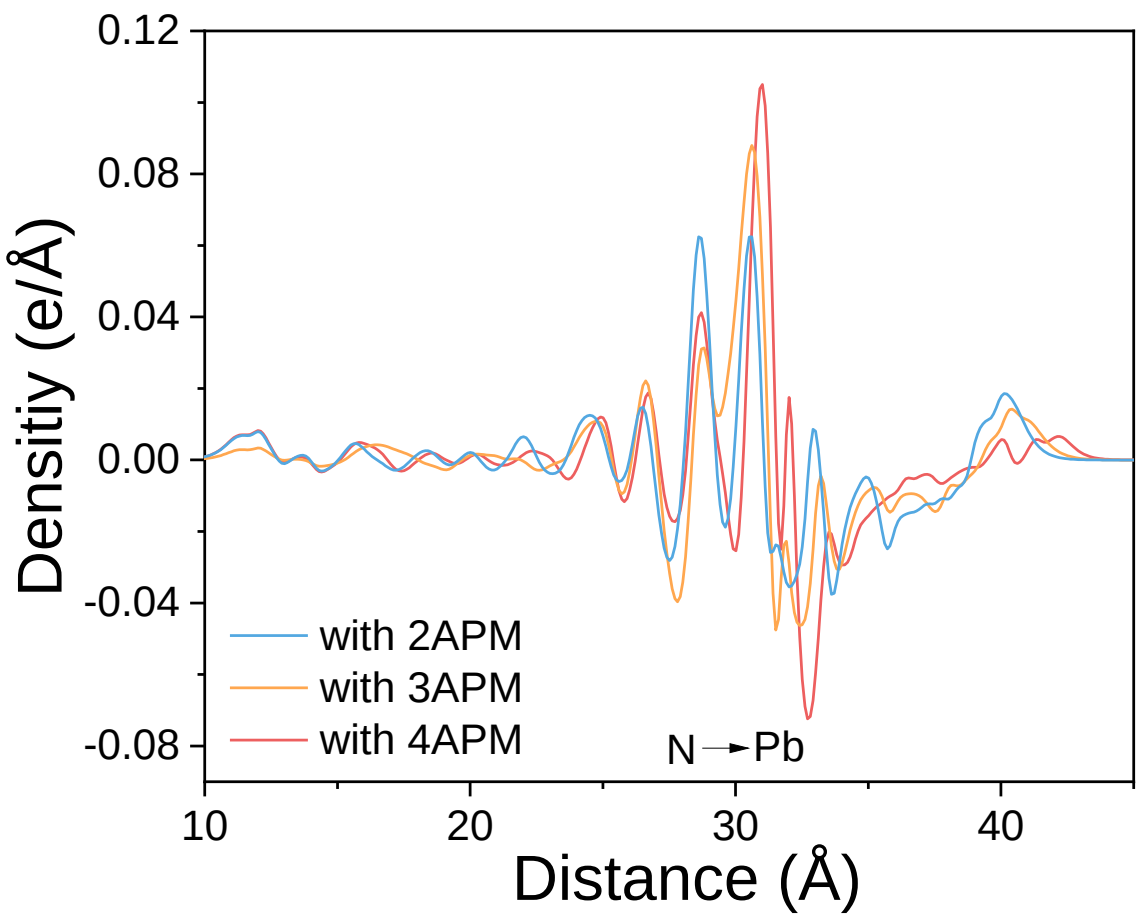

Figure R6. Longitudinal integration image of differential charge density. The isosurface values for the charge density difference plots is set at 0.0004 e/Å^3. Compared to 2APM and 3APM, 4APM exhibits more pronounced electron accumulation (positive) and electron depletion (negative), indicating more intense charge transfer and redistribution at the 4APM/perovskite interface. This reflects stronger chemical interactions between the 4APM molecules and surface Pb^{2+} atoms, which in turn facilitate more effective defect passivation and a more favorable interfacial energy level alignment.

7. The operational stability data are currently limited to small-area PSC devices. Given that module performance is a key claim of this work, additional stability measurements at the module level should be included.

Response: We thank the reviewer for this valuable suggestion. In response, operational stability for the encapsulated perovskite solar modules (PSMs) under continuous illumination at 65 °C in ambient air have been included, as shown in Figure R7. After 600 hours of aging, the 4APM-treated PSM retains 92% of its initial PCE, whereas the control device lost 30% of its initial efficiency.

To make this more clear, we added the stability of the PSMs in the Supporting Information as Figure S29, and added these sentences in the manuscript:

"……Furthermore, we investigated the operational stability of the encapsulated PSM under continuous light illumination at 65 °C in ambient air (Figure S29). After 600 hours, the 4APM-treated PSM retained 92% of its initial PCE, whereas the control device lost 30% of its initial PCE, further underscoring the superior long-term operational stability imparted by the 3D molecular modification strategy."

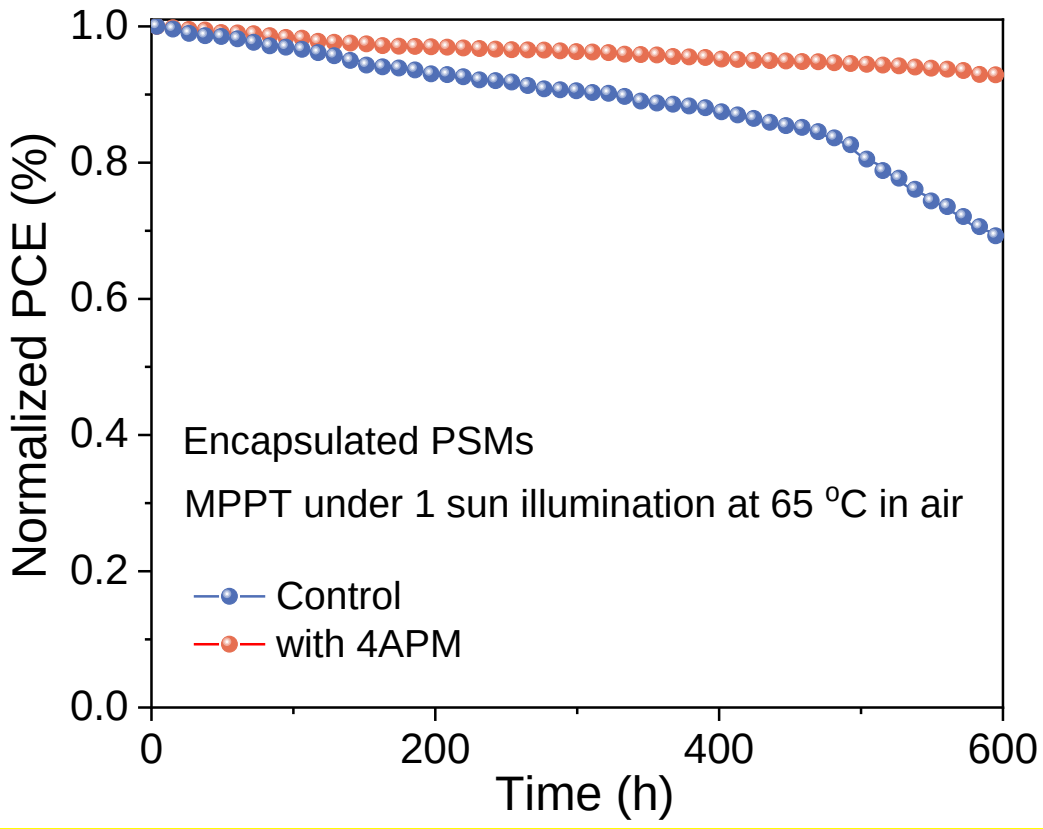

Figure R7. Operational stability of the encapsulated PSMs under 1-sun MPPT conditions at 65 °C in ambient air.

Reviewer #2 (Remarks to the Author):

This manuscript compares the influence of molecular spatial configuration on film crystallization and defect passivation in perovskite solar cells. It is claimed that the 3D-structured molecule (TAAM) is advantageous in enhancing defect passivation, perovskite film crystallization, and interfacial energy level alignment over the other two molecules with lower dimensions, thereby yielding in the best device performance. The point is interesting and resulting device efficiencies and stability are impressive. I believe it is an authoritative and inspiring article, and will draw broad readership. I would recommend its acceptance for publication in Nature Communications provided that the authors addresses following issues:

We thank the reviewer for the overall positive evaluation of this work. We greatly appreciate the reviewer's constructive comments to help improve the quality of our manuscript.

1. The authors attribute the superior passivation capability of TAAM primarily to its three-dimensional spatial configuration. However, compared to the other two molecules, TAAM also bears more functional groups. Could this also contribute to its stronger passivation ability? Furthermore, the enhanced electron cloud density of the groups might result from interactions among multiple groups, rather than solely from the simple stereo structure. It is recommended to revise the related descriptions accordingly.

Response: We sincerely appreciate the reviewer's thoughtful and constructive suggestions. To further elucidate the respective contributions of functional group number and molecular spatial configuration to passivation capability, we selected a molecule with a more planar amino group distribution, tetrakis(4-aminophenyl)ethylene (4APE), bearing four phenylamine groups, as an additional reference to investigate its interaction with lead defects. Based on electrostatic potential (ESP) calculations (Figure R8), the amino groups in 4APE exhibit an electron cloud density of −0.264, significantly higher than that of the three-dimensional molecule 4APM, indicating that the enhanced electron cloud density arises from cooperative interactions among multiple functional groups rather than from the three-dimensional configuration alone. Nevertheless, the molecular dipole moment of 4APE (0.64 D) is substantially lower than that of 4APM (1.95 D), demonstrating that the three-dimensional spatial configuration plays a decisive role in elevating the molecular dipole moment. Furthermore, 4APE exhibits an interaction energy of −0.727 eV and a defect formation energy of 0.488 eV with the perovskite surface (Figure R9), both of which are inferior to those of 4APM, demonstrating that despite possessing an equivalent number of functional groups, the planar molecular configuration of 4APE results in

weaker overall passivation capability compared to the three-dimensional architecture of 4APM.

The interaction between 4APE and lead defects was systematically investigated by ^{1}H NMR, FTIR, XPS, and SEM measurements. As shown in Figure R10a, the ^{1}H NMR chemical shift of the $–NH_2$ group in 4APE shifts upfield by 0.08 ppm upon addition of PbI_2, which is smaller than that observed for 4APM (0.10 ppm), indicating a weaker coordination interaction between the planar 4APE molecule and Pb^{2+}. Consistently, FTIR measurements reveal that upon mixing with PbI_2, the N–H bending vibration of 4APE red-shifts by 13 cm^{-1} (from 1614 to 1601 cm^{-1}), a smaller shift compared to 16 cm^{-1} for 4APM (Figure R10b). XPS analysis further shows that the Pb 4*f* binding energy shift induced by 4APE (0.13 eV) is smaller than that induced by 4APM (0.16 eV) (Figure R10c). These results indicate that while interactions among multiple functional groups enhance electron cloud density and interaction energy, the three-dimensional configuration of 4APM enables its four $–NH_2$ groups to extend more effectively in space, significantly increasing the molecular dipole moment and consequently affording superior defect passivation capability.

To make this more clear, we added the Figures R8-R10 in the Supporting Information as Figures S9-S11, and added and modified these sentences in the manuscript:

"……Furthermore, another molecule, tetrakis(4-aminophenyl)ethylene (4APE), is also studied as a reference, which possesses the same number yet relatively more planar distribution of amino groups compared to 4APM. [24] The study found that 4APM with its amino groups spatially distributed in a three-dimensional (3D) pattern, relative to the other three molecules, present more obvious enhancements in defect passivation, and crystallinity regulation.…."

"….. To decouple the influence of amino groups quantity from their spatial distribution, we introduced a more planar amino-based molecule 4APE as a structural control (Figures S9-S11). Compared to 4APM, 4APE exhibits a denser electron cloud distribution yet a significantly lower dipole moment, despite containing the same number of amino groups. Both computational and experimental results consistently demonstrate that while the number of amino groups contributes to the interaction energy, the 3D configuration of 4APM enables its four $-NH_2$ groups to extend more freely and uniformly into space, in favor of stronger and more spatially distributed interactions with perovskite defects. This stereoscopically structural advantage amplifies the molecular dipole moment of 4APM relative to the more planar 4APE, thereby improving its defect passivation capability beyond what the count of functional groups alone can achieve.……"

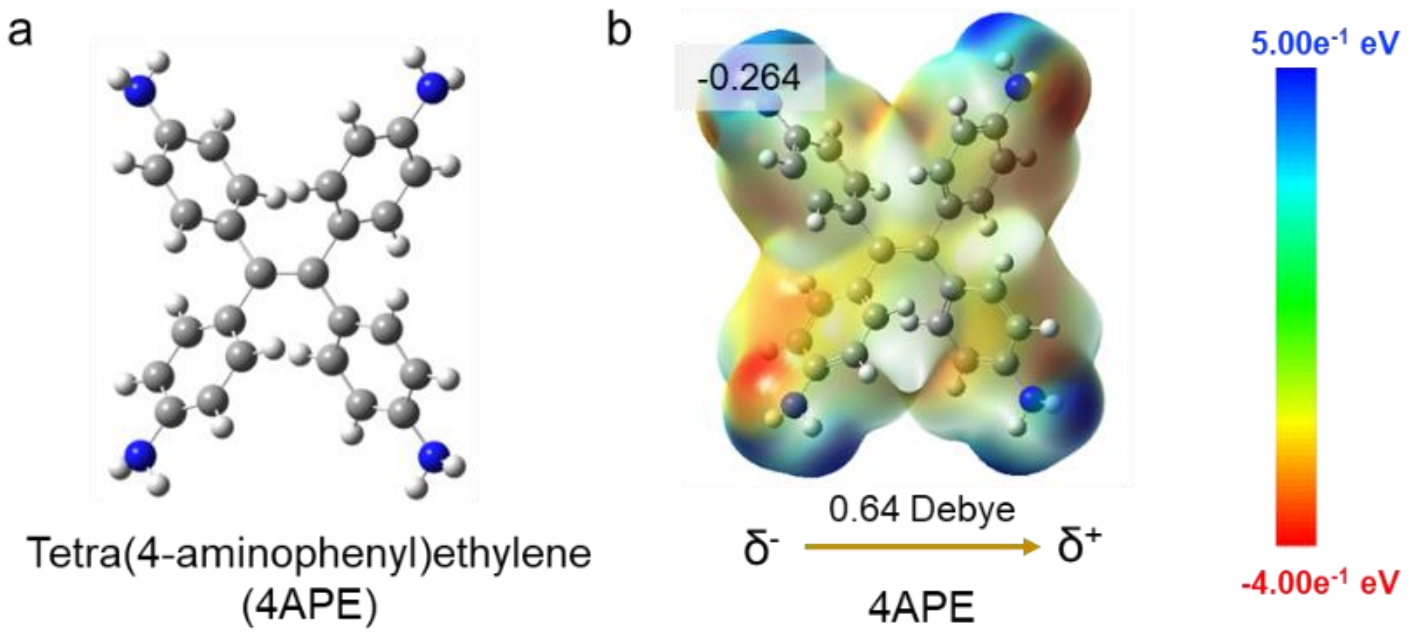

Figure R8. (a) molecular structure of 4APE. (b) Calculated ESP profiles for 4APE molecule.

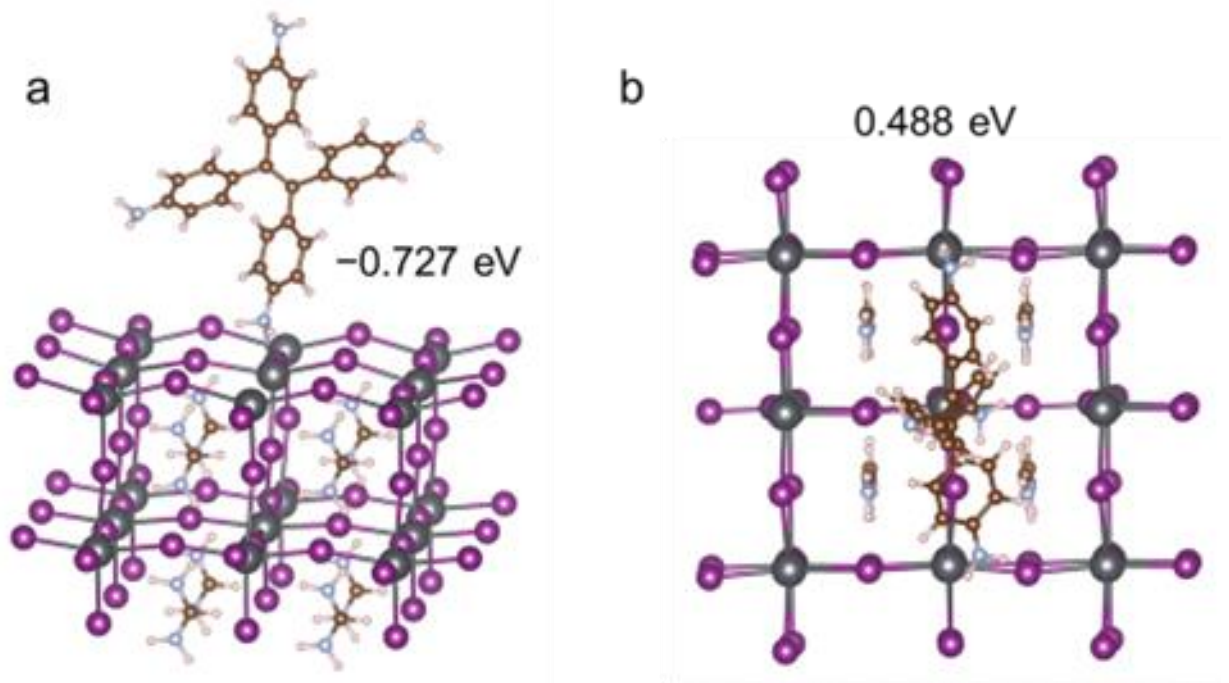

Figure R9. (a) DFT calculations of the interaction energy (E_{int}) of 4APE with V_I defect sites on the surface of perovskite. (b) DFT calculations of the formation energies of V_I defect sites within perovskite with 4APE.

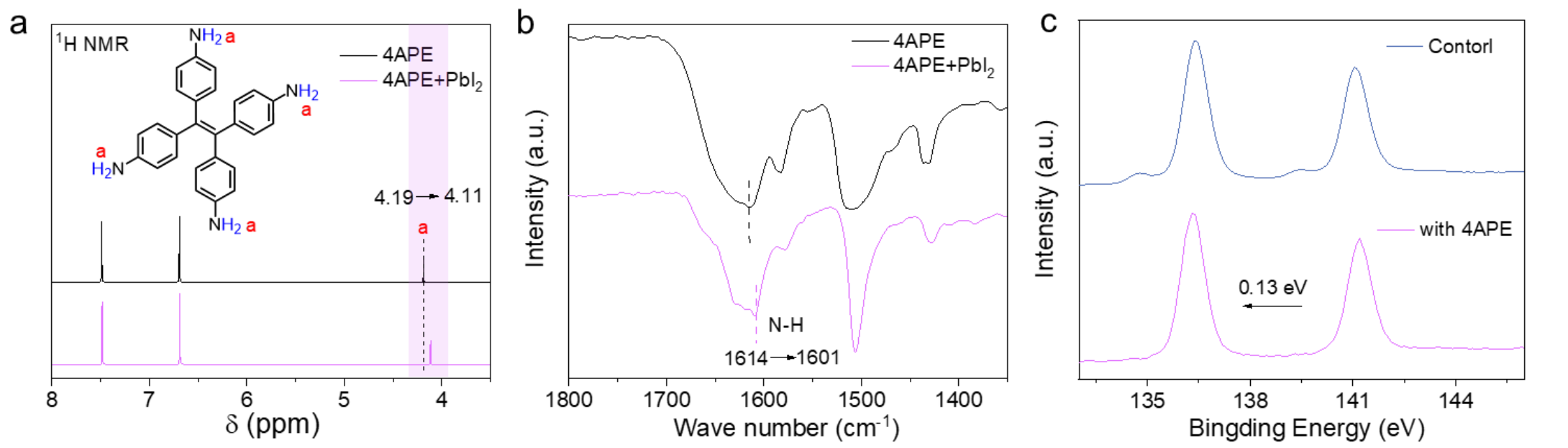

Figure R10. (a) ^{1}H NMR spectra of 4APE with and without PbI_2. (b) FTIR spectra of 4APE and PbI_2-4APE films. (c) Pb 4*f* XPS spectra of perovskite films without and with 4APE treatment.

2. Cross-sectional SEM images are currently absent for samples subjected to different molecular treatments. The authors should provide those data for all relevant samples to enable a systematic and detailed comparison of film morphology characteristics across the control and modified devices.

Response: We appreciate the reviewer's comments. Cross-sectional scanning electron microscopy (SEM) images of films treated with different molecules have been supplemented, as shown in Figure R11. The 4APM-treated perovskite film exhibits a more continuous grain structure with fewer and less distinct grain boundaries compared

to control and those treated with 2APM and 3APM.

To make this more clear, we added Figure R11 in the Supporting Information as Figure S17, and added these sentences in the manuscript:

"….In addition, cross-sectional SEM measurements further reveal that the 4APM-treated perovskite film exhibits a more continuous grain structure with fewer and less distinct grain boundaries compared to control and those treated with 2APM and 3APM (Figure S17)."

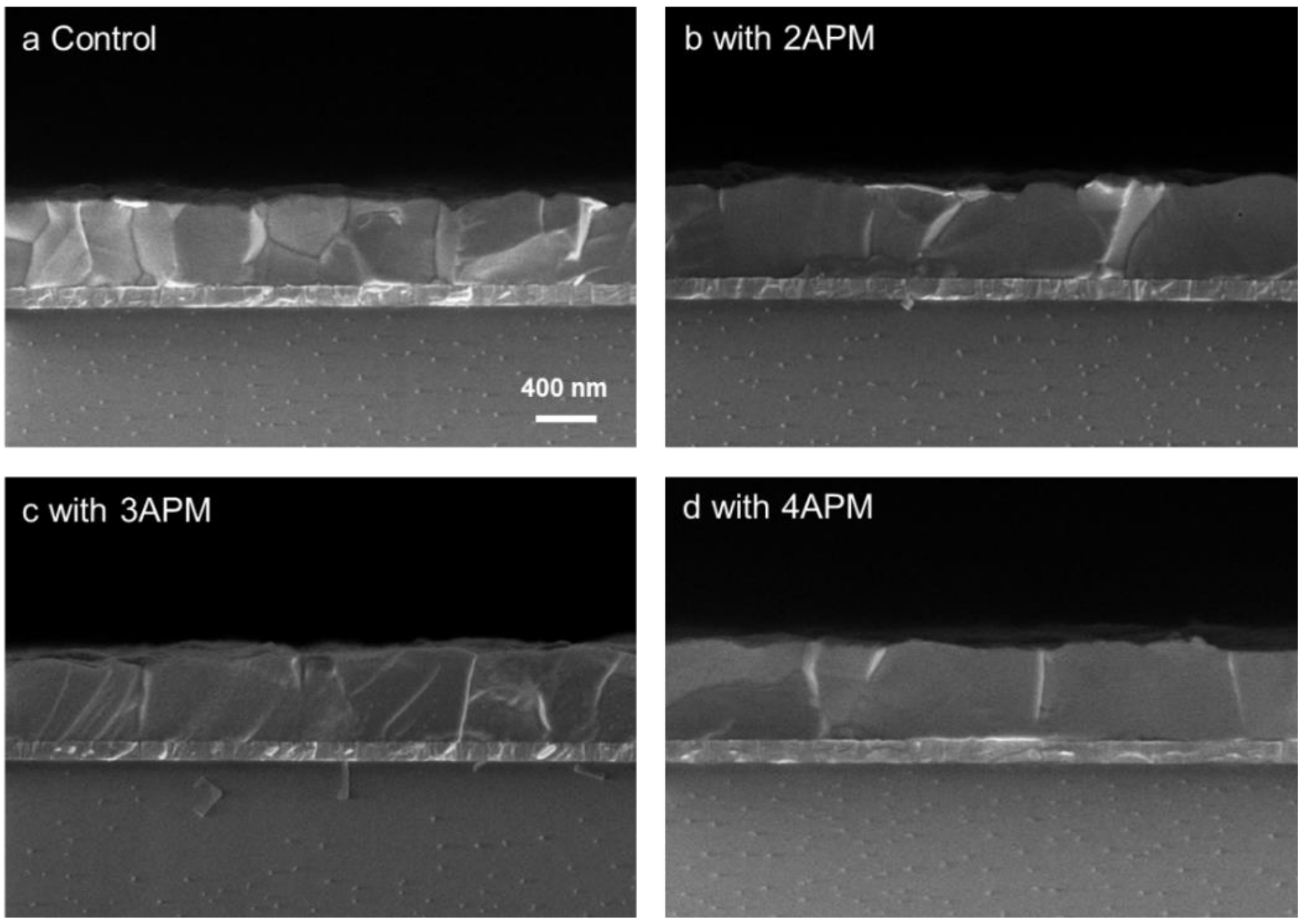

Figure R11. (a-d) Cross-sectional SEM images of perovskite films without and with 2APM, 3APM and 4APM, respectively.

3. Figure 1b shows significant differences in the calculated dipole moments among the three molecules, but the main text does not discuss the potential implications of these dipole moment variations for perovskite film formation or device physics.

Response: We thank the reviewer for this valuable suggestion. As presented in Figure 1b, the molecular dipole moment increases systematically with spatial distribution of amino groups, 0.72 D for 2APM, 0.75 D for 3APM, and 1.95 D for 4APM. A larger molecular dipole moment reflects more pronounced intramolecular charge separation, which strengthens electrostatic interactions with the perovskite surface, promoting more intense charge redistribution at the molecule/perovskite interface. (*Nat. Energy*, 2023, 8, 610-620) Furthermore, this is directly reflected in the most uniform surface potential distribution observed in KPFM measurements for 4APM-treated films, arising from the surface dipoles introduced by 4APM and the consequent suppression of surface potential fluctuations and reduction in surface defect density. (*Nat. Commun.*

2017, 8, 613; *Adv. Mater.* 2024, 36, 2310080)

To make this more clear, we added these sentences in the manuscript:

"……rising from 0.72 D for 2APM to 0.75 D for 3APM and 1.95 D for 4APM, which indicates that the 3D spatial configuration of amino groups plays a significant role in enhancing the molecular dipole moment, thereby strengthening the electrostatic interaction with perovskite surface and promoting charge redistribution at the molecule/perovskite interface. [25]"

"……Notably, the large surface potential fluctuations associated with grain boundaries in the untreated film are nearly eliminated after 4APM treatment, yielding a highly uniform surface potential distribution. This improvement can be attributed to the surface dipoles introduced by 4APM, which suppress potential fluctuations and reduce surface defect density. [41-44]…."

4. In Figure 5g, the voltage generated by the MEDE-modified device under 1 sun illumination appears inconsistent with the corresponding open-circuit voltage reported in the J–V curve. Please explain the reason for this discrepancy.

Response: We thank the reviewer for raising this issue. The unexpectedly low voltage in the original light intensity-dependent curves was attributable to batch-to-batch variations in device fabrication. The light intensity-dependent V_{OC} measurements have been repeated for the optimized 2APM-treated device, and the updated results are presented in Figure R12. Based on linear fitting of the V_{OC} versus light intensity data, the corrected ideality factor of the 2APM-treated device is determined to be 1.67.

To make this more clear, we updated Figure R12 in the manuscript as Figure 5g, and modified these sentences in the manuscript:

"……Consistently, the ideality factors (n_{id}) extracted from voltage response fitting decrease systematically with increasing the spatial distribution of amino groups, from 1.82 for the control to 1.67, 1.48, and 1.37 for 2APM-, 3APM-, and 4APM-based devices (Figure 5g),"

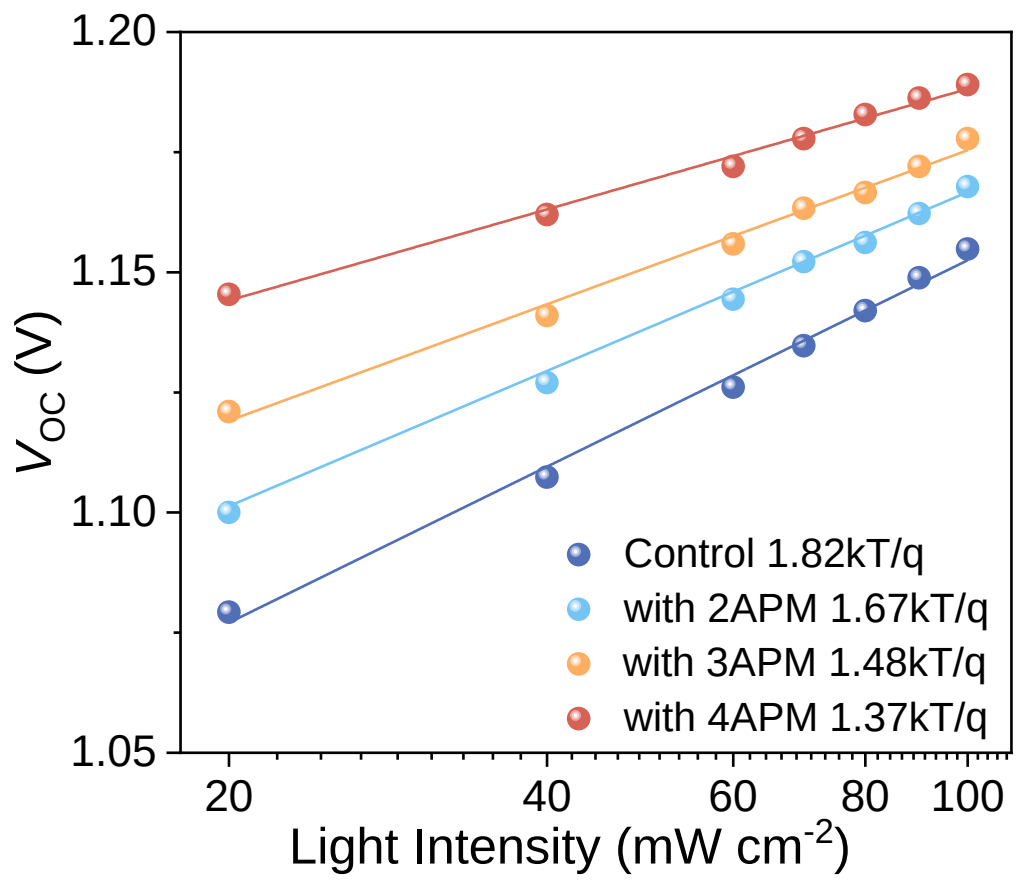

Figure R12. The light-intensity dependence of V_{OC} for PSCs.

5. Although a champion PCE of 26.26% is reported for the PSC, neither the main text nor the Supplementary Information specifies the device area or the active area used for this measurement. The authors need to provide the relevant information as well as the aperture mask used during characterization.

Response: We thank the reviewer for pointing out this important missing information. The active area of the tested cells is 0.25 cm^2 (0.5 cm × 0.5 cm), and a black metal mask with an aperture area of 0.09 cm^2 was applied during *J–V* characterization.

To make this more clear, we added these sentences in Device Characterization Section of the Supporting Information:

"…. The cells with an active area of 0.25 cm^2 (0.5 cm × 0.5 cm) were masked with a black metal mask with an area of 0.09 cm^2."

Reviewer #3 (Remarks to the Author):

In this manuscript, Jiang et al. reported three aniline-based molecules, so-called 4,4'-methylenedianiline (MEDE), tris(4-aminophenyl)methane (TSAM), and tetra(4-aminophenyl)methane (TAAM), with different spatial configurations as in-situ modulators incorporated into the perovskite precursor solution. In the three molecules, TAAM with a 3D architecture most effectively suppresses uncoordinated Pb^{2+} defects, enhances the perovskite film crystallinity, and optimizes the interfacial energy-level alignment between the perovskite and the HTL. The TAAM-incorporated small-area perovskite solar cells and mini-modules achieved efficiencies of 26.26% and 23.16%, respectively, along with notable improvement in operational stability. This work can be considered for publication in Nature Communications after the points listed below are addressed.

We sincerely appreciate the reviewer's positive and constructive comments, which have greatly contributed to improving the quality of our manuscript.

1. The title of this manuscript needs to be revised to clearly show the specific difference in the spatial configuration of the three molecules.

Response: We appreciate the reviewer for raising this point. The title of this manuscript has been revised to "Three-dimensional distribution of amino groups improves perovskite crystallization and defect passivation in high-performance photovoltaics"

2. The abbreviations of the three molecules, which do not reflect their structural characteristics, are suggested to be changed.

Response: We thank the reviewer for pointing this out. The abbreviations of the three molecules have been renamed to bis(4-aminophenyl)methane (2APM), tris(4-aminophenyl)methane (3APM), and tetrakis(4-aminophenyl)methane (4APM), with corresponding revisions made throughout the manuscript, such as "….. Here, we introduce three phenylamine-based molecules, bis(4-aminophenyl)methane (2APM), tris(4-aminophenyl)methane (3APM) and tetrakis(4-aminophenyl)methane (4APM), featuring distinct spatial geometries…" in the Abstract, and "….. In this work, we investigate the effect of spatial distribution of amino groups in passivators on their passivation capability and on PSC performances by comparing bis(4-aminophenyl)methane (2APM), tris(4-aminophenyl)methane (3APM), and tetrakis(4-aminophenyl)methane (4APM)…." in the Introduction.

3. The NMR peak shifts of the amino groups in the three aniline molecules are described as “shifted upfield”. However, in Figures 2a-c and Figure S5, the chemical shifts of the amino groups consistently move toward lower numerical values. Please clarify how the direction of the shift is defined.

Response: We appreciate the reviewer for raising this point. Our description of the chemical shifts (δ) as "shifted upfield" is entirely consistent with the observed decrease in δ values in Figures 2a–c and Figure S6. In ^{1}H NMR spectroscopy, the chemical shift scale is referenced to tetramethylsilane (TMS) at 0 ppm, with upfield shifts corresponding to smaller δ values and reflecting increased nuclear shielding. Upon mixing with PbI_2, the $–NH_2$ proton resonances of 2APM, 3APM, and 4APM all shift toward smaller δ values (by −0.04, −0.07, and −0.10 ppm, respectively), confirming coordination between the lone pair electrons on the amino nitrogen and Pb^{2+} ions. This coordination drives electron density toward Pb^{2+}, increasing the relative electron density around the neighboring $–NH_2$ protons, enhancing their shielding, and consequently shifting their resonance signals upfield.

4. In Figure 2f, the N–H peak shift does not appear to correspond to the value of “$\Delta = 14\ cm^{-1}$” stated in the main text. The authors should verify whether this discrepancy reflects a labeling error in the figure or an inaccuracy in the reported value and provide corrected data or a revised figure as appropriate.

Response: We thank the reviewer for the careful and thorough review of our manuscript. Upon re-examination of the raw data, we confirm that the N–H bending vibration peak of 4APM shifts by 16 cm^{-1} (from 1616 cm^{-1} to 1600 cm^{-1}), and the previously reported value of "$\Delta = 14\ cm^{-1}$" in the original manuscript was indeed a typographical error. The value has been corrected to $\Delta = 16\ cm^{-1}$ in the revised manuscript:

“…4APM exhibited the most pronounced red-shifts (N–H bending: 1616 cm^{-1} → 1600 cm^{-1}, $\Delta = 16\ cm^{-1}$; N–H stretching: 3429 cm^{-1} → 3413 cm^{-1}, $\Delta = 16\ cm^{-1}$),…”

5. It is stated that all three aniline molecules are incorporated as bulk-phase additives within the perovskite film. However, both AFM-IR and TOF-SIMS analyses show strong signals from these molecules on the film surface. Please explain the cause of this surface signal.

Response: We thank the reviewer for this thoughtful and important question. Although the three aniline molecules are introduced as bulk-phase additives uniformly dispersed in the perovskite precursor solution, dynamic redistribution occurs during spin-coating, crystallization, and annealing, resulting in preferential surface and grain boundary

enrichment.

During perovskite crystallization, uncoordinated additive molecules are expelled from the growing perovskite lattice and accumulate at interfacial regions, particularly the air/film interface (top surface) and grain boundaries (*Adv. Energy Mater.* e04647 (2025).; *ACS Energy Lett.* 10, 3325–3334 (2025)). Furthermore, as demonstrated in this work through DFT simulations, NMR, and FTIR characterizations, the–NH_2 groups of these molecules exhibit strong binding affinity for under-coordinated Pb^{2+} ions. Since the top surface and grain boundaries of perovskite films host the highest density of such under-coordinated Pb^{2+} defect sites, the expelled molecules preferentially anchor to these regions through coordination interactions, effectively passivating surface defects.

The strong and spatially uniform benzene ring signals observed in AFM-IR imaging, together with the surface-concentrated molecular signals in TOF-SIMS depth profiling, are therefore consistent with this synergistic accumulation mechanism, driven by both physical segregation during crystallization and chemical affinity for surface defects.

To make this more clear, we added and modified these sentences in the manuscript:

"…… Depth-profile analysis reveals that the additive molecules are partially enriched at the upper surface of the perovskite layer (Figure S22), owing to the synergistic effects of physical segregation during perovskite crystallization and molecular chemical affinity for surface defects, which favors effective passivation of under-coordinated Pb^{2+} defects at the surface and grain boundaries of perovskite films. [45, 46]"

6. The team "PEC" in the sentence "retaining 93% of their initial PEC after 2000 h of aging" appears to be an error. The authors should carefully check the entire manuscript to identify and correct any similar typographical or terminology issues.

Response: We thank the reviewer for the careful and thorough review of our manuscript and for identifying this typographical error. The term "PEC" in the sentence "retaining 93% of their initial PEC after 2000 h of aging" is indeed a typo for "power conversion efficiency (PCE)" and has been corrected accordingly. The entire manuscript has been carefully proofread to identify and rectify any similar typographical or terminology inconsistencies, and all necessary corrections have been made in the revised version.

"……The 4APM-modified PSCs exhibit the best durability, retaining 93% of their initial PCE after 2000 h of aging (Figure 5h), substantially outperforming the 2APM- (85%) and 3APM-based (88%) devices over the same period,…."

7. The reduced surface potential variation observed in KPFM measurement is

interpreted as evidence of lower defect density. However, KPFM signals are inherently sensitive not only to defect states but also to local work function variations and surface dipole moments.

Response: We appreciate the reviewers for pointing this out. We fully acknowledge that KPFM signals are inherently sensitive not only to defect states but also to local work function variations and surface dipole moments. For this reason, we wish to clarify that the KPFM data presented in Figures 4e–h are not interpreted in isolation, but are corroborated by a comprehensive set of complementary characterization results.

Specifically, FTIR and XPS measurements confirm strong coordination interactions between the $–NH_2$ groups of the additive molecules and under-coordinated Pb^{2+} defects, directly evidencing effective defect passivation. PL and PLQY measurements show that 4APM-treated films exhibit the highest values, demonstrating effective suppression of non-radiative recombination. TRPL further reveals that 4APM-treated films possess the longest carrier lifetime, directly confirming a significant reduction in defect-assisted recombination.

We also acknowledge that surface enrichment of additive molecules, as evidenced by TOF-SIMS and AFM-IR, may introduce additional interface dipoles that contribute to the observed KPFM signals. (*Nat Commun.* 2017*,* 8, 613; *Adv. Mater.* 2024, 36, 2310080.) However, the key observation in Figure S20 is that the large potential fluctuations associated with grain boundaries in untreated films are nearly eliminated after 4APM treatment, yielding a highly uniform surface potential distribution. Such a dramatic reduction in spatial potential fluctuations is most consistently explained by the passivation of local defect states at grain boundaries and film surfaces. (*ACS Nano* 2019, 13, 12127; *Nature* 2022, 611, 278.)

To make this more clear, we added and modified these sentences in the manuscript:

"…... Notably, the large surface potential fluctuations associated with grain boundaries in the untreated film are nearly eliminated after 4APM treatment, yielding a highly uniform surface potential distribution. This improvement can be attributed to the surface dipoles introduced by 4APM, which suppress potential fluctuations and reduce surface defect density. [41-44]"

8. Please also clarify how the phenylamine-based molecules contribute to the observed increase in V_{OC}. Specifically, it should be clarified whether the V_{OC} enhancement arises from the suppressed non-radiative recombination, the modified interfacial energy-level alignment, or their synergistic effects.

Response: We thank the reviewer for this insightful comment. The enhancement in V_{OC} observed in additive-treated devices arises from the synergistic contributions of suppressed non-radiative recombination and optimized interfacial energy-level alignment. Multiple complementary measurements consistently demonstrate that phenylamine-based molecules effectively suppress trap-assisted non-radiative recombination. As shown in Figure 3k, the quasi-Fermi level splitting (QFLS) increases systematically from 1.166 V in the control to 1.220 V in 4APM-treated devices, directly confirming that non-radiative recombination losses are most effectively mitigated by 4APM treatment. Furthermore, Figure 5g shows that the ideality factor decreases with increasing spatial distribution of amino groups, indicating effective defect passivation.

As shown in Figure 3l, 4APM treatment induces a positive shift in the valence band maximum (VBM) of the perovskite layer, optimizing the energy-level alignment at the perovskite/hole transport layer (HTL) interface. This reduced energy barrier facilitates more efficient hole extraction from the perovskite to the HTL, contributing directly to the enhanced V_{OC}. (*Nat. Energy* 2023, 8, 515–525.)

To make this more clear, we added these sentences in the manuscript:

"….. Notably, the 3D molecule 4APM delivers the highest PCE of 26.26%, with a V_{OC} of 1.187 V, J_{SC} of 25.72 mA cm^{-2}, and FF of 85.95%. The progressive enhancement in V_{OC} across the additive-treated devices can be attributed to the synergistic effects of suppressed non-radiative recombination and optimized interfacial energy level alignment. [48]"

Reviewer #4 (Remarks to the Author):

This study finds that the three-dimensional (3D) molecule TAAM, due to its multidentate aniline groups, most effectively suppresses uncoordinated Pb^{2+} defects and improves the crystallinity of perovskite films. Furthermore, the TAAM molecule demonstrates good device performance in both perovskite solar cells and modules (14 cm^2) with superior operational stability under MPP tracking conditions. These findings provide an important reference for the rational selection of in-situ modification molecules in high-performance perovskite photovoltaics. Therefore, I am willing to recommend its publication in this journal after addressing the following revisions.

We sincerely appreciate the reviewer's thoughtful and constructive suggestions, which have improved the clarity and quality of our manuscript.

1. The paper indirectly infers that TAAM effectively suppresses uncoordinated Pb^{2+} defects based on results such as improved XRD crystallinity and homogenized surface potential in KPFM. However, it lacks direct quantitative characterization of changes in trap density. It is recommended to supplement tests such as space-charge-limited current (SCLC) to quantitatively compare the trap densities of devices modified with different molecules.

Response: We thank the reviewer for this valuable comment. To directly and quantitatively assess the trap density in perovskite films treated with different additives, space charge limited current (SCLC) measurements were performed. As shown in Figure R13, the trap state density of the pristine control film is 9.39×10^{15} cm^{-3}, which decreases progressively to 7.12×10^{15} cm^{-3}, 5.05×10^{15} cm^{-3}, and 3.71×10^{15} cm^{-3} upon treatment with 2APM, 3APM, and 4APM, respectively. This reduction in trap density with increasing spatial distribution of amino groups directly confirms that 4APM affords the most effective suppression of under-coordinated Pb^{2+} defects, consistent with the trend in device performance observed across the series.

To make this more clear, we added Figure R13 in the Supporting Information as Figure S27, and added and modified these sentences in the manuscript:

"……The trap state density in perovskite films was quantitatively assessed using the space charge limited current (SCLC) method. As shown in Figure S27, the pristine film exhibits a trap state density of 9.39×10^{15} cm^{-3}, which decreases progressively to 7.12×10^{15} cm^{-3}, 5.05×10^{15} cm^{-3}, and 3.71×10^{15} cm^{-3} upon treatment with 2APM, 3APM, and 4APM, respectively, demonstrating the best defect passivation capability of 4APM."

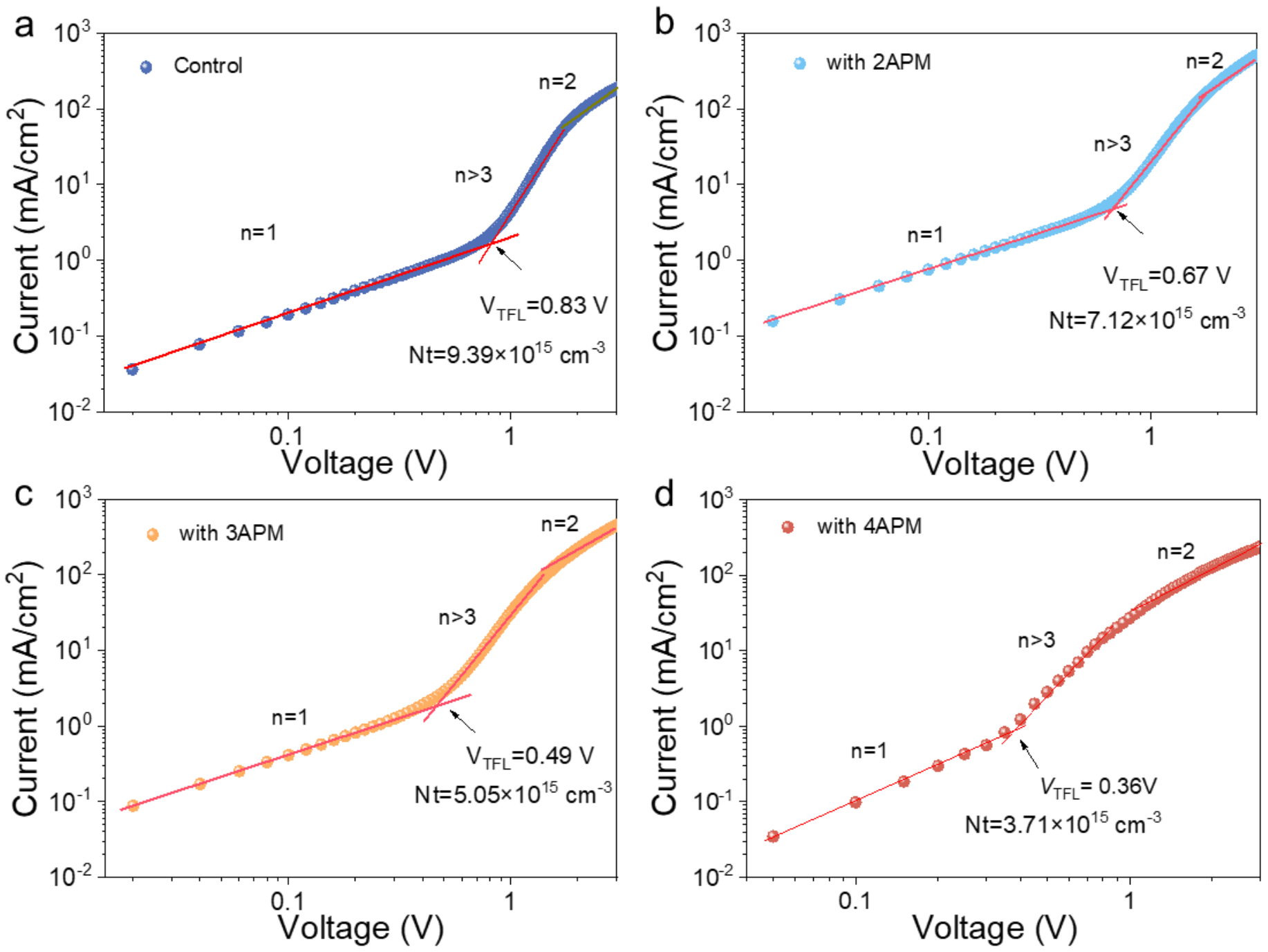

Figure R13. Space charge limited current (SCLC) curve of hole-only devices; V_{TFL} values indicate trap density variation.

2. The paper compares three molecules—MEDE, TSAM, and TAAM—with functional group (-NH2) counts of 2, 3, and 4, respectively. Please explain how the authors more fairly compare the influence of "spatial configuration" rather than merely the influence of the "number of functional groups."

Response: We sincerely appreciate the reviewer's thoughtful and constructive suggestions. To further elucidate the respective contributions of functional group number and molecular spatial configuration to passivation capability, we selected a molecule with a more planar amino group distribution, tetrakis(4-aminophenyl)ethylene (4APE), bearing four phenylamine groups, as an additional reference to investigate its interaction with lead defects. Based on electrostatic potential (ESP) calculations (Figure R8), the amino groups in 4APE exhibit an electron cloud density of −0.264, significantly higher than that of the three-dimensional molecule 4APM, indicating that the enhanced electron cloud density arises from cooperative interactions among multiple functional groups rather than from the three-dimensional configuration alone. Nevertheless, the molecular dipole moment of 4APE (0.64 D) is substantially lower than that of 4APM (1.95 D), demonstrating that the three-dimensional spatial configuration plays a decisive role in elevating the molecular dipole moment. Furthermore, 4APE exhibits an interaction energy of −0.727 eV and a defect formation energy of 0.488 eV with the perovskite surface (Figure R9), both of which are inferior to those of 4APM, demonstrating that despite possessing an equivalent

number of functional groups, the planar molecular configuration of 4APE results in weaker overall passivation capability compared to the three-dimensional architecture of 4APM.

The interaction between 4APE and lead defects was systematically investigated by ^1H NMR, FTIR, XPS, and SEM measurements. As shown in Figure R10a, the ^1H NMR chemical shift of the $-NH_2$ group in 4APE shifts upfield by 0.08 ppm upon addition of PbI_2, which is smaller than that observed for 4APM (0.10 ppm), indicating a weaker coordination interaction between the planar 4APE molecule and Pb^{2+}. Consistently, FTIR measurements reveal that upon mixing with PbI_2, the N–H bending vibration of 4APE red-shifts by 13 cm^{-1} (from 1614 to 1601 cm^{-1}), a smaller shift compared to 16 cm^{-1} for 4APM (Figure R10b). XPS analysis further shows that the Pb 4*f* binding energy shift induced by 4APE (0.13 eV) is smaller than that induced by 4APM (0.16 eV) (Figure R10c). These results indicate that while interactions among multiple functional groups enhance electron cloud density and interaction energy, the three-dimensional configuration of 4APM enables its four $-NH_2$ groups to extend more effectively in space, significantly increasing the molecular dipole moment and consequently affording superior defect passivation capability.

To make this more clear, we added the Figures R8-R10 in the Supporting Information as Figures S9-S11, and added and modified these sentences in the manuscript:

"……Furthermore, another molecule, tetrakis(4-aminophenyl)ethylene (4APE), is also studied as a reference, which possesses the same number yet relatively more planar distribution of amino groups compared to 4APM. [24] The study found that 4APM with its amino groups spatially distributed in a three-dimensional (3D) pattern, relative to the other three molecules, present more obvious enhancements in defect passivation, and crystallinity regulation.…."

"….. To decouple the influence of amino groups quantity from their spatial distribution, we introduced a more planar amino-based molecule 4APE as a structural control (Figures S9-S11). Compared to 4APM, 4APE exhibits a denser electron cloud distribution yet a significantly lower dipole moment, despite containing the same number of amino groups. Both computational and experimental results consistently demonstrate that while the number of amino groups contributes to the interaction energy, the 3D configuration of 4APM enables its four $-NH_2$ groups to extend more freely and uniformly into space, in favor of stronger and more spatially distributed interactions with perovskite defects. This stereoscopically structural advantage amplifies the molecular dipole moment of 4APM relative to the more planar 4APE, thereby improving its defect passivation capability beyond what the count of functional groups

alone can achieve.……”

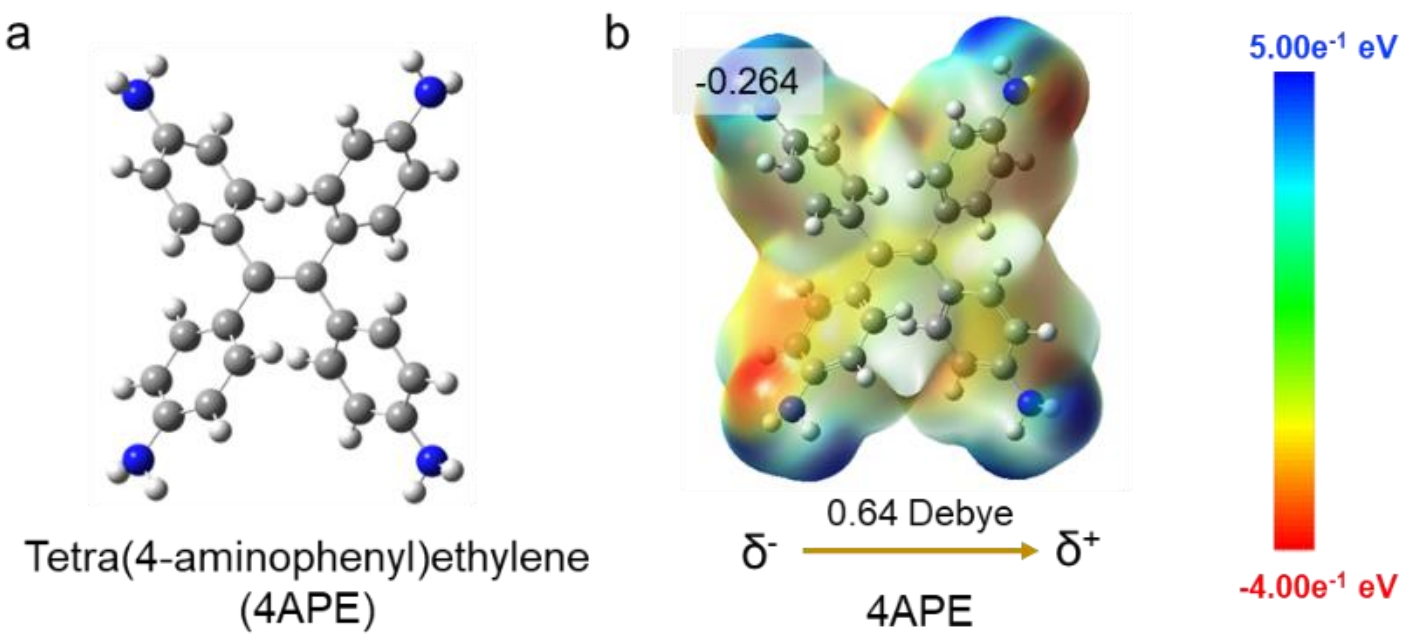

Figure R8. (a) molecular structure of 4APE. (b) Calculated ESP profiles for 4APE molecule.

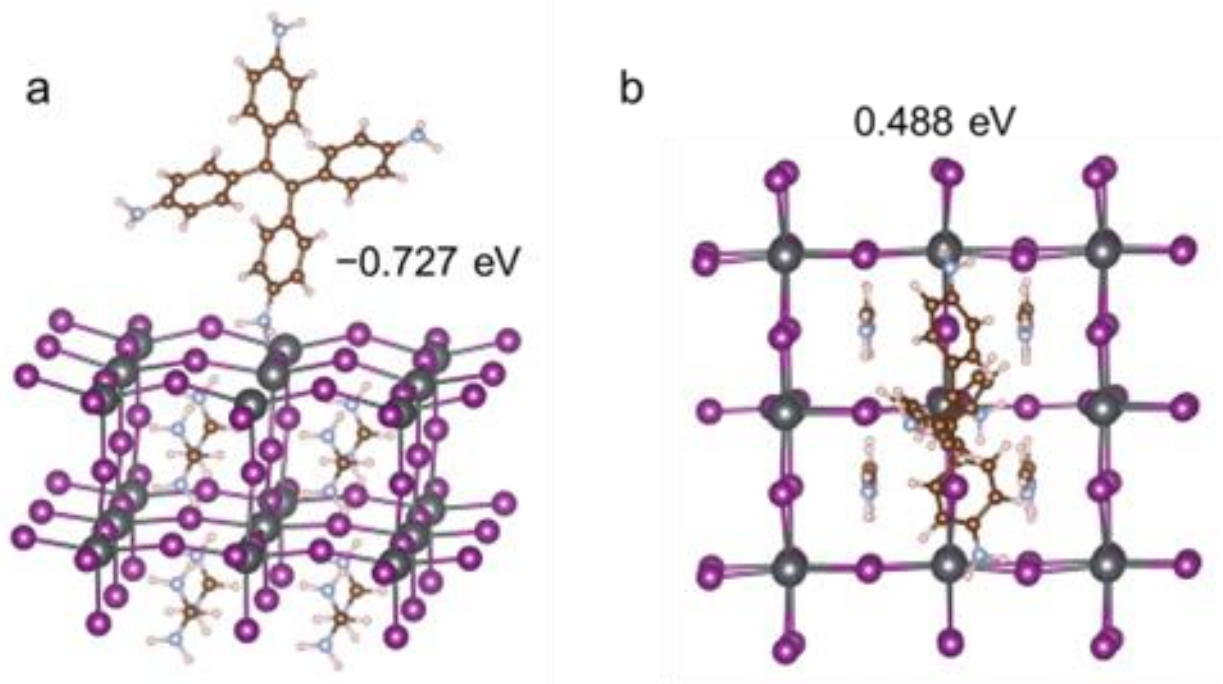

Figure R9. (a) DFT calculations of the interaction energy (E_{int}) of 4APE with V_I defect sites on the surface of perovskite. (b) DFT calculations of the formation energies of V_I defect sites within perovskite with 4APE.

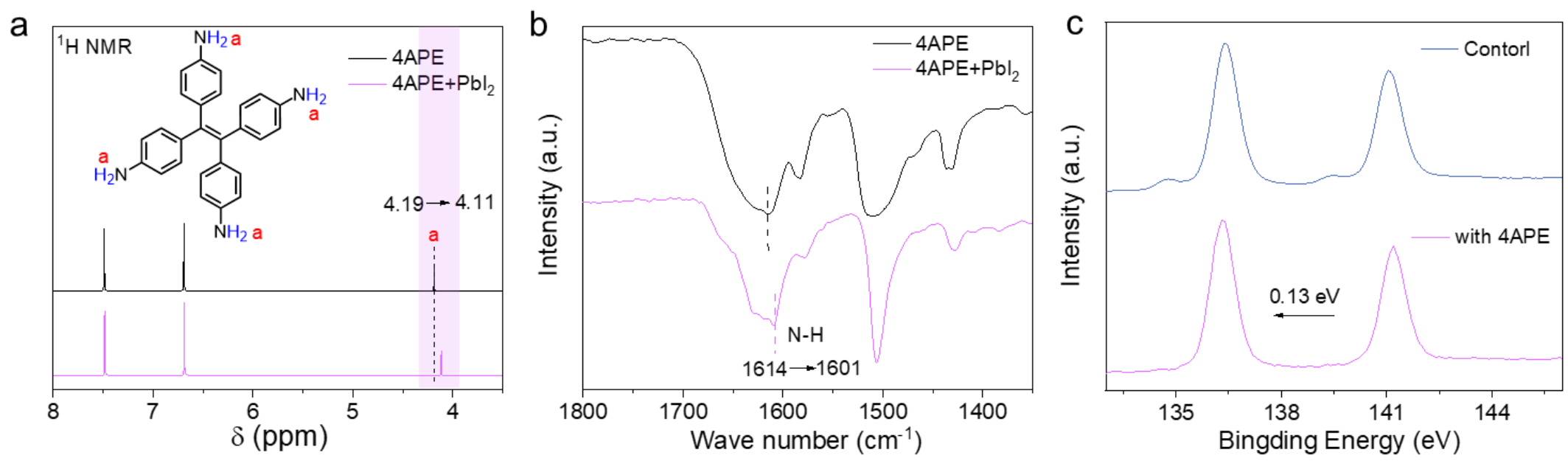

Figure R10. (a) ^{1}H NMR spectra of 4APE with and without PbI_2. (b) FTIR spectra of 4APE and PbI_2-4APE films. (c) Pb 4*f* XPS spectra of perovskite films without and with 4APE treatment.

3. The authors optimized the concentrations of all three additives and achieved the best device performance with the concentration of 0.1 mg/ml. Please explain why the higher or lower concentrations cause the inferior device performance.

Response: We thank the reviewer for raising this important question. Through systematic concentration optimization, 0.1 mg/mL was identified as the optimal concentration for all three additives, as detailed in Figures S24a–c and Tables S3–S5. The concentration dependence of device performance can be rationalized as follows.

At lower concentrations, an insufficient number of additive molecules is available to effectively passivate the abundant defect sites, particularly under-coordinated Pb^{2+} at film surfaces and grain boundaries, resulting in a higher density of non-radiative recombination centers and consequently suboptimal V_{OC} and FF. Conversely, at higher concentrations, the excessive additive molecules disrupt the chemical environment of the perovskite precursor solution and interfere with the crystallization process, compromising film quality, introducing unwanted organic impurity phases, leading to inferior device performance.

The optimal concentration of 0.1 mg/mL therefore strikes the most favorable balance between sufficient defect passivation and minimal disruption to perovskite crystallization, affording the best overall device performance.

To make this more clear, we added these sentences in the Supporting Information under Figure S24:

"At lower concentrations, an insufficient number of additive molecules is available to effectively passivate the abundant defect sites, particularly under-coordinated Pb^{2+} at film surfaces and grain boundaries, resulting in a higher density of non-radiative recombination centers and consequently suboptimal V_{OC} and FF. Conversely, at higher concentrations, the excessive additive molecules disrupt the chemical environment of the perovskite precursor solution and interfere with the crystallization process, compromising film quality, introducing unwanted organic impurity phases, leading to inferior device performance. The optimal concentration of 0.1 mg/mL therefore strikes the most favorable balance between sufficient defect passivation and minimal disruption to perovskite crystallization, affording the best overall device performance."

4. The differential charge distribution of the 3D molecule TAAM shows significantly enhanced charge transfer performance. Please explain the reason for this enhancement.

Response: We thank the reviewer for this insightful question. The enhanced charge transfer performance of 4APM originates from two intrinsic electronic advantages conferred by its three-dimensional distribution of amino groups.

As shown in Figure 1b, the four $–NH_2$ groups in 4APM results in the highest electron cloud density among the three molecules. This elevated electron cloud density enhances the ability of the $–NH_2$ groups to donate electron density toward under-coordinated Pb^{2+} sites through coordination interactions, thereby facilitating more efficient charge sharing at the perovskite surface.

The three-dimensional configuration of 4APM gives rise to the largest molecular dipole

moment among the additives (1.95 D, compared to 0.75 D for 3APM and 0.72 D for 2APM). The enhanced dipole moment strengthens electrostatic interactions with the perovskite surface, further promoting charge redistribution at the molecule/perovskite interface. (Nature Energy 2023, 8, 610–620.)

To make this more clear, we added these sentences in the manuscript:

"……which indicates that the 3D spatial configuration of amino groups plays a significant role in enhancing the molecular dipole moment, thereby strengthening the electrostatic interaction with perovskite surface and promoting charge redistribution at the molecule/perovskite interface.[25]…. "

"…. Charge density difference plots further reveal that 4APM facilitates greater electron density sharing toward Pb^{2+} relative to the other additives (Figures 1d and S5)…."

5. The author mentioned in the discussion that the molecules were incorporated into the perovskite bulk phase, but the TOF-SIMS results clearly show strong signals of the molecules still present on the film surface. Please explain why this phenomenon occurs and provide a clearer description of the actual distribution of the molecules.

Response: We thank the reviewer for this thoughtful and important question. Although the three aniline molecules are introduced as bulk-phase additives uniformly dispersed in the perovskite precursor solution, dynamic redistribution occurs during spin-coating, crystallization, and annealing, resulting in preferential surface and grain boundary enrichment.

During perovskite crystallization, uncoordinated additive molecules are expelled from the growing perovskite lattice and accumulate at interfacial regions, particularly the air/film interface (top surface) and grain boundaries (Adv. Energy Mater. e04647 (2025).; ACS Energy Lett. 10, 3325–3334 (2025)). Furthermore, as demonstrated in this work through DFT simulations, NMR, and FTIR characterizations, the–NH_2 groups of these molecules exhibit strong binding affinity for under-coordinated Pb^{2+} ions. Since the top surface and grain boundaries of perovskite films host the highest density of such under-coordinated Pb^{2+} defect sites, the expelled molecules preferentially anchor to these regions through coordination interactions, effectively passivating surface defects.

To make this more clear, we added and modified these sentences in the manuscript:

"……Depth-profile analysis reveals that the additive molecules are partially enriched

at the upper surface of the perovskite layer (Figure S22), owing to the synergistic effects of physical segregation during perovskite crystallization and molecular chemical affinity for surface defects, which favors effective passivation of under-coordinated Pb^{2+} defects at the surface and grain boundaries of perovskite films. [45, 46]"

6. Why did the authors choose silver as the electrode instead of gold? I am curious whether there might also be some differences in photovoltaic parameters between the two, and I would like the authors to provide some comparisons and reasonable explanations.

Response: We thank the reviewer for this thoughtful suggestion. The photovoltaic performance of devices fabricated with silver and gold electrodes was systematically compared, and the results are summarized in Table R2. Devices employing gold electrodes of equivalent thickness exhibit a slightly lower average V_{OC} compared to those with silver electrodes, while no significant differences are observed in J_{SC} or FF. This V_{OC} reduction can be attributed to the tendency of gold to migrate into the perovskite active layer and form deep-level defect states, which promote non-radiative recombination (*ACS Nano* **10**, 6306–6314 (2016).; *Energy Environ. Sci.* **18**, 10483-10493 (2025).) Additionally, silver electrodes offer practical advantages in terms of cost-effectiveness and processability.

Table R2. Comparison of the photovoltaic performance of 20 devices for Control, 2APM-, 3APM- and 4APM-treated samples. (active area: 0.09 cm^2)

Sample		V_{OC} (V)	J_{SC} (mA cm^{-2})	*FF* (%)	PCE (%)
Control	Ag	1.153±0.0033	25.35±0.13	80.75±1.07	23.61±0.37
	Au	1.142±0.0044	25.36±0.24	80.68±1.57	23.36±0.45
with 2APM	Ag	1.161±0.0035	25.42±0.14	82.05±0.72	24.21±0.35
	Au	1.151±0.0039	25.39±0.22	82.11±1.02	23.99±0.42
with 3APM	Ag	1.173±0.0034	25.56±0.11	84.21±0.64	25.24±0.34
	Au	1.165±0.0041	25.54±0.26	84.12±0.94	25.02±0.38
With 4APM	Ag	1.184±0.0032	25.63±0.08	85.37±0.55	25.91±0.27
	Au	1.177±0.0037	25.61±0.23	85.25±0.75	25.69±0.32

7. Please carefully review the grammar throughout the manuscript and thoroughly proofread the spelling to ensure accuracy and consistency. It would be greatly appreciated if the authors could pay close attention to any typographical errors, awkward phrasing, or grammatical inconsistencies, and make the necessary revisions to improve the overall clarity and professionalism of the manuscript.

Response: We sincerely thank the reviewer for this careful and constructive suggestion. The entire manuscript has been thoroughly proofread and revised to address typographical errors, grammatical inconsistencies, and awkward phrasing. In addition, all specific issues raised by the reviewers, including the typographical error "PEC" (corrected to "PCE") and inconsistencies in figure data descriptions, have been carefully addressed. We are confident that these revisions have substantially improved the overall clarity, accuracy, and professionalism of the manuscript.
